# Diradical reaction mechanisms in [3 + 2]-cycloadditions of hetaryl thioketones with alkyl- or trimethylsilyl-substituted diazomethanes

**DOI:** 10.3762/bjoc.12.71

**Published:** 2016-04-14

**Authors:** Grzegorz Mlostoń, Paulina Pipiak, Heinz Heimgartner

**Affiliations:** 1 Department of Organic and Applied Chemistry, University of Łódź, Tamka 12, PL 91-403 Łódź, Poland; 2 Department of Chemistry, University of Zürich, Winterthurerstrasse 190, CH-8057 Zürich, Switzerland

**Keywords:** [3 + 2]-cycloadditions, diazoalkanes, diradicals, 1,3-dithiolanes, reaction mechanisms, thioketones

## Abstract

Reactions of dihetaryl and aryl/hetaryl thioketones with 2-diazopropane, diazoethane, and (trimethylsilyl)diazomethane were studied at variable temperature. The experiments showed that reactions with 2-diazopropane carried out at –75 °C occur mainly via the initially formed, relatively stable 1,3,4-thiadiazolines as products of the [3 + 2]-cycloaddition of the diazo dipole onto the C=S bond. The latter decompose only at higher temperature (ca. −40 °C) to generate thiocarbonyl *S*-isopropanide. In the absence of the starting thioketone, the corresponding thiiranes and/or ethene derivatives, formed from them via spontaneous desulfurization, are the main products. In contrast, reactions with diazoethane occurred predominantly via initially formed diradicals, which in cascade processes gave sterically crowded 4,4,5,5-tetrahetaryl-1,3-dithiolanes as major products. Finally, the reaction of dihetaryl thioketones with (trimethylsilyl)diazomethane occur smoothly at −75 °C leading to the corresponding 4,4,5,5-tetrahetaryl-1,3-dithiolanes as the exclusive [3 + 2]-cycloadducts formed via a cascade of postulated diradicals. The presence of S or Se atoms in the hetaryl rings is of importance for stabilizing diradical intermediates. Remarkably, in no single case, the ‘head-to-head dimerization’ of aryl/hetaryl and dihetaryl substituted thiocarbonyl ylides was observed.

## Introduction

Cycloaddition reactions belong to the most important classes of organic reactions, and [3 + 2]- cycloadditions, also known as 1,3-dipolar cycloadditions or Huisgen reactions, offer a universal tool for the preparation of five-membered heterocycles with a variable number of heteroatoms in the ring [1–2]. In addition to their practical importance, discussions on the mechanism contribute significantly to the development of fundamental concepts in organic chemistry [3–7]. The first general concept of concerted [3 + 2]-cycloadditions was formulated by Huisgen [4]. However, some time later, Huisgen and co-workers reported stepwise [3 + 2]-cycloadditions via zwitterionic intermediates [8–9]. Large differences of energies of the frontier orbitals of dipole and dipolarophile, as well as sterically demanding groups at the terminus of the dipole, were pointed out as requirements for the initiation of the ‘zwitterionic pathway’. In addition to the experimental findings, new reports dealing with computational studies aimed at the demonstration of new zwitterionic [3 + 2]-cycloadditions were published [10–12]. Finally, a third concept for the interpretation of the mechanism of [3 + 2]-cycloadditions, formulated by Firestone, is based on the assumption that they occur via diradical intermediates [13–15].

**Scheme 1:**
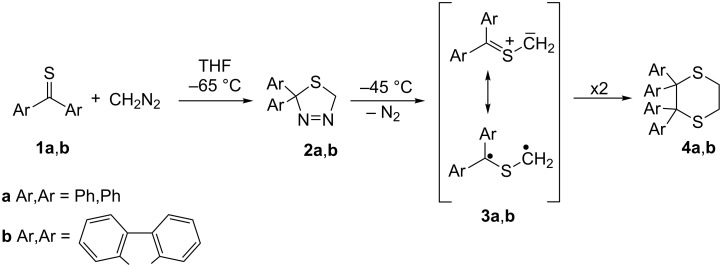
‘Head-to-head dimerization’ of diarylthioketone *S*-methanides **3a**,**b** leading to 2,2,3,3-tetrasubstituted 1,4-dithianes **4a**,**b**.

Reactions of aromatic thioketones with diazomethane are well established. For example, in the case of thiobenzophenone (**1a**), the reaction performed at –65 ºC occurs without evolution of N_2_ and the in situ formed 2,2-diphenyl-1,3,4-thiadiazoline **2a** can be subsequently used as a precursor of the reactive thiobenzophenone *S*-methanide (a thiocarbonyl ylide) **3a** at ca. −45 °C, when the evolution of N_2_ takes place [16–20]. An analogous course of the reaction with diazomethane was observed in the case of thiofluorenone (**1b**, Scheme 1).

When the decomposition of **2a** or **2b** was performed in the presence of a suitable dipolarophile, the corresponding [3 + 2]-cycloadducts were formed, whereas in the absence of a dipolarophile, the ‘head-to-head dimerization’ leading to 2,2,3,3-tetraaryl-1,4-dithianes **4a**,**b** is the exclusive reaction.

**Scheme 2:**
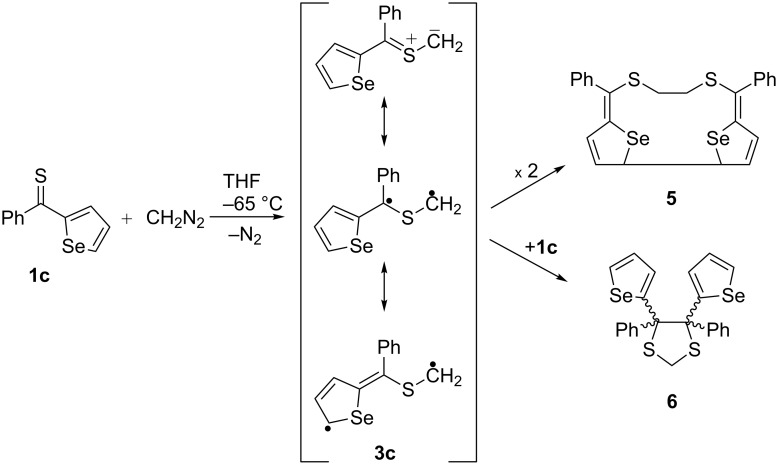
Diradical nature of the reactive intermediate **3c** in the reaction of phenyl selenophen-2-yl thioketone **1c** with diazomethane.

Heteroatoms such as S and Se are known to stabilize radical centers [21]. In our ongoing studies on thioketones and their applications in the cycloaddition chemistry, we described in a recent publication the unexpected course of the reaction of diazomethane with aryl/selenophen-2-yl thioketones of type **1c**, leading to unusual dimers **5** of intermediate thiocarbonyl ylides of type **3c** [22] (Scheme 2). In a competitive reaction, the latter react with the starting thioketone **1c** to give 1,3-dithiolanes of type **6** which are, apparently, also formed via a diradical pathway, leading to the sterically crowded 4,4,5,5-tetrasubstituted isomers exclusively.

In other studies, performed with cycloaliphatic thioketones, e.g. with adamantane-2-thione or 2,2,4,4-tetramethyl-3-thioxocyclobutanone, the growing stability of the corresponding 1,3,4-thiadiazolines obtained in reactions with diazomethane, diazoethane, and 2-diazopropane was reported based on kinetic data [9,17–19]. The same tendency was observed in a series of reactions with aromatic thioketones: for example, the reaction of thiobenzophenone (**1a**) with diazoethane performed at −70 °C led to the formation of a relatively stable 2-methyl-1,3,4-thiadiazoline, which could be identified in the low temperature ^1^H NMR spectrum [20]. To the best of our knowledge, there are no reports on the course of reactions of either diaryl, aryl/hetaryl or dihetaryl thioketones with 2-diazopropane.

Prompted by these observations, we decided to examine reactions of aryl/hetaryl and dihetaryl thioketones **1** with some diazomethane derivatives, such as 2-diazopropane (**7a**), diazoethane (**7b**), and (trimethylsilyl)diazomethane (**7c**), and to compare their outcome with earlier reported reactions with diazomethane [22]. An important question was if in these reactions the corresponding 2-substituted 1,3,4-thiadiazolines of type **2** can be obtained at low temperature and subsequently used as precursors of new thiocarbonyl ylides. The latter may be potentially useful for the [3 + 2]-cycloaddition reactions with diverse dipolarophiles leading to five-membered *S*-heterocycles, such as di- and tetrahydrothiophenes, 1,3-oxathiolanes, 1,3-dithiolanes, etc.

## Results and Discussion

**Scheme 3:**
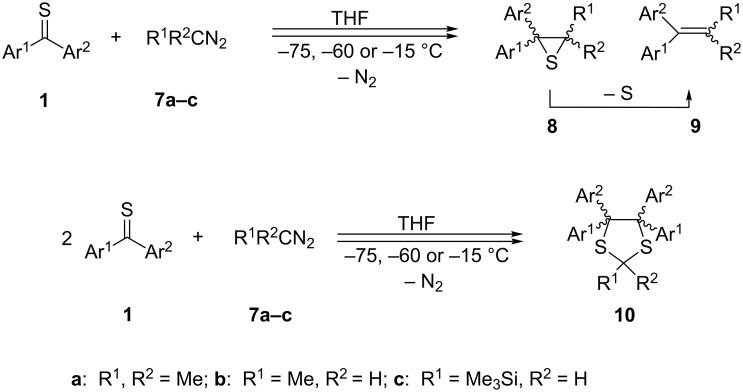
Formation of thiiranes **8** and/or 1,3-dithiolanes **10** in the reaction of aryl/aryl, aryl/hetaryl and dihetaryl thioketones **1** with 2-diazopropane (**7a**), diazoethane (**7b**), and (trimethylsilyl)diazomethane (**7c**) (Table 1).

The first experiment performed with thiobenzophenone (**1a**) and 2-diazopropane (**7a**) at ca. −75 °C in THF led to a change of the blue color of the solution without evolution of N_2_. The addition of the solution of **7a** was continued until the color of the mixture changed to pale red. The mixture was warmed up, and around 0 °C the evolution of N_2_ was observed. The ^1^H NMR analysis of the crude product revealed the presence of only one singlet for two Me groups located at 1.62 ppm. After chromatographic work-up, the only product isolated as colorless crystals was identified as 2,2-dimethyl-3,3-diphenylthiirane (**8a**; Scheme 3, Table 1). The same result was obtained when the reaction was carried out at ca. −60 or −15 °C.

**Table 1:** Reactions of aryl/aryl, aryl/hetaryl, and hetaryl/hetaryl thioketones **1** with 2-diazopropane (**7a**), diazoethane (**7b**), and (trimethylsilyl)diazomethane (**7c**).

Entry	Thioketone **1**	Diazoalkane **7**	*T* [°C]	Product **8**/**9**	Yields **8**/**9** [%]^a^	Product **10** (and/or **4**)	Yield **10** (or **4**) [%]^a^

1	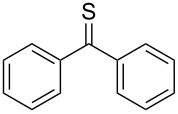 **1a**	**7a** R^1^ = R^2^ = Me	−15 −60 −75	**8a**/**9a**	86 80 (76^b^) 75		– – –
2	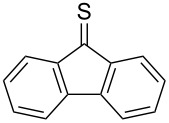 **1b**	**7a**	−15 −60 −75	**8b/9b**	61 (41^b^) 85 76	**10b**	14 (12^d^) – –
3	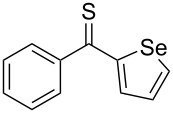 **1c**	**7a**	−15 −60 −75	**8c/9c**	42 74 (57^b^) 63	**10c**	61 13 (11^d,e^) 18
4	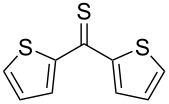 **1d**	**7a**	−15 −60 −75	**8d/9d**	56 87 (75^b^ + 2^c^) 97	**10d**	58 36 (32^d^) 7
5	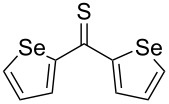 **1e**	**7a**	−15 −60 −75	**8e/9e**	50 84 (61^c^) 68	**10e**	49 20 (17^d^) 27
6	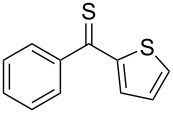 **1f**	**7a**	−15 −60 −75	**8f/9f**	40 (17^b^ + 7^c^) 85 96	**10f**	67 (63^d,e^) 38 –
7	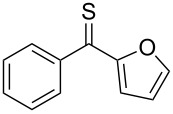 **1g**	**7a**	−15 −60 −75	**8g/9g**	38 51 (40^c^) 69	**10g**	70 31 (28^d,e^) 36
8	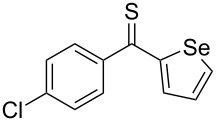 **1h**	**7a**	−15 −60 −75	**8h/9h**	36 88 (65^c^) 72	**10h**	68 21 (17^d,e^) 21
9	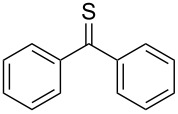 **1a**	**7b** R^1^ = Me, R^2^ = H	rt −75	**8i/9i**	traces (see, ref. [23]) 93^b^ + 3^c^ (see, ref. [20])	**10i**	87 (see, ref. [23]) –
10	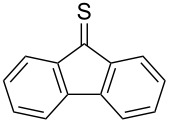 **1b**	**7b**	−15 −60 −75	**8j/9j**	3 9 (6^c^) 14	**10j**	82 73 (61^d^) 57
11	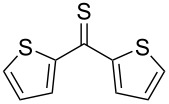 **1d**	**7b**	−15 −60 −75	**8k/9k**	39 32 (25^c^) 69	**10k**	85 81 (72^d^) 43
12	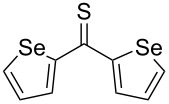 **1e**	**7b**	−15 −60 −75	**8l/9l**	11 5 (3^c^) 12	**10l**	76 86 (79^d^) 87
13	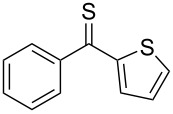 **1f**	**7b**	−15 −60 −75	**8m/9m**	6 59 (48^c^) 62	**10m**	79 35 (27^d,e^) 28
14	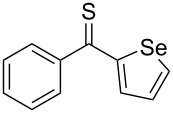 **1c**	**7b**	−15 −60 −75	**8n/9n**	20 83 (71^c^) 65	**10n**	75 36 (28^d,e^) 30
15	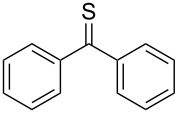 **1a**	**7c** R^1^ = Me_3_Si, R^2^ = H	−75		–	**10o** and **4c**	36 (33^d^) 39 (22^f^)
16	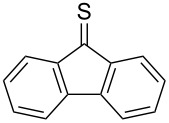 **1b**	**7c**	−75		–	**4d**	53^f^ see ref. [26]
17	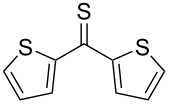 **1d**	**7c**	−75		–	**10r**	95 (87^d^)
18	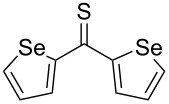 **1e**	**7c**	−75		–	**10s**	85 (76^d^)

^a^Yields determined by ^1^H NMR with a weighed amount of 1,1,2,2-tetrachloroethane as a standard. ^b^Yield of isolated product **8**. ^c^Yield of isolated product **9**. ^d^Yield of isolated product **10**. ^e^Isolated as mixtures of isomeric products. ^f^Yield of isolated product **4**.

Similar experiments were performed with thiofluorenone (**1b**) and **7a**. Whereas at −75 °C and −60 °C the corresponding thiirane **8b** and ethene **9b**, formed via spontaneous desulfurization of **8b**, were obtained as the sole products, in the experiment carried out at –15 ºC, the sterically crowded 1,3-dithiolane **10b** was also detected as a minor product (14% yield, Table 1). In the latter case, thiirane **8b** and ethene **9b** were found in 24 and 37% yield, respectively. According to the literature, reaction of thiobenzophenone (**1a**) with diazoethane (**7b**) performed at –75 °C led to 5-methyl-2,2-diphenyl-1,3,4-thiadiazoline as the exclusive product [20]. After warming the reaction mixture to room temperature, the only products observed in the mixture were 2,2-diphenyl-3-methylthiirane (**8i**) and 1,1-diphenylpropene (**9i**) as the product of its desulfurization. On the other hand, the experiment with **1a** and **7b** performed at room temperature led to the 1,3-dithiolane **10i** as the major product (87%) accompanied by small amounts of thiirane **8i** and ethene **9i** [23].

A different result was observed in the reaction of **7a** with di(thiophen-2-yl) thioketone (**1d**). In that case, the reaction at −75 °C afforded also the expected thiirane **8d** as the major product, accompanied by small amounts of the corresponding alkene **9d**. However, in that case 4,4,5,5-tetrasubstituted 1,3-dithiolane **10d** was also observed (7%). The amount of the latter product increased to 36% when the reaction was performed at −60 °C and to 58% at −15 °C. The corresponding experiments with **1c** and **7b** led to increased amounts of 1,3-dithiolane **10k**, established as 43% at −75 °C, 81% at −60 °C, and 85% at −15 °C (Table 1). The same tendency, i.e., an increasing amount of 1,3-dithiolanes **10** in the case of the less-substituted diazoethane (**7b**) and at higher temperature, was observed for the reactions with thiofluorenone (**1b**), di(selenophen-2-yl)-, (phenyl)(thiophen-2-yl)-, (phenyl)(selenophen-2-yl)-, and other aryl hetaryl thioketones **1** (Table 1). As mentioned before, in some cases tri- and tetrasubstituted thiiranes partially underwent spontaneous extrusion of sulfur to form the corresponding tri- or tetrasubstituted ethene derivatives **9**. In these cases, complete desulfurization was achieved by treatment of the reaction mixture with tris(diethylamino)phosphine, and the respective ethenes **9** were isolated as the final products.

It is worth mentioning that in the case of the non-symmetrical aryl/hetaryl thioketones **1c, 1f**–**h** and 2-diazopropane (**7a**), the formed 1,3-dithiolanes **10** were isolated as mixtures of *cis*- and *trans*-isomers. In the ^1^H NMR spectra, they could be identified based on the presence of two and one signal, respectively, for the Me_2_C(2) group. The mixtures of 1,3-dithiolanes **10** obtained from the reactions of diazoethane (**7b**) with thioketones **1c**,**f** consisted of only two instead of the expected three diastereoisomers. In all cases they belong to the same group of regioisomers. The regioselective formation of products **10** was proved by ^13^C NMR spectroscopy: the signals attributed to C(2) were found in narrow regions at 54.9–59.4 ppm for **10c**, **10f**–**h** and 43.1–45.6 ppm for **10m**,**n**, respectively. In addition, the structure of the sterically crowded 4,4,5,5-tetrasubstituted aryl/hetaryl 1,3-dithiolanes **10** could be confirmed by the presence of only two signals for the three C(sp^3^)-atoms of the heterocycle, whereas in the isomeric 2,2,4,4-tetraaryl/hetaryl 1,3-dithiolanes, three signals for these atoms are expected (cf. [19]).

Trimethylsilyldiazomethane (**7c**) is widely applied as a practical and useful synthetic equivalent of the hazardous diazomethane [24–25]. In our earlier publications, its reactions with thiofluorenone (**1b**) and *S*-methyl (phosphonyl)dithioformate, leading to the expected 1,3,4-thiadiazoline derivatives, which are stable at −60 °C, were reported [26–27]. At higher temperature, in both reactions, dimers of the intermediate thiocarbonyl *S*-(trimethylsilyl)methanides were formed in the absence of a dipolarophile after evolution of N_2_. An analogous test experiment with **7c** and thiobenzophenone (**1a**) was carried out in the course of the present study at −75 °C, and in this case slow decolorization of the reaction solution was observed. In contrast to the experiment with **1b**, complete decolorization of the blue reaction solution was observed before the addition of the total, equimolar amount of **7c**. After warming up and typical work-up procedure, the corresponding 1,3-dithiolane **10o** and 1,4-dithiane **4c** in a ratio of 2:1 were found as products identified in the ^1^H NMR spectrum of the crude reaction mixture. Thus, in this reaction dimerization of the intermediate thiocarbonyl ylide and its reaction with another molecule of **1a** were competitive pathways. Finally, the reactions of **7c** with symmetrically substituted dihetaryl thioketones **1d** and **1e** were performed at −75 °C, and in both cases, the sterically crowded 4,4,5,5-tetrahetaryl-1,3-dithiolanes **10r** and **10s**, respectively, were obtained as sole products.

**Scheme 4:**
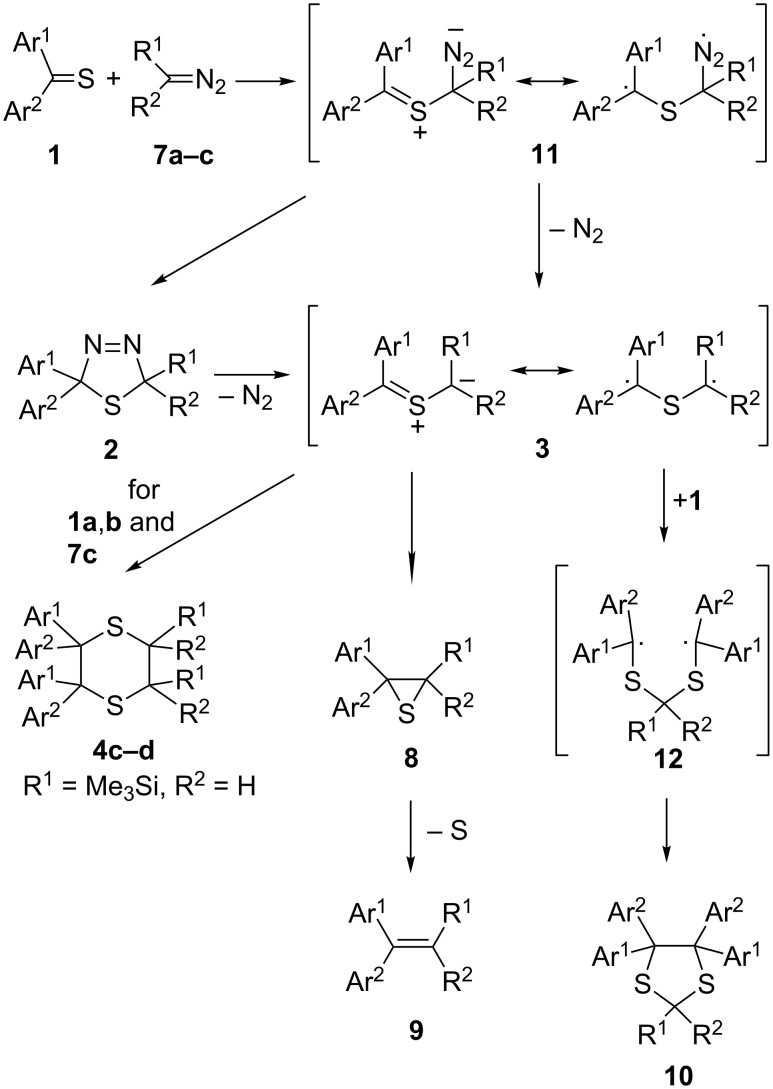
Proposed competitive mechanisms in the reactions of aryl/hetaryl and dihetaryl thioketones **1** with 2-diazopropane (**7a**), diazoethane (**7b**), and (trimethysilyl)diazomethane (**7c**).

The obtained results can be explained by the assumption that in the case of hetaryl thioketones **1** stepwise mechanisms via diradical intermediates govern the formation of the isolated 1,3-dithiolanes **10** (Scheme 3). Based on earlier studies, the stability of 1,3,4-thiadiazolines **2**, which are considered as potential precursors of thiocarbonyl ylides **3**, should be increased by the introduction of Me or Me_3_Si groups. Upon this assumption, all reactions performed at −75 °C should lead to the corresponding cycloadducts **2** with complete conversion of the starting thioketones **1**. Only after warming up above −45/40 °C compounds **2** are expected to decompose yielding the reactive thiocarbonyl ylide **3**. Under these conditions, the latter intermediates can undergo either 1,3-dipolar electrocyclization to give thiiranes **8** or dimerization leading to 1,4-dithianes **4** [20,26]. This reaction course resulting in the exclusive formation of thiiranes **8** was observed in the case of thiobenzophenone (**1a**) and thiofluorenone (**1b**) with 2-diazopropane (**7a**). However, the reaction of **1b** with **7a** carried out at −15 °C yielded also a small amount of 1,3-dithiolane **10b**. This result can be interpreted by the partial decomposition of **2b** in the presence of the non-converted thioketone **1b**. The replacement of aromatic thioketones **1a**,**b** by di(thiophen-2-yl) thioketone (**1d**) in the reaction with **7a** resulted in the formation of comparable amounts of thiirane **8d** and 1,3-dithiolane **10d**, whereas at −75 °C **8d** is again the major product. It is worth mentioning that the analogous experiment carried out at –60 ºC, i.e., below the expected decomposition temperature of 1,3,4-thiadiazoline **2d**, led to substantial increase of the amount of **10d**. A similar tendency was observed in other studied cases with hetaryl thioketones and **7a**. The replacement of the latter by diazoethane (**7b**), leading to the less stable 1,3,4-thiadiazolines **2**, resulted in a general increase of the corresponding 1,3-dithiolanes **10**, which were formed in substantial amounts, even in experiments performed at −75 °C. However, the most striking results were observed in reactions performed with dihetaryl thioketones **1d** and **1e** with trimethylsilyldiazomethane (**7c**). In both experiments, the only products formed were the corresponding, sterically crowded 4,4,5,5,-tetrahetarylsubstituted 1,3-dithiolanes **10r** and **10s**. These results clearly demonstrate that **7c**, similarly to diazomethane [22], reacts smoothly with dihetaryl thioketones **1** with no formation of the expected 1,3,4-thiadiazolines **2** and after release of N_2_ even at low temperature, the intermediate diradicals of type **3** attack the parent thioketones **1** yielding 1,3-dithiolanes **10** via stabilized 1,5-diradicals **12**. In both cases no tendency for the formation of dimers of the intermediate ‘thiocarbonyl ylide’ was observed (Scheme 4).

Based on these results, the reaction mechanism can be proposed as formulated in Scheme 4. It seems likely that the first step comprising the reaction of a hetaryl thioketone **1** with **7a, 7b**, and **7c** is not a concerted process, but the diradical or zwitterionic intermediate of type **11** is formed and, depending on the number and type of stabilizing substituents and the reaction temperature, they undergo two competitive reactions: elimination of N_2_ leading to thiocarbonyl ylide **3** or 1,5-ring closure to give 1,3,4-thiadiazoline **2**. When the rapid elimination of N_2_ occurs in the presence of the non-converted thioketone **1**, the stabilized 1,5-diradical **12** is formed as precursor of the sterically crowded 1,3-dithiolane **10**. The alternatively formed 1,3,4-thiadiazolines **2a** and **2b** are expected to be stable at –75 ºC and eliminate N_2_ only at enhanced temperature generating thiocarbonyl ylide **3**. As in that case the starting diaryl (or dihetaryl) thioketone **1** is completely consumed, the 1,3-dithiolane **10** cannot be formed. The results obtained with **7c** demonstrate that its reaction with dihetaryl thioketones **1** occurs without formation of 1,3,4-thiadiazolines as intermediate [3 + 2]-cycloadducts. Elimination of N_2_ from the initially formed diradical of type **11** leads immediately to the new diradical species **3**, which adds regioselectively to the C=S group of the non-converted thioketone **1**.

This interpretation is consistent with the course of the reactions of aryl/hetaryl thioketones **1** with diazomethane, in which the formation of sterically crowded 1,3-dithiolanes side-by-side with 12-membered dimers of the thiocarbonyl ylide was observed [22]. In addition, it corresponds to the diradical mechanism postulated for the formal [3 + 2]-cycloaddition of aryl/hetaryl thioketones **1** with the in situ generated thiocarbonyl *S*-methanides [28]. The missing formation of dimers from thiocarbonyl ylides **3** derived from hetaryl thioketones can be explained by steric hindrance resulting from the presence of the Me or Me_3_Si groups in the *S*-methanide moiety. However, electronic effects resulting from the diradical nature of the intermediate thiocarbonyl ylides, can also play certain role. The same effect was observed in the case of diaryldiazomethanes used in reactions with aryl/hetaryl thioketones [29–30].

## Experimental

**General information**: Melting points were determined in capillaries using a MEL-TEMP II apparatus (Aldrich) and are uncorrected. IR spectra were recorded with a FTIR NEXUS spectrophotometer as KBr pellets or as film; absorptions (ν) in cm^−1^. ^1^H and ^13^C NMR spectra were measured on a Bruker Avance III (^1^H at 600 and ^13^C at 150 MHz) instrument in CDCl_3_; chemical shifts (δ) are given in ppm, solvent signals as reference, coupling constants (*J*) in Hz. The multiplicity of the ^13^C signals was deduced from DEPT, supported by ^1^H,^13^C HMQC spectra. ^1^H NMR data are presented as follows: chemical shift, multiplicity (br = broad, s = singlet, d = doublet, t = triplet, q = quartet, m = multiplet), coupling constant, integration. The mass spectra were recorded on a Finnigan MAT-95 (ESI). Elemental analyses were performed in the Microanalytical Laboratory of the Chemistry Faculty of the University of Łódź. Applied reagents such as 2-diazopropane (**7a**) and diazoethane (**7b**) were prepared by known methods according to the literature protocols [31–32]. Thiobenzophenone (**1a**), fluorene-9-thione (thiofluorenone, **1b**), symmetrical dihetaryl thioketones **1d**,**e**, and nonsymmetrical aryl/hetaryl thioketones **1c**, **1f–h** were obtained from the corresponding ketones using the known procedures [33]. Other reagents used in the present study were commercially available.

**Reaction of thioketones 1a–h with 2-diazopropane (7a) and diazoethane (7b) – General procedure:** Corresponding thioketones **1a–h** (1 mmol) were dissolved in freshly distilled THF (2.5 mL) and the solution was cooled to the corresponding temperature (−15, −60, −75 °C; acetone/dry ice). Then, the mixture was treated with small portions of ethereal 2-diazopropane (**7a**) or diazoethane (**7b**) solution, until the intense color of the thioketone vanished. Then, the mixture was allowed to warm slowly to rt (ca. 2–4 h). After removal of the solvent under vacuum, the residue was subjected to ^1^H NMR analysis in CDCl_3_ solution with a weighed amount of 1,1,2,2-tetrachloroethane as a standard. Crude products were purified by CC (CHCl_3_/hexane 2:8).

In crude mixtures obtained from 2-diazopropane (**7a**) and thioketones **1c,e,g,h** and from diazoethane (**7b**) and thioketones **1b–f**, the presence of thiirane and the corresponding ethylene was evidenced based on the ^1^H NMR spectra. In these cases, no isolation of the thiirane was performed; after addition of tris(dimethylamino)phosphine desulfurization leading to the ethane derivative was carried out.

**Reaction of thioketones 1d–e with (trimethylsilyl)diazomethane (7c) – General procedure:** The corresponding thioketones **1d**–**e** (1 mmol) were dissolved in freshly distilled THF (2.5 mL) and the solution was cooled to −75 °C (acetone/dry ice). Then, the mixture was treated with small portions of an ethereal solution of **7c** (1 mmol). The mixture was kept in a cold bath, and the intense color of the thioketone vanished after 15 min. Subsequently, the mixture was allowed to slowly warm to rt (ca. 2–4 h). After removal of the solvent under vacuum, the residue was subjected to the ^1^H NMR analysis in CDCl_3_ solution with a weighed amount of 1,1,2,2-tetrachloroethane as an internal standard. Crude products were purified by CC (CHCl_3_/hexane 2:8).

**2,2-Dimethyl-3,3-diphenylthiirane** (**8a**)**:** Yield: 182 mg (76%). White crystals; mp 66–67 °C (chromatographic purification); IR (KBr) ν: 2987 (w), 2921 (w), 1596 (w), 1490 (m), 1443 (m), 773 (m), 747 (m), 705 (s), 691 (m) cm^−1^; ^1^H NMR (600 MHz, CDCl_3_) δ 7.60–7.58 (m, 4H, H_arom_), 7.31–7.29 (m, 4H, H_arom_), 7.23–7.21 (m, 2H, H_arom_), 1.62 (s, 6H, CH_3_) ppm; ^13^C NMR (150 MHz, CDCl_3_) δ 142.4 (for 2 C_arom_), 129.4, 127.9, 126.8 (for 10 CH_arom_), 67.8, 52.9 (C-2, C-3), 27.9 (2 CH_3_) ppm; ESIMS *m/z* (%): 241 (100, [M + H]^+^); anal. calcd for C_16_H_16_S (240.36): C, 79.95; H, 6.71; S, 13.34; found: C, 79.69; H, 6.50; S, 13.40.

**1,1-Di(thiophen-2-yl)-2-methylpropene** (**9d**)**:** Yield: 4 mg (2%). Yellow oil; IR (film) ν: 2927 (w), 2908 (w), 2850 (w), 1436 (m), 1369 (m), 1227 (m), 1016 (w), 827 (m), 693 (s) cm^−1^; ^1^H NMR (600 MHz, CDCl_3_) δ 7.29 (d, *J* = 5.4 Hz, 2H, H_arom_), 7.04–7.02 (m, 2H, H_arom_), 6.91 (d, *J* = 3.6 Hz, 2H, H_arom_), 2.03 (s, 6H, CH_3_) ppm; ^13^C NMR (150 MHz, CDCl_3_) δ 144.9, 137.1 (C_arom_, C_arom_-*C*=), 126.6, 126.4, 124.9 (for 6 CH_arom_), 122.9 (C=*C*(CH_3_)_2_), 23.3 (2 CH_3_) ppm; anal. calcd for C_12_H_12_S_2_ (220.35): C, 65.41; H, 5.49; S, 29.10; found: C, 65.31; H, 5.76; S, 28.81.

**2,2-Dimethyl-4,5-diphenyl-4,5-di(thiophen-2-yl)-1,3-dithiolane** (**10f**)**:** Isolated as a mixture of *cis*-, *trans*-isomers (crude product ratio 65:35). Yield: 142 mg (63%). White crystals; mp 153–154 °C (MeOH/CHCl_3_); IR (KBr) ν: 3062 (w), 2919 (w), 1595 (w), 1490 (m), 1443 (m), 1231 (m), 1156 (m), 854 (w), 697 (s) cm^−1^; ^1^H NMR (600 MHz, CDCl_3_) δ 7.59–6.68 (m, 32H, H_arom_), 1.86 (s, 3H, CH_3_-*cis*), 1.70 (s, 6H, CH_3_-*trans*), 1.39 (s, 3H, CH_3_-*cis*) ppm; ^13^C NMR (150 MHz, CDCl_3_) δ 151.2, 149.8, 144.0, 142.9 (for 8 C_arom_), 131.3, 131.1, 130.5, 127.5, 127.1, 127.0, 126.6, 126.1, 125.4, 125.2, 125.0 (for 32 CH_arom_), 78.8, 78.4 (C-4 + C-5, for *cis* and *trans*), 55.4, 55.3 (C-2, *cis* and *trans*) 33.1 (CH_3_-*cis*), 32.9 (for 2 CH_3_-*trans*), 32.5 (CH_3_-*cis*) ppm; anal. calcd for C_25_H_22_S_4_ (450.70): C, 66.62; H, 4.92; S, 28.46; found: C, 66.46; H, 4.91; S, 28.33.

**1,1-Di(thiophen-2-yl)-propene** (**9k**)**:** Yield: 52 mg (25%) – After desulfurization of tiirane **8k**. Yellow oil; IR (film) ν: 3104 (w), 3071 (w), 2930 (w), 2908 (w), 2850 (w), 1438 (m), 1362 (w), 1249 (m), 1223 (m), 1036 (w), 850 (s), 836 (s), 818 (s), 695 (s) cm^−1^; ^1^H NMR (600 MHz, CDCl_3_) δ 7.23–7.22 (m, 1H, H_arom_), 7.01–7.00 (m, 1H, H_arom_), 6.96–6.94 (m, 1H, H_arom_), 6.90–6.89 (m, 1H, H_arom_), 6.80–6.79 (m, 1H, H_arom_), 6.71–6.70 (m, 1H, H_arom_), 6.18 (q, *J* = 7.2 Hz, 1H, =CH), 1.74 (d, *J* = 7.2 Hz, 3H, CH_3_) ppm; ^13^C NMR (150 MHz, CDCl_3_) δ 129.4, 139.8, 146.8 (2 C_arom_, C_arom_, *C*=), 127.6, 127.0, 126.6, 126.3, 125.5, 124.8, 123.4 (6 CH_arom_, =*C*H), 15.6 (CH_3_) ppm; anal. calcd for C_11_H_10_S_2_ (206.33): C, 64.04; H, 4.89; S, 31.08; found: C, 63.83; H, 4.98; S, 31.14.

**2-Trimethylsilyl-4,4,5,5-tetra(thiophen-2-yl)-1,3-dithiolane** (**10r**)**:** Yield: 220 mg (87%). Yellow crystals: mp 152–154 °C (hexane/CH_2_Cl_2_); IR (KBr) ν: 2951 (w), 1618 (w), 1424 (m), 1250 (s), 1232 (m), 1122 (m), 1077 (m), 1050 (m), 842 (s), 752 (s), 699 (s), 632 (m) cm^−1^; ^1^H NMR (600 MHz, CDCl_3_) δ 7.23–7.22 (m, 2H, H_arom_), 7.20–7.19 (m, 2H, H_arom_), 6.96–6.95 (m, 2H, H_arom_), 6.89–6.87 (m, 2H, H_arom_), 6.83–6.81 (m, 4H, H_arom_), 3.89 (s, 1H, ((CH_3_)_3_Si)*H*C), 0.34 (s, 9H, (C*H*_3_)_3_Si) ppm; ^13^C NMR (150 MHz, CDCl_3_) δ 148.1, 146.1 (for 4 C_arom_), 130.2, 129.4, 127.0, 125.6, 125.5, 125.4 (for 12 CH_arom_), 74.9 (C-4, C-5), 36.5 (C-2), −1.53 ((*C*H_3_)_3_Si) ppm; anal. cald for C_22_H_22_S_6_Si (506.89): C, 52.13; H, 4.37; S, 37.96; found: C, 52.44; H, 4.55; S,37.71.

## Supporting Information

File 1 Experimental data for selected compounds **8**–**10**, and the original ^1^H and ^13^C NMR spectra for all products.
