# Supplementary material for: Diradical reaction mechanisms in [3 + 2]-cycloadditions of hetaryl thioketones with alkyl- or trimethylsilyl-substituted diazomethanes
Source: Beilstein J Org Chem. 2016 Apr 14;12:716–24. doi: 10.3762/bjoc.12.71 (PMC4901872; doi:10.3762/bjoc.12.71)
Supplement: File 1 — Experimental data for selected compounds 8–10, and the original 1H and 13C NMR spectra for all products. [file Beilstein_J_Org_Chem-12-716-s001.pdf]

## Supporting Information

## for

# Diradical reaction mechanisms in [3 + 2]-cycloadditions of hetaryl thioketones with alkyl- or trimethylsilyl-substituted diazomethanes

Grzegorz Mlostoń[*1], Paulina Pipiak[1] and Heinz Heimgartner[2]

Address: [1]Department of Organic and Applied Chemistry, University of Łódź, Tamka 12, PL 91-403 Łódź, Poland and [2]Department of Chemistry, University of Zürich, Winterthurerstrasse 190, CH-8057 Zürich, Switzerland

Email: Grzegorz Mlostoń - gmloston@uni.lodz.pl

*Corresponding author

## Experimental data for compounds 8, 9, 10 and the original $^{1}$H and $^{13}$C NMR spectra

1. Experimental data for compound **8**, **9** and **10**.

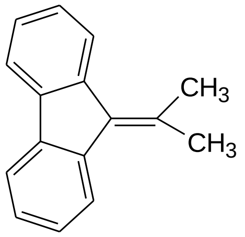


*9-Isopropylidenefluorene* (**9b**) [S1]: Yield: 85 mg (41%). White crystals; m.p. 114 – 115 °C (chromatographic purification; lit. m.p. 114 °C [S1]). $^{1}$H NMR (600 MHz, $CDCl_3$): δ = 7.78–7.89 (m, 4 $H_{arom}$), 7.30–7.35 (m, 4 $H_{arom}$), 2.58 (s, 2 $CH_3$) ppm. $C_{16}H_{14}$(206.28): calcd. C 93.16 H 6.84; found C 93.13 H 6.98.

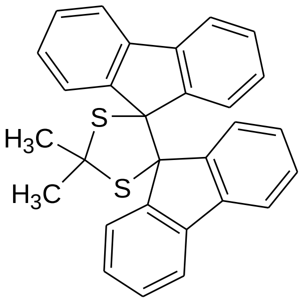

*2',2'-Dimethyldispiro[9*H*-fluorene-9,4'-(1,3)dithiolane-5'9''-[9*H*]fluorene]* (**10b**): Yield: 27 mg (12%). White crystals; m.p. 258 – 260 °C (MeOH/$CHCl_3$). IR (KBr): ν = 2918 (w), 1445 (m), 1279 (w), 1149 (m), 1108 (w), 1033 (w), 760 (m), 744 (s) $cm^{-1}$. $^{1}H$ NMR (600 MHz, $CDCl_3$): δ = 7.97 (br. s, 4 $H_{arom}$), 7.42 (t, *J* = 7.8Hz, 4 $H_{arom}$), 7.25 (t, *J* = 7.2Hz, 4 $H_{arom}$), 7.12 (t, *J* = 7.2Hz, 4 $H_{arom}$), 2.37 (s, 2 $CH_3$) ppm. $^{13}C$ NMR (150 MHz, $CDCl_3$): δ = 140.1 (for 8 $C_{arom}$), 128.5, 127.8, 126.3, 119.3 (for 16 $CH_{arom}$), 59.4 (C-2), 78.4 (C-4, C-5), 35.2 (2 $CH_3$) ppm. $C_{29}H_{22}S_2$ (434.61): calcd. C 80.14, 5.10, 14.76; found C 80.0, 5.27, 14.95.

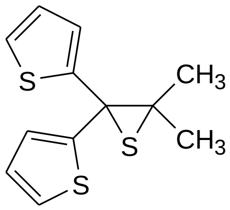

*2,2-Dimethyl-3,3-di(thiophen-2-yl)thiirane* (**8d**): Yield: 190 mg (75%). Yellow crystals; m.p. 50 – 51 °C (chromatographic purification). IR (KBr): ν = 3099 (w), 2955 (w), 2917 (w), 1429 (m), 1367 (m), 1244 (m), 1231 (m), 1085 (m), 1077 (m), 849 (m), 833 (s), 783 (m), 705 (s), 560 (m) $cm^{-1}$. $^{1}H$ NMR (600 MHz, $CDCl_3$): δ = 7.07 (dd, *J* = 1.2 Hz, *J* = 4.8 Hz, 2 $H_{arom}$), 7.00 (dd, *J* = 1.2 Hz, *J* = 3.6 Hz, 2 $H_{arom}$), 6.80–6.81 (m, 2 $H_{arom}$), 1.64 (s, 2 $CH_3$) ppm. $^{13}C$ NMR (150 MHz, $CDCl_3$): δ = 146.2 (for 2 $C_{arom}$), 127.4, 126.5, 125.2 (for 6 $CH_{arom}$), 56.0, 54.8 (C-2, C-3), 26.7 (2 $CH_3$) ppm. MS (ESI): m/z (%) = 253 (100, $[M+H]^+$). $C_{12}H_{12}S_3$ (252.42): calcd. C 57.10, H 4.79, S 38.11; found C 56.91, H 4.80, S 38.12.

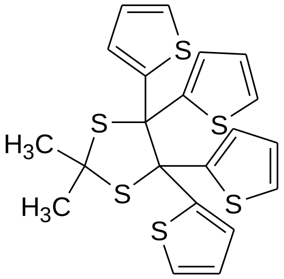

*2,2-Dimethyl-4,4,5,5-tetra(thiophen-2-yl)-1,3-dithiolane* (**10d**): Yield: 74 mg (32%). White crystals; m.p. 150 – 151 °C (MeOH/$CHCl_3$). IR (KBr): ν = 3101 (w), 3085 (w), 2916 (w), 1423 (m), 1234 (m), 1226 (m), 1156 (m), 1106 (m), 1048 (m), 856 (m), 758 (m), 707 (s) $cm^{-1}$. $^1H$ NMR (600 MHz, $CDCl_3$): δ = 7.24 (dd, *J* = 1.2 Hz, *J* = 5.4 Hz, 4 $H_{arom}$), 7.05 (dd, *J* = 1.2 Hz, *J* = 3.6 Hz, 4 $H_{arom}$), 6.86–6.87 (m, 4 $H_{arom}$),1.76 (s, 2 $CH_3$) ppm. $^{13}C$ NMR (150 MHz, $CDCl_3$): δ = 147.7 (for 4 $C_{arom}$), 130.3, 126.3, 125.3 (for 12 $CH_{arom}$), 76.1 (C-4, C-5), 57.5 (C-2), 33.1 (2 $CH_3$) ppm. MS (ESI): m/z (%) = 485 (100, $[M+Na]^+$). $C_{21}H_{18}S_6$ (462.76): calcd. C 54.50, H 3.92, S 41.57; found C 54.44, H 3.76, S 41.82.

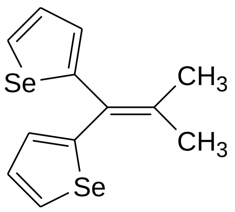


*1,1-Di(selenophen-2-yl)-2-methylpropene* (**9e**): Yield: 192 mg (61%) – After desulfurization of tiirane **8d**. Colorles oil that crystallized in the refrigerator. IR (film): ν = 3054 (m), 2926 (m), 2905 (m), 2850 (w), 1448 (m), 1368 (w), 1267 (w), 1225 (m), 1168 (w), 1078 (w), 1000 (w), 829 (w), 783 (w), 681 (s) $cm^{-1}$. $^1H$ NMR (600 MHz, $CDCl_3$): δ = 7.98 (d, *J* = 6.0 Hz, 2 $H_{arom}$), 7.22–7.23 (m, 2 $H_{arom}$), 7.05 (d, *J* = 3.6 Hz, 2 $H_{arom}$), 1.98 (s, 2 $CH_3$) ppm. $^{13}C$ NMR (150 MHz, $CDCl_3$): δ = 151.8, 135.6 ($C_{arom}$, $C_{arom}$-*C*=),), 130.7, 129.0, 128.6 (for 6 $CH_{arom}$), 127.3 (C=*C*$(CH_3)_2$) 23.5 (2 $CH_3$) ppm. MS (ESI): m/z (%) = 315 (100, $[M+H]^+$). $C_{12}H_{12}Se_2$ (314.14): calcd. C 45.88, H 3.85; found C 46.14, H 3.70.

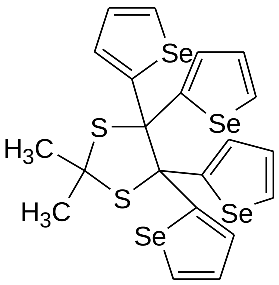


*2,2-Dimethyl-4,4,5,5-tetra(selenophen-2-yl)-1,3-dithiolane* (**10e**): Yield: 56 mg (17%). White crystals; m.p. 123 – 124 °C (MeOH/$CHCl_3$). IR (KBr): ν = 2952 (w), 2917 (w), 1434 (m), 1362 (w), 1230 (s), 1154 (m), 1103 (m), 1025 (m), 797 (m), 688 (s) $cm^{-1}$. $^1H$ NMR (600 MHz, $CDCl_3$): δ = 7.96 (dd, *J* = 1.2 Hz, *J* = 6.0 Hz, 4 $H_{arom}$), 7.39 (dd, *J* = 1.2 Hz, *J* = 4.2 Hz, 4 $H_{arom}$), 7.11–7.13 (m, 4 $H_{arom}$), 1.77 (s, 2 $CH_3$) ppm. $^{13}C$ NMR (150 MHz, $CDCl_3$): δ = 154.8 (for 4 $C_{arom}$), 133.3, 133.2, 127.9 (for 12 $CH_{arom}$), 79.8

(C-4, C-5), 58.1 (C-2), 33.2 (2 $CH_3$) ppm. $C_{21}H_{18}S_2Se_4$ (650.34): calcd. C 38.78, H 2.79, S 9.86; found C 38.56, H 2.93, S 9.78.

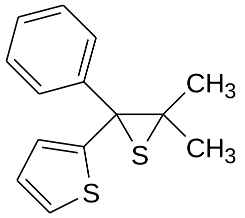


*2,2-Dimethyl-3-phenyl-3-(thiophen-2-yl)thiirane* (**8f**): Yield: 42 mg (17%). White crystals; m.p. 44 – 45 °C (chromatographic purification). IR (KBr): $\nu$ = 2997 (w), 2919 (w), 1491 (w), 1443 (m), 1369 (w), 1228 (m), 1075 (m), 753 (m), 702 (s) $cm^{-1}$. $^1H$ NMR (600 MHz, $CDCl_3$): $\delta$ = 7.55–7.56 (m, 2 $H_{arom}$), 7.32–7.35 (m, 2 $H_{arom}$), 7.27–7.28 (m, 1 $H_{arom}$), 7.14 (*dd*, *J* = 1.2 Hz, *J* = 5.4 Hz, 1 $H_{arom}$), 7.02 (*dd*, *J* = 1.2 Hz, *J* = 3.6 Hz, 1 $H_{arom}$), 6.87 (*dd*, *J* = 3.6 Hz, *J* = 4.8 Hz, 1 $H_{arom}$), 1.68 (s, $CH_3$), 1.53 (s, $CH_3$) ppm. $^{13}C$ NMR (150 MHz, $CDCl_3$): $\delta$ = 147.3, 141.7 (2 $C_{arom}$), 129.5, 127.9, 127.3, 127.2, 126.5, 125.3 (for 8 $CH_{arom}$), 61.5, 54.4, (C-2, C-3), 26.3 ($CH_3$), 28.3 ($CH_3$) ppm. MS (ESI): m/z (%) = 247 (100, $[M+H]^+$). $C_{14}H_{14}S_2$ (246.39): calcd. C 68.25, H 5.73, S 26.03; found C 68.37, H 5.56, S 26.23.

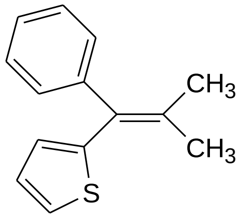


*1-Phenyl-1-(thiophen-2-yl)-2-methylpropene* (**9f**) [S2]: Yield 15 mg (7%). Pale yellow oil (lit. [S2]: colorless liquid). $^1H$ NMR (600 MHz, $CDCl_3$): $\delta$ = 7.21–7.36 (m, 6 $H_{arom}$), 6.97–6.98 (m, 1 $H_{arom}$), 6.79–6.80 (m, 1 $H_{arom}$), 2.06 (s, $CH_3$), 1.79 (s, $CH_3$) ppm.

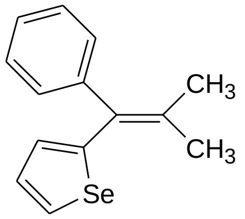


*1-Phenyl-1-(selenophen-2-yl)-2-methylpropene* (**9c**): Yield 148 mg (57%) – After desulfurization of tiirane **8f**. Yellow oil. IR (film): $\nu$ = 3055 (m), 2907 (m), 2853 (m), 1598 (w), 1491 (m), 1442 (s), 1369 (m), 1218 (m), 1019 (m), 757 (m), 700 (s), 685 (s) $cm^{-1}$. $^1H$ NMR (600 MHz, $CDCl_3$): $\delta$ = 7.91 (dd, *J* = 1.2 Hz, *J* = 6.0 Hz, 1 $H_{arom}$), 7.21–7.36 (m, 6 $H_{arom}$), 6.97 (dd, *J* = 1.2 Hz, *J* = 3.6 Hz, 1 $H_{arom}$), 2.06 (s, $CH_3$), 1.77 (s, $CH_3$) ppm. $^{13}C$ NMR (150 MHz, $CDCl_3$): $\delta$ = 151.9, 143.7, 132.7, 132.5 (2 $C_{arom}$,

$C_{arom}$-*C*=*C*($CH_3$)$_2$), 130.3, 129.4, 129.0, 128.6, 128.0, 126.5 (for 8 $CH_{arom}$), 23.5 ($CH_3$), 22.8 ($CH_3$) ppm. MS (ESI): m/z (%) = 263 (100, [*M*+2H]$^+$), 261 (40, [*M*]$^+$). $C_{14}H_{14}Se$ (261.22): calcd. C 64.37, H 5.40; found C 64.41, H 5.61.

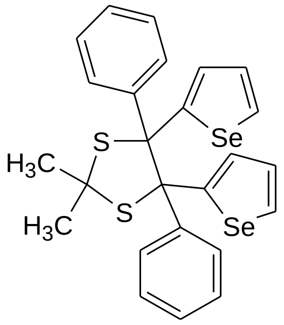


*2,2-Dimethyl-4,5-diphenyl-4,5-di(selenophen-2-yl)-1,3-dithiolane* (**10c**): Isolated as a mixture of *cis*,*trans*-isomers (crude product ratio 72 : 28). Yield: 30 mg (11%). White crystals; m.p. 137 – 138 °C (MeOH/$CHCl_3$). IR (KBr): ν = 3057 (w), 2922 (w), 2853 (w), 1596 (w), 1490 (m), 1444 (s), 1364 (m), 1232 (m), 1181 (m), 1155 (m), 1033 (m), 931 (m), 692 (s) $cm^{-1}$.$^1$H NMR (600 MHz, $CDCl_3$): δ = 7.93–7.24 (m, 32 $H_{arom}$), 1.87 (s, $CH_3$ *cis*), 1.74 (s, 2 $CH_3$ *trans*), 1.36 (s, $CH_3$ *cis*) ppm. $^{13}$C NMR (150 MHz, $CDCl_3$): δ = 144.6 br., 143.2 br. (for 8 $C_{arom}$), 133.1, 132.9, 132.8, 131.6, 131.2, 130.8, 128.0, 127.9, 127.6, 127.1, 127.0, 126.7 (for 32 $CH_{arom}$), 80.5, 80.0 (C-4 + C-5, for *cis* and *trans*), 55.6, 55.3 (C-2, *cis* and *trans*), 33.2 (2 $CH_3$ *trans*), 33.0 ($CH_3$ *cis*), 32.7 ($CH_3$ *cis*) ppm. $C_{25}H_{22}S_2Se_2$ (544.49): calcd. C 55.15, H 4.07, S 11.78; found C 55.32, H 4.26, S 11.63.

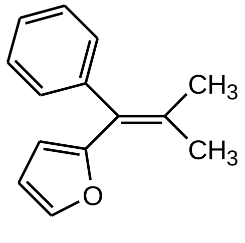


*1-Phenyl-1-(furan-2-yl)-2-methylpropene* (**9g**) [S2b]: Yield 79 mg (40%) – After desulfurization of tiirane **8g**. Pale yellow oil (lit. [S2b]: a liquid isolated by distillation under reduced pressure). $^1$H NMR (600 MHz, $CDCl_3$): δ = 7.29–7.39 (m, 4 $H_{arom}$), 7.20–7.21 (m, 2 $H_{arom}$), 6.37–6.38 (m, 1 $H_{arom}$), 5.96 (d, *J* = 3.0 Hz, 1 $H_{arom}$), 2.17 (s, $CH_3$), 1.75 (s, $CH_3$) ppm. $^{13}$C NMR (150 MHz, $CDCl_3$): δ = 155.5, 141.2, 133.3, 127.3 (2 $C_{arom}$, $C_{arom}$*C*=*C*($CH_3$)$_2$), 140.9, 129.9, 128.0, 126.6, 110.4, 109.4 (for 8 $CH_{arom}$), 23.3 (*C*$H_3$), 22.4 ($CH_3$) ppm.

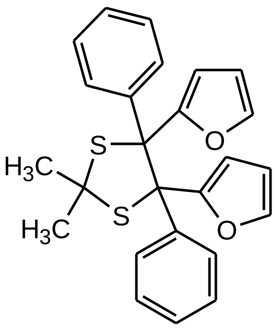

*2,2-Dimethyl-4,5-diphenyl-4,5-di(furan-2-yl)-1,3-dithiolane* (**10g**): Isolated as a mixture of *cis*,*trans*-isomers (crude product ratio 58:42). Yield: 59 mg (28%). White crystals; m.p. 144 – 145 °C (MeOH/$CHCl_3$). IR (KBr): ν = 3110 (w), 3058 (w), 2921 (m), 2855 (w), 1598 (w), 1490 (s), 1445 (s), 1149 (s), 1037 (m), 1017 (s), 932 (m), 795 (m), 750 (s), 718 (s), 696 (s), 595 (m) $cm^{-1}$. $^1H$ NMR (600 MHz, $CDCl_3$): δ = 7.37–5.94 (*m*, 32 $CH_{arom}$) 1.99 (s, $CH_3$ *cis*), 1.72 (s, 2 $CH_3$ *trans*), 1.53 (s, $CH_3$ *cis*) ppm. $^{13}C$ NMR (150 MHz, $CDCl_3$): δ = 155.0, 154.9, 140.0, 139.9 (for 8 $C_{arom}$), 141.0, 140.6, 130.5, 129.8, 127.6, 127.3, 127.2, 126.5, 112.9, 110.9, 110.3, 110.1 (for 32 $CH_{arom}$), 76.1, 76.0 (C-4 + C-5, for *cis* and *trans*), 55.8, 54.9 (C-2, *cis* and *trans*), 34.8 ($CH_3$ *cis*), 33.7 (2 $CH_3$ *trans*), 32.3 ($CH_3$ *cis*) ppm. $C_{25}H_{22}O_2S_2$ (418.57): calcd. C 71.74, H 5.30, S 15.32; found C 71.87, H 5.28, S 15.54.

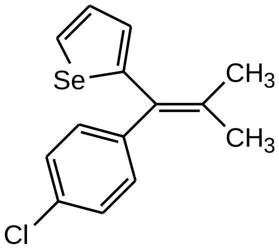

*1-(4-Chlorophenyl)-1-(selenophen-2-yl)-2-methylpropene* (**9h**): Yield: 168 mg (65%) – After desulfurization of tiirane **8h**. Yellow oil. IR (film): ν = 3056 (w), 2908 (m), 2852 (m), 1901 (w), 1590 (w), 1488 (s), 1448 (m), 1229 (m), 1218 (m), 1091 (s), 1014 (s), 855 (m), 833 (m), 817 (s), 776 (s), 685 (s) $cm^{-1}$. $^1H$ NMR (600 MHz, $CDCl_3$): δ = 7.95–7.96 (m, 1 $H_{arom}$), 7.16–7.33 (m, 5 $H_{arom}$), 6.96–6.97 (m, 1 $H_{arom}$), 2.05 (s, $CH_3$), 1.77 (s, $CH_3$) ppm. $^{13}C$ NMR (150 MHz, $CDCl_3$): δ = 151.3, 142.0, 133.5, 132.4, 131.1 (3 $C_{arom}$, $C_{arom}$*C*=*C*($CH_3$)$_2$), 130.9, 130.6, 129.1, 128.9, 128.3 (for 7 $CH_{arom}$), 22.8 ($CH_3$), 22.3 ($CH_3$) ppm. $C_{14}H_{13}ClSe$ (259.67): calcd. C 56.87, H 4.43; found C 56.96, H 4.55.

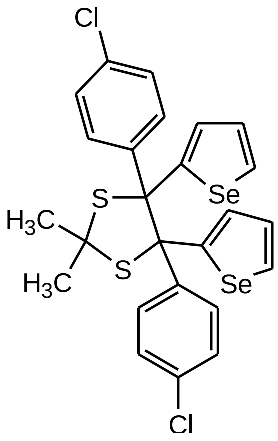


*2,2-Dimethyl-4,5-di(4-chlorophenyl)-4,5-di(selenophen-2-yl)-1,3-dithiolane* (**10h**): Isolated as a mixture of *cis*,*trans*-isomers (crude product ratio 70:30). Yield: 53 mg (17%). White crystals; m.p. 86 – 87 °C (MeOH/$CHCl_3$). IR (KBr): ν = 2919 (w), 2851 (w), 1587 (w), 1489 (s), 1230 (m), 1095 (s), 1012 (s), 785 (m), 688 (s) $cm^{-1}$. $^1H$ NMR (600 MHz, $CDCl_3$): δ = 7.94–6.98 (m, 28 $H_{arom}$), 1.85 (s, $CH_3$ *cis*), 1.72 (s, 2 $CH_3$ *trans*), 1.44 (s, $CH_3$ *cis*) ppm. $^{13}C$ NMR (150 MHz, $CDCl_3$): δ = 157.3, 156.6, 142.6, 141.9 (for 8 $C_{arom}$), 133.6, 133.2, 133.1, 132.9, 132.8, 132.5, 132.3, 132.1, 128.0, 127.1, 126.9 (for 32 $CH_{arom}$), 79.7, 79.4 (C-4 + C-5, for *cis* and *trans*), 56.0, 55.9 (C-2, *cis* and *trans*), 33.1 ($CH_3$ *cis*), 33.0 (2 $CH_3$ *trans*), 32.7 ($CH_3$ *cis*) ppm. $C_{25}H_{20}Cl_2S_2Se_2$ (613.38): calcd. C 48.95, H 3.29, S 10.45; found C 48.73, H 3.50, S 10.48.

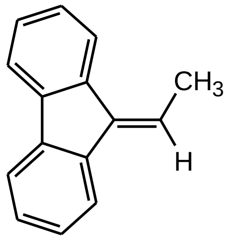


*9-Ethylidenefluorene* (**9j**) [S3]: Yield: 6 mg (6%). Pale yellow crystals; m.p. 97 – 98 °C (isolated by PLC). (Lit. [S3a], m.p. 76.5 – 78.0); lit. [S3b], m. p. 103 – 104 °C). $^1H$ NMR (600 MHz, $CDCl_3$): δ = 7.92 (d, *J* = 7.2 Hz, 1 $H_{arom}$), 7.79 (d, *J* = 7.2 Hz, 1 $H_{arom}$), 7.73 (d, *J* = 7.2 Hz, 1 $H_{arom}$), 7.68 (d, *J* = 7.2 Hz, 1 $H_{arom}$), 7.30–7.40 (m, 4 $H_{arom}$), 6.89 (q, *J* = 7.2 Hz, =CH), 2.43 (d, *J* = 7.2 Hz, $CH_3$) ppm.

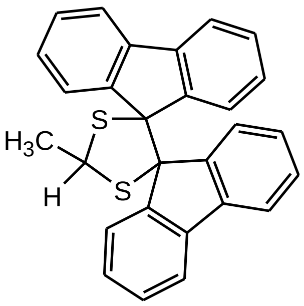


*2’-Methyldispiro[9*H*-fluorene-9,4’-(1,3)dithiolane-5’9’’-[9*H*]fluorene]* (**10j**): Yield: 65 mg (61%). White crystals; m.p. 251 – 252 °C (MeOH/$CHCl_3$). IR (KBr): $\nu$ = 3052 (w), 3028 (w), 1474 (w), 1445 (m), 1279 (w), 1157 (w), 1033 (w), 739 (s) $cm^{-1}$. $^1H$ NMR (600 MHz, $CDCl_3$): $\delta$ = 7.55–7.28 (m, 8 $H_{arom}$), 7.16–7.11 (m, 4 $H_{arom}$), 7.00 (s br., 4 $H_{arom}$), 5.51 (q, $J$ = 6.0 Hz, =CH) 2.00 (d, $J$ = 6.0 Hz, $CH_3$) ppm. $^{13}C$ NMR (150 MHz, $CDCl_3$): $\delta$ = 140.2, 140.0 (for 8 $C_{arom}$), 128.5, 128.4, 127.2, 126.6, 126.5, 126.4, 119.4, 119.1 (for 16 $CH_{arom}$), 76.1 (C-4, C-5), 46.8 (C-2), 22.2 ($CH_3$) ppm. $C_{28}H_{20}S_2$ (420.59): calcd. C 79.96, 4.79, 15.25; found C 79.73, H 4.77, S 15.15.

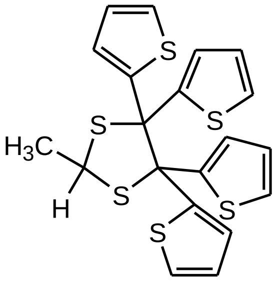


*2-Methyl-4,4,5,5-tetra(thiophen-2-yl)-1,3-dithiolane* (**10k**): Yield: 160 mg (72%). Yellow crystals; m.p. 155 – 156 °C (MeOH/$CHCl_3$). IR (KBr): $\nu$ = 3097 (w), 3070 (w), 2918 (w), 1424 (m), 1227 (s), 1187 (w), 1045 (m), 856 (m), 753 (m), 703 (s) $cm^{-1}$. $^1H$ NMR (600 MHz, $CDCl_3$): $\delta$ = 7.23–7.25 (m, 4 $H_{arom}$), 7.09–7.10 (m, 2 $H_{arom}$), 6.89–6.90 (m, 2 $H_{arom}$), 6.85–6.87 (m, 4 $H_{arom}$), 4.77 (q, $J$ = 6.6 Hz, C*H*($CH_3$)), 1.79 (d, $J$ = 6.6 Hz, $CH_3$) ppm. $^{13}C$ NMR (150 MHz, $CDCl_3$): $\delta$ = 146.2, 147.4 (for 4 $C_{arom}$), 130.3, 129.4, 126.9, 126.1, 125.8, 125.6 (for 12 $CH_{arom}$), 74.8 (C-4, C-5), 45.0 (C-2), 18.9 ($CH_3$) ppm. $C_{20}H_{16}S_6$ (448.73): calcd. C 53.53, H 3.59, S 42.87; found C 53.62, H 3.83, S 42.88.

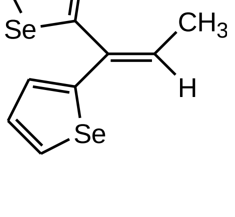

*1,1-Di(selenophen-2-yl)propene* (**9l**): Yield: 10 mg (3 %). Pale yellow oil. IR (film): ν = 3055 (w), 2926 (w), 1724 (w), 1444 (m), 1246 (m), 1221 (m), 1022 (w), 841 (w), 816 (m), 793 (w), 742 (w), 684 (s) $cm^{-1}$. $^{1}H$ NMR (600 MHz, $CDCl_3$): δ = 8.07–8.08 (m, 1 $H_{arom}$), 7.79–7.80 (m, 1 $H_{arom}$), 7.30–7.31 (m, 1 $H_{arom}$), 7.14–7.17 (m, 2 $H_{arom}$), 7.02–7.03 (m, 1 $H_{arom}$), 6.24 (q, *J* = 7.2 Hz, =C*H*), 1.82 (d, *J* = 7.2 Hz, $CH_3$) ppm. $^{13}C$ NMR (150 MHz, $CDCl_3$): δ = 153.3, 145.9, 133.7 (2 $C_{arom}$, $C_{arom}$-*C*=), 131.3, 129.9, 129.8, 129.1, 128.7, 127.0, 126.6 (6 $CH_{arom}$, =*C*H), 15.9 ($CH_3$) ppm. $C_{11}H_{10}Se_2$(300.12): calcd. C 44.02, H 3.36; found C 44.00, H 3.66.

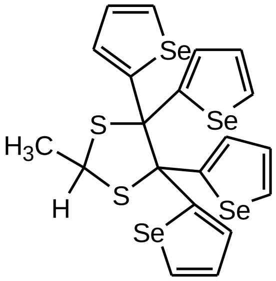


*2-Methyl-4,4,5,5-tetra(selenophen-2-yl)-1,3-dithiolane* (**10l**): Yield: 252 mg (79%). White crystals; m.p. 152 – 153 °C (MeOH/$CHCl_3$). IR (KBr): ν = 3088 (w), 3048 (w), 2914 (w), 1439 (m), 1375 (w), 1230 (s), 1022 (m), 829 (w), 787 (w), 682 (s) $cm^{-1}$. $^{1}H$ NMR (600 MHz, $CDCl_3$): δ = 7.99–8.01 (m, 4 $H_{arom}$), 7.47–7.48 (m, 2 $H_{arom}$), 7.17–7.19 (m, 2 $H_{arom}$), 7.10–7.13 (m, 4 $H_{arom}$), 4.8 (q, *J* = 6.6 Hz, C*H*($CH_3$)), 1.76 (d, *J* = 6.6 Hz, $CH_3$) ppm. $^{13}C$ NMR (150 MHz, $CDCl_3$): δ = 154.5, 152.7 (for 4 $C_{arom}$), 134.1, 133.2 br., 132.3, 128.2, 128.0 (for 12 $CH_{arom}$), 78.4 (C-4, C-5), 45.6 (C-2), 19.1 ($CH_3$) ppm. $C_{20}H_{16}S_2Se_4$ (636.31): calcd. C 37.75, H 2.53, S 10.08; found C 37.67, H 2.65, S 10.23.

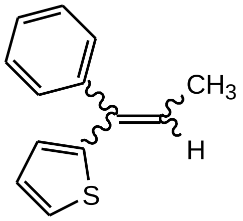


*1-Phenyl-1-(thiophen-2-yl)propene* (**9m**): Isolated as a mixture of (*E*/*Z*)-isomers (53:47). Yield: 96 mg (48%) – After desulfurization of tiirane **8m**. Yellow oil. IR (film): ν = 3062 (w), 2931 (w), 1716 (m), 1682 (m), 1635 (s), 1599 (m), 1578 (m), 1515 (m), 1447 (m), 1413 (s), 1354 (m), 1288 (s), 1232 (m), 1053 (m), 843 (m), 717 (s), 700 (s), 649 (m) $cm^{-1}$. $^{1}H$ NMR (600 MHz, $CDCl_3$): δ = 6.48–7.32 (m, 16 $H_{arom}$), 6.17 (q, *J* = 7.2 Hz, =CH), 6.02 (q, *J* = 7.2 Hz, =CH), 1.86 (d, *J* = 7.2 Hz, $CH_3$), 1.60 (d, *J* = 7.2

Hz, $CH_3$) ppm. $^{13}C$ NMR (150 MHz, $CDCl_3$): δ = 147.4, 143.2, 141.6, 139.1, 136.7, 135.8 (4 $C_{arom}$, 2 $C_{arom}$-*C*=), 129.7, 128.2, 128.0, 127.8, 127.5, 127.3, 127.1, 126.8, 126.6, 125.4, 124.6, 123.4, 123.1 (for 16 $CH_{arom}$, 2 (=CH)), 16.0 ($CH_3$), 15.2 ($CH_3$). $C_{13}H_{12}S$ (200.30): calcd. C 77.95, H 6.04, S 16.01; found C 78.0, H 5.79, S 16.29.

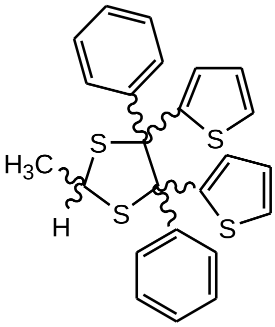


*2-Methyl-4,5-diphenyl-4,5-di(thiophen-2-yl)-1,3-dithiolane* (**10m**): Isolated as a mixture of two *cis,trans*-isomers in a ratio of 56:44 (from three isomers possible). Yield: 58 mg (27%). Yellow crystals; m.p. 137 – 139 °C (MeOH/$CHCl_3$). IR (KBr): ν = 3055 (w), 2922 (w), 1597 (w), 1488 (s), 1443 (s), 1371 (w), 1233 (w), 1187 (m), 1052 (m), 1034 (m), 850 (w), 695 (s) $cm^{-1}$. $^{1}H$ NMR (600 MHz, $CDCl_3$): δ = 6.48–7.54 (m, 32 $H_{arom}$), 4.38 (q, *J* = 6.6 Hz, C*H*($CH_3$)), 4.09 (q, *J* = 6.6 Hz, C*H*($CH_3$)), 1.68 (d, *J* = 6.6 Hz, $CH_3$), 1.61 (d, *J* = 6.6 Hz, $CH_3$) ppm. $^{13}C$ NMR (150 MHz, $CDCl_3$): δ = 151.1, 149.8, 148.7, 143.4, 143.3, 141.3 (for 8 $C_{arom}$), 131.2, 130.8, 130.6, 130.4, 130.3, 130.2, 127.7, 127.3, 127.2, 126.8, 126.7, 126.4, 126.2, 126.1, 125.5, 125.4, 125.3, 124.9 (for 32 $CH_{arom}$), 77.7, 77.6, 76.4, 76.9 (2 C-4, 2 C-5), 43.3, 43.1 (2 C-2), 17.8 ($CH_3$), 18.7 ($CH_3$) ppm. $C_{24}H_{20}S_4$ (436.68): calcd. C 66.01, H 4.62, S 29.37; found C 66.00, H 4.71, S 29.27.

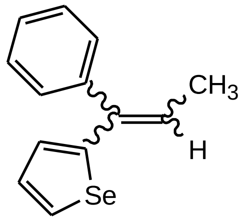


*1-Phenyl-1-(selenophen-2-yl)propene* (**9n**): Isolated as a mixture of (*E*/*Z*)-isomers (73:27). Yield: 176 mg (71%) – After desulfurization of tiirane **8n**. Red oil. IR (film): ν = 3055 (w), 3020 (w), 2928 (w), 2909 (w), 2852 (w), 1491 (m), 1441 (m), 1220 (m), 819 (m), 787 (m), 702 (s), 685 (s). $^{1}H$ NMR (600 MHz, $CDCl_3$): δ = 6.70–8.04 (m, 16 $H_{arom}$), 6.17 (q, *J* = 7.2 Hz, =CH), 6.09 (q, *J* = 7.2 Hz, =CH), 1.93 (d, *J* = 7.2 Hz, $CH_3$), 1.65 (d, *J* = 7.2 Hz, $CH_3$) ppm. $^{13}C$ NMR (150 MHz, $CDCl_3$): δ = 153.7, 147.8, 143.4,

139.2, 138.8, 137.9 (4 $C_{arom}$, 2 $C_{arom}C$=), 131.1, 130.0, 129.9, 129.6, 129.1, 128.2, 128.1, 128.0, 127.6, 127.2, 127.1, 126.0, 124.2 (for 16 $CH_{arom}$, 2 =*C*H), 16.0 ($CH_3$), 15.4 ($CH_3$). $C_{13}H_{12}Se$ (247.19): calcd. C 63.16, H 4.89; fund C 62.98, 4.84.

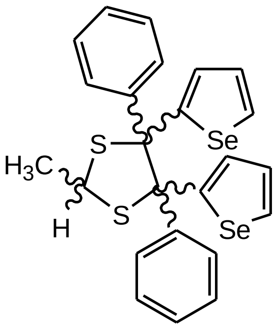


*2-Methyl-4,5-diphenyl-4,5-di(selenophen-2-yl)-1,3-dithiolane* (**10n**): Isolated as a mixture of two *cis*/*trans*-isomers in a ratio of 52:48 (from three isomers possible). Yield: 74 mg (28%). Yellow crystals: m.p. 144 – 146 °C (MeOH/$CHCl_3$). IR (KBr): ν = 3053 (w), 2922 (w), 1596 (w), 1486 (s), 1441 (s), 1371 (m), 1232 (s), 1185 (m), 1032 (m), 685 (s) $cm^{-1}$. $^1H$ NMR (600 MHz, $CDCl_3$): δ 6.63–7.92 (m, 32 $H_{arom}$), 4.54 (q, *J* = 6.6 Hz, C*H*($CH_3$)), 4.12 (q, *J* = 6.6 Hz, C*H*($CH_3$)), 1.78 (d, *J* = 6.6 Hz, $CH_3$), 1.68 (d, *J* = 6.6 Hz, $CH_3$) ppm. $^{13}C$ NMR (150 MHz, $CDCl_3$): δ = 160.2, 157.9, 156.2, 143.7, 143.4, 140.9 (for 8 $C_{arom}$), 133.2, 132.7, 132.6, 132.5, 132.4, 131.6, 131.0, 130.6, 130.3, 128.2, 128.0, 127.8, 127.6, 127.5, 127.3, 126.9, 126.8, 126.7 (for 32 $CH_{arom}$), 79.3, 79.2, 78.7, 78.1 (2 C-4, 2 C-5), 43.4, 43.3 (2 C-2), 18.6 ($CH_3$), 17.7 ($CH_3$) ppm. $C_{24}H_{20}S_2Se_2$ (530.47): calcd. C 54.34, H 3.80, S 12.09; found C 54.39, H 3.77, 12.19.

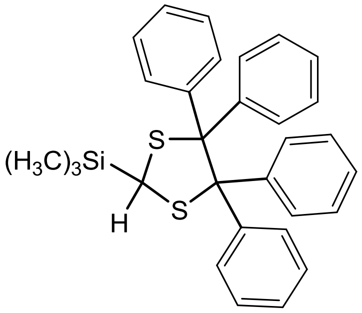


*4,4,5,5-Tetraphenyl-2-trimethylsilyl-1,3-dithiolane* (**10o**): Yield: 79 mg (33%). Colorless crystals: m.p. 87 – 88 °C ($CH_3OH/CH_2Cl_2$). IR (KBr): ν = 3054 (w), 2952 (w), 2842 (w), 1603 (w), 1494 (m), 1437 (m), 1246 (m), 864 (s), 835 (s), 722 (s), 694 (s) $cm^{-1}$. $^1H$ NMR (600 MHz, $CDCl_3$): 6.94–7.64 (m, 20 $H_{arom}$), 3.36 (s, (($CH_3)_3Si$)*H*C), 0.34 (s, $(CH_3)_3Si$) ppm. $^{13}C$ NMR (150 MHz, $CDCl_3$): 142.5 (for 4 $C_{arom}$), 131.7 br., 131.3 br., 129.2, 128.1, 127.8, 126.4 br., 126.1 br. (for 20 $CH_{arom}$), 79.5 (C-4,C-5),

33.4 (C-2), -1.55 (($CH_3$)$_3$Si) ppm. HR-EI-MS: 482.155825 (calcd. 482.154830 for $C_{30}H_{30}S_2Si$ $[M]^+$).

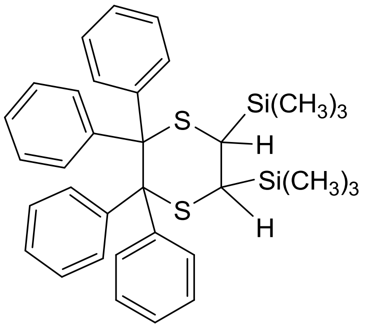


*2,2,3,3-Tetraphenyl-5,6-bis(trimethylsilyl)-1,4-dithiane* (**4c**) (in analogy to **4d** tentatively described as a *trans*-isomer): Yield: 65 mg (22%). Colorless crystals: m.p. 185 – 186 °C ($CH_3OH/CH_2Cl_2$). IR (KBr): ν = 3058 (w), 2941 (w), 2860 (w), 1487 (m), 1434 (m), 1250 (s), 1108 (w), 857 (s), 718 (s), 694 (s) $cm^{-1}$. $^1H$ NMR (600 MHz, $CDCl_3$): 7.56–7.57 (m, 4 $H_{arom}$), 7.32–7.33 (m, 4 $H_{arom}$), 7.03–7.17 (m, 12 $H_{arom}$), 2.97 (s, (2 ($CH_3$)$_3$Si)*H*C), 0.14 (s, 2 ($CH_3$)$_3$Si) ppm. $^{13}C$ NMR (150 MHz, $CDCl_3$): 146.7, 145.2 (for 4 $C_{arom}$), 135.0, 132.0, 126.5, 126.2, 125.8, 124.9 (for 20 $CH_{arom}$), 63.9 (C-5, C-6), 36.3 (2 CHSi), 0.17 (2 ($CH_3$)$_3$Si) ppm. $C_{34}H_{40}S_2Se_2$ (568.98): calcd. C 71.77, H 7.09, S 11.27; found C 71.77, H 7.13, S 11.38.ESI-MS (MeOH): 591(100, [*M*+Na].

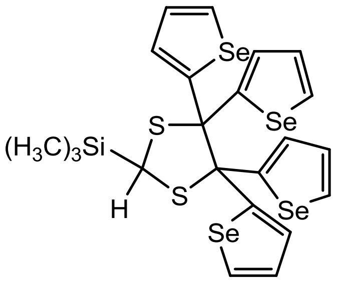


*4,4,5,5-Tetra(selenophen-2-yl)-2-trimethylsilyl-1,3-dithiolane* (**10s**): Yield: 265 mg (76%). Yellow crystals: m.p. 140 – 142 °C (hexane/$CH_2Cl_2$). IR (KBr): ν = 2949 (w), 1439 (m), 1251 (s), 1225 (s), 1156 (w), 1120 (m), 1104 (w), 844 (s), 805 (m), 694 (s) $cm^{-1}$. $^1H$ NMR (600 MHz, $CDCl_3$): 7.09–7.10 (m, 2 $H_{arom}$), 7.14–7.16 (m, 2 $H_{arom}$), 7.19–7.20 (m, 2 $H_{arom}$), 7.51–7.52 (m, 2 $H_{arom}$), 7.93–7.93 (m, 4 $H_{arom}$), 4.02 (s, (($CH_3$)$_3$Si)*H*C), 0.31 (s, ($CH_3$)$_3$Si) ppm. $^{13}C$ NMR (150 MHz, $CDCl_3$): 156.0, 153.3 (for 4 arom. C), 133.8, 132.8, 132.2, 131.9, 128.3, 128.1 (for 12 $CH_{arom}$), 78.2 (C-4, C-5), 37.4 (C-2), –1.46 (($CH_3$)$_3$Si) ppm. $C_{22}H_{22}S_2Se_4Si$ (694.47): calcd. C 38.05, H 3.19, S 9.23; found C 38.08, H 3.21, 9.55.

# References


[S1] Rabinovitz, M.; Agranat, I.; Bergrnann, E. D., *J. Chem. Soc.*, **1967**, 1281–1284. doi: 10.1039/J29670001281.

[S2] a) Ganapathy, D.; Sekar G.; *Org. Lett*. **2014**, *16*, 3856–3859, doi: 10.1021/ol5017367 b) Cantrell, T. S.; *J. Org. Chem*., **1974**, *3*, 2242–2246. doi: 10.1021/jo00929a024.

[S3] a) Bell, T. W.; Catalano, V. J.; Drew, M. G. B.; Phillips, D. J.; *Chem. Eur. J*., **2002**, 8, 5001–5006, doi: 10.1002/1521-3765. b) Boyce, R.; Hayes, B. A.; Murphy, W. S., *J. Chem. Soc*. *Perkin Trans. 1*, **1975**, 531–534. doi: 10.1039/P19750000531.


## **2.** Collection of the $^{1}$H- and $^{13}$C-NMR for the described compounds **8**, **9**, and **10**

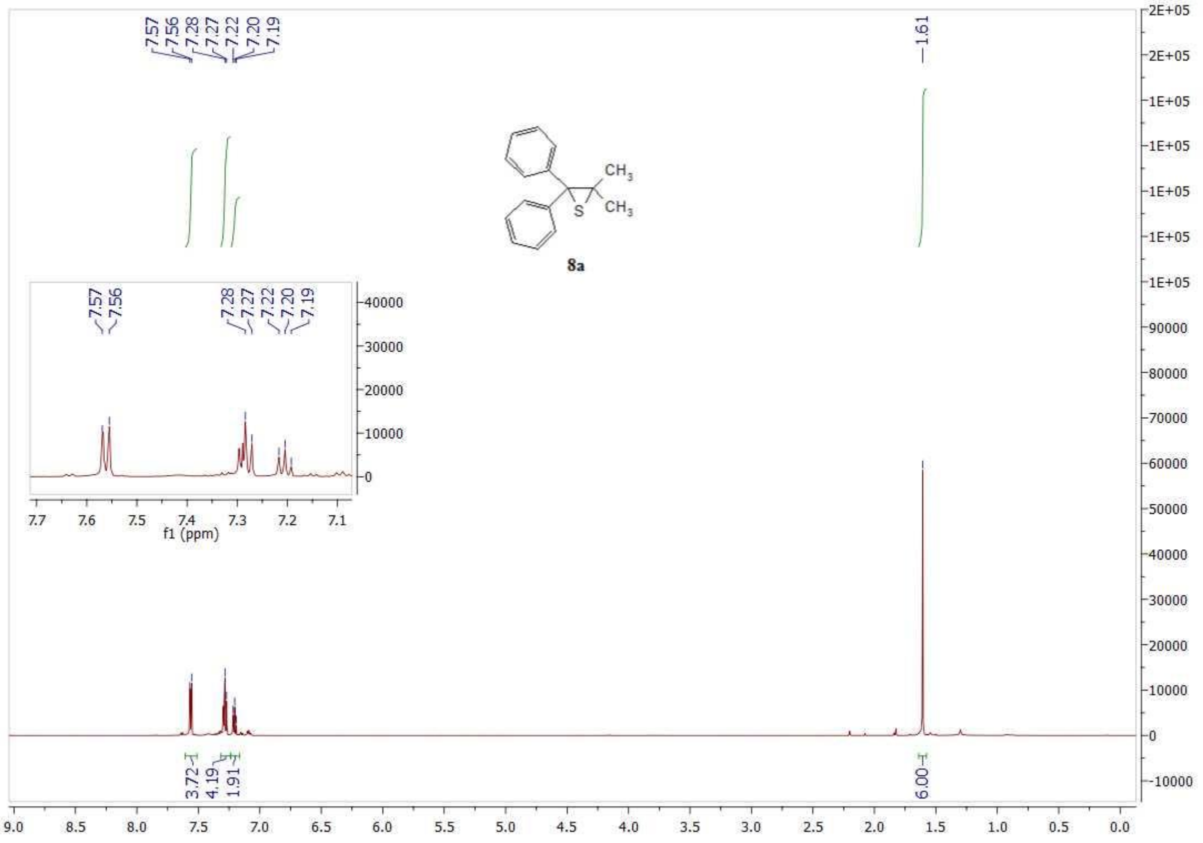


**Figure S1:** The $^{1}$H NMR spectrum of compound **8a**.

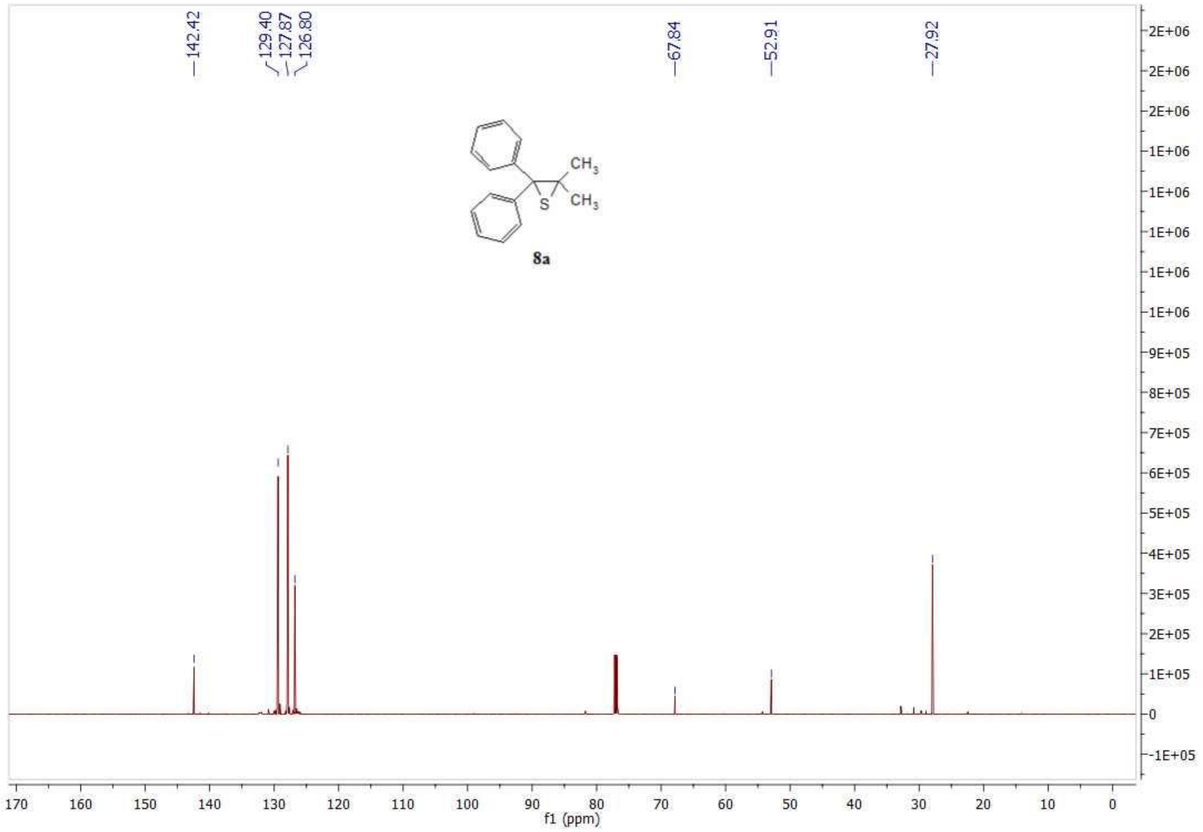

**Figure S2:** The $^{13}C$ NMR spectrum of compound **8a**.

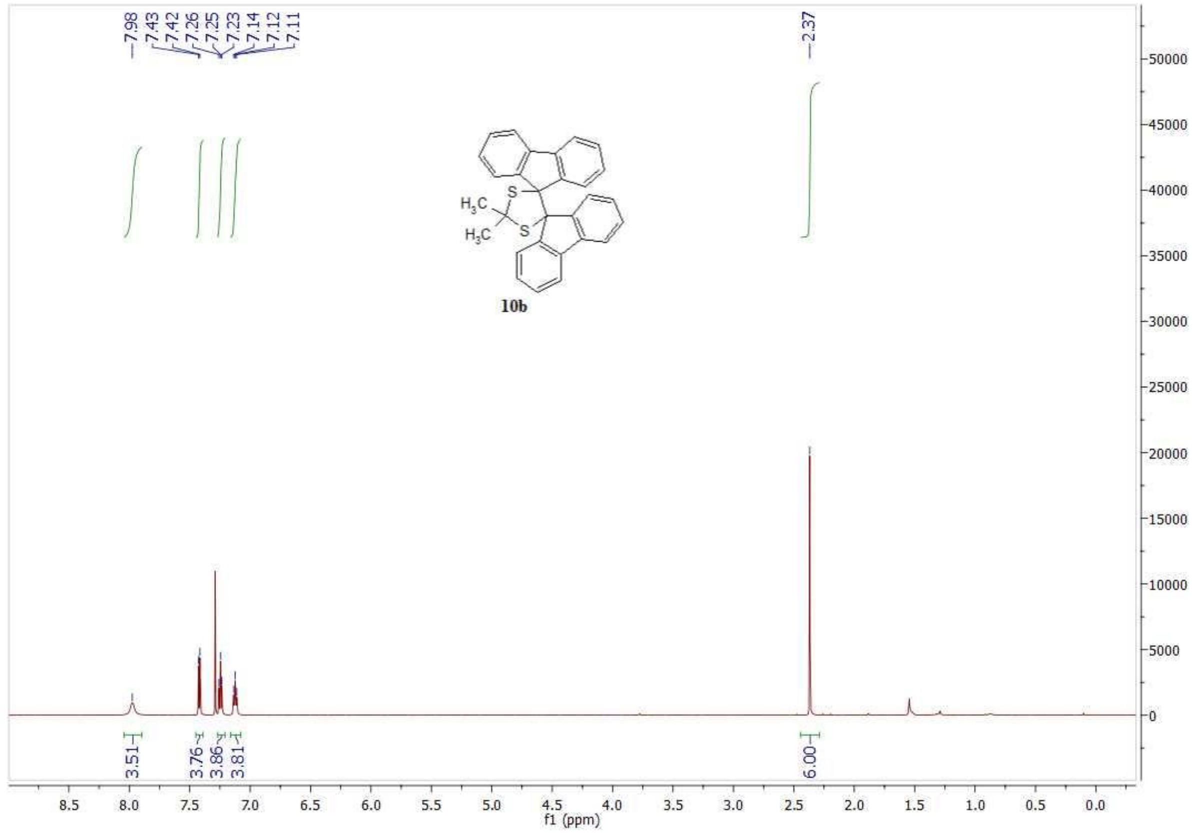

**Figure S3:** The $^{1}H$ NMR spectrum of compound **10b**.

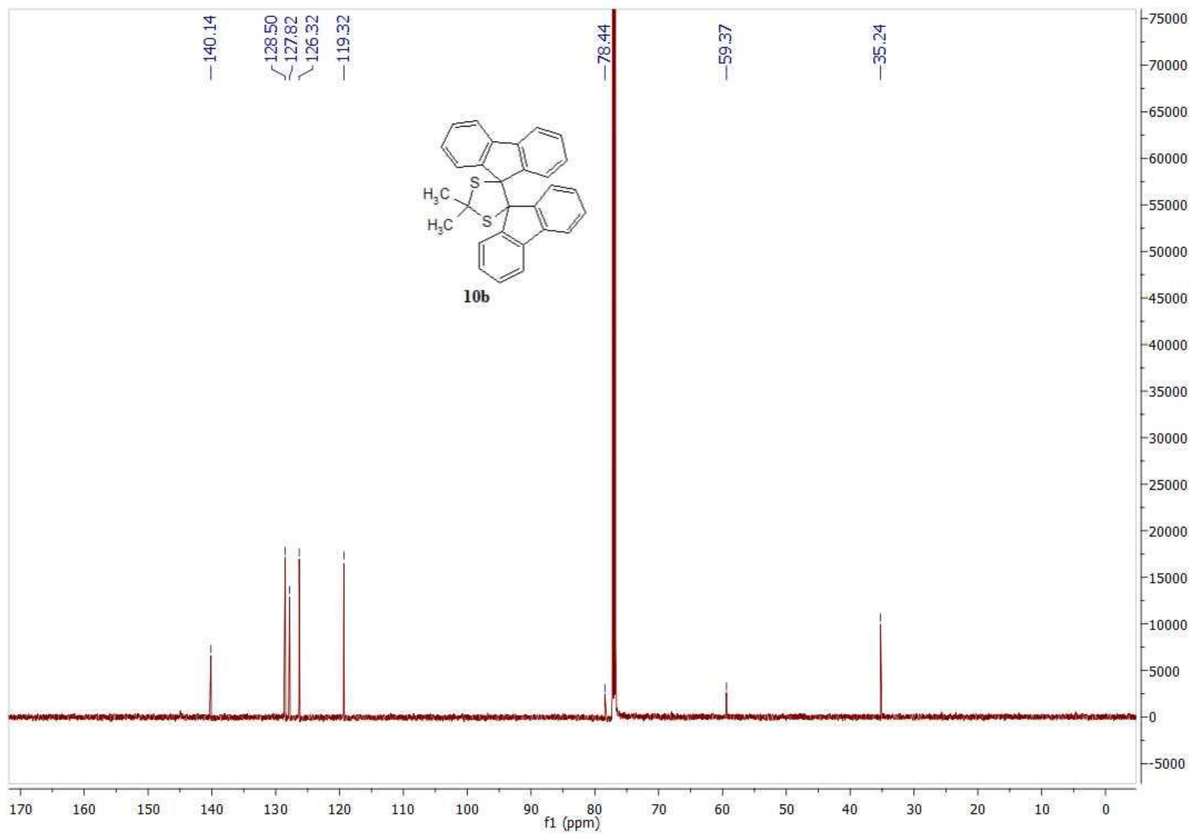


**Figure S4:** The $^{13}$C NMR spectrum of compound **10b**.

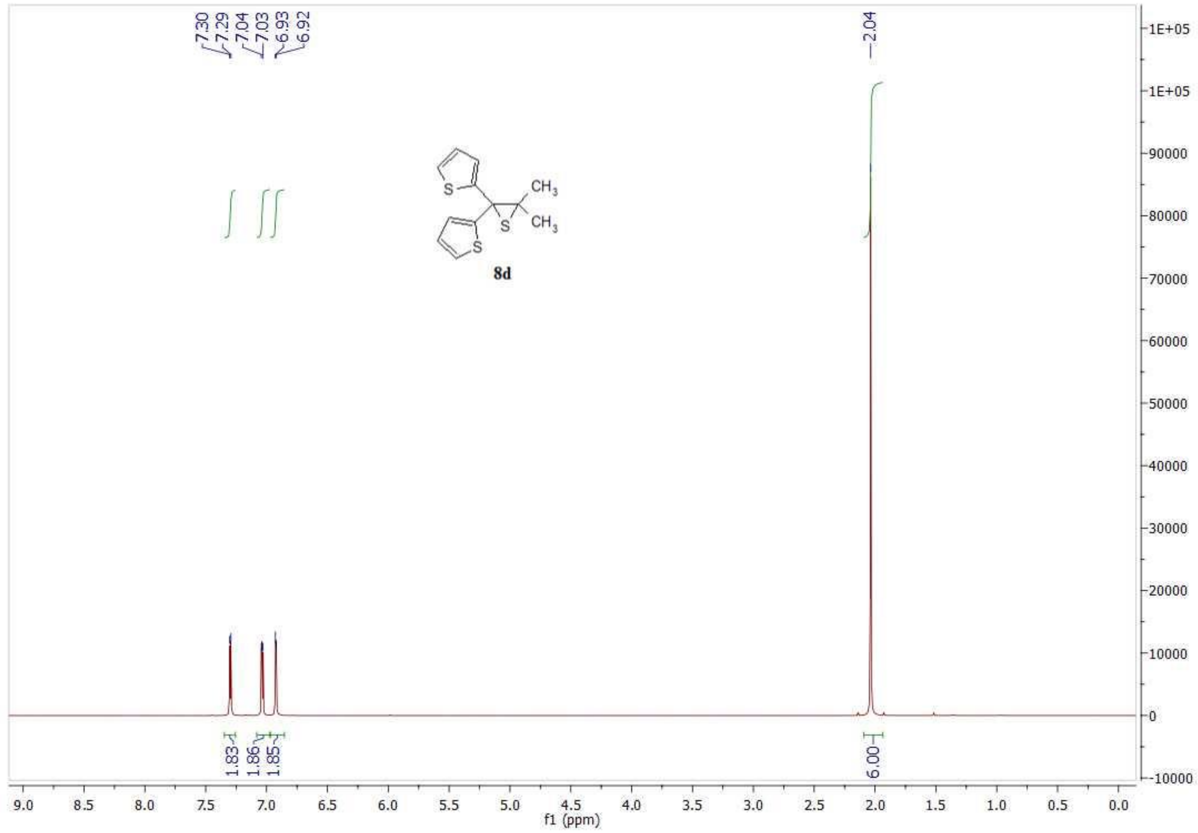


**Figure S5:** The $^{1}$H NMR spectrum of compound **8d**.

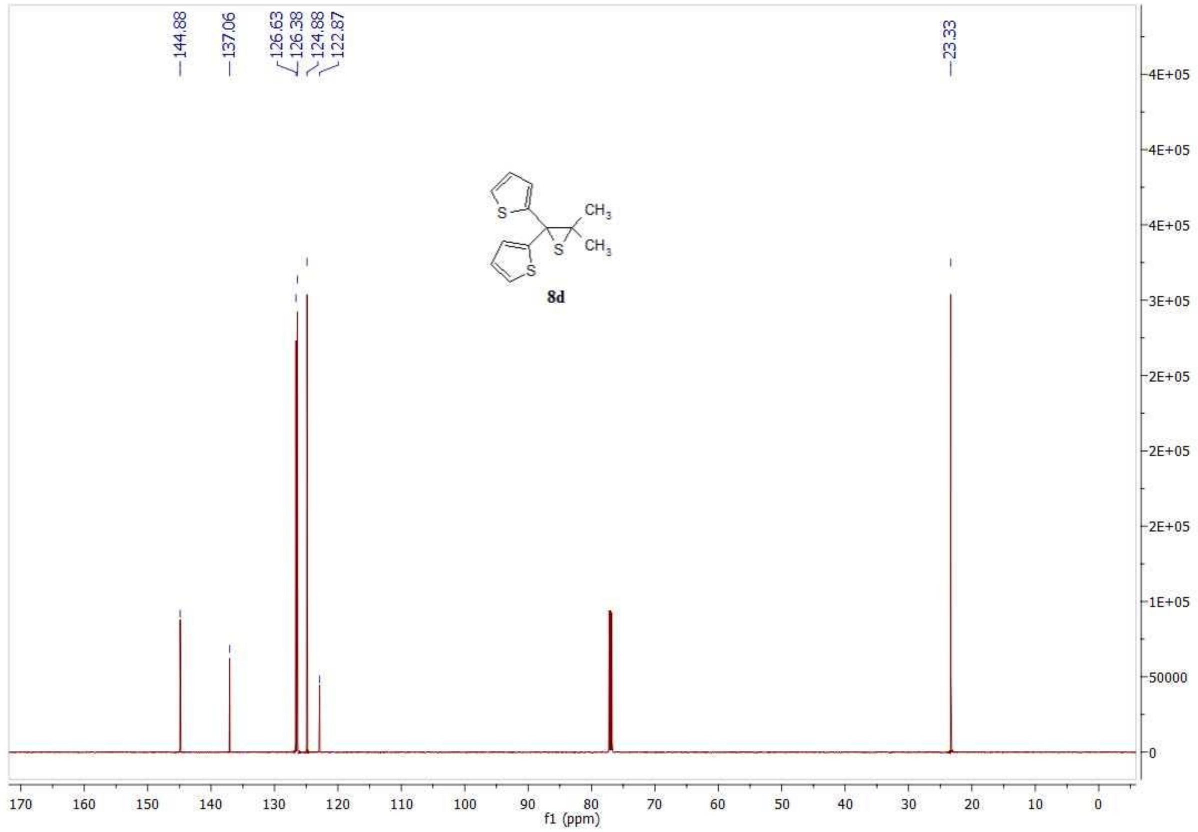


**Figure S6:** The $^{13}$C NMR spectrum of compound **8d**.

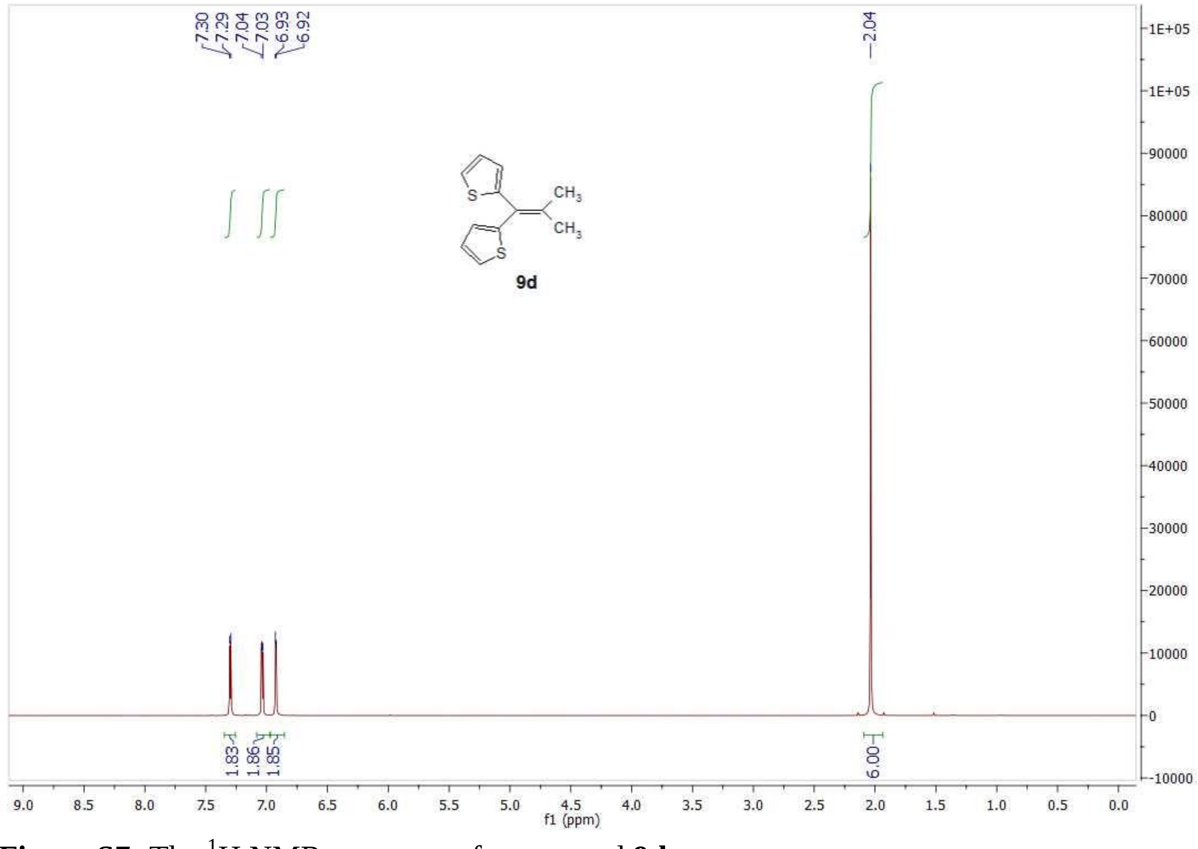


**Figure S7:** The $^1$H NMR spectrum of compound **9d**.

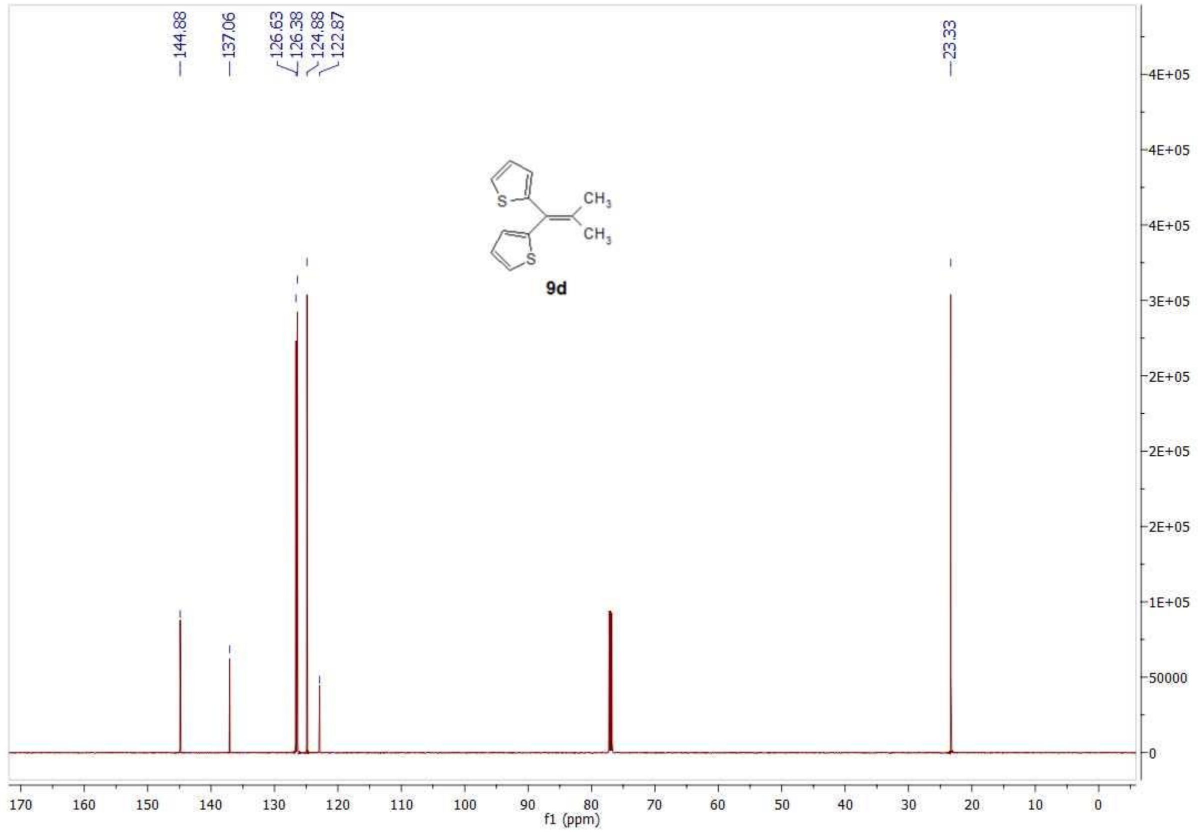


**Figure S8:** The $^{13}$C NMR spectrum of compound **9d**.

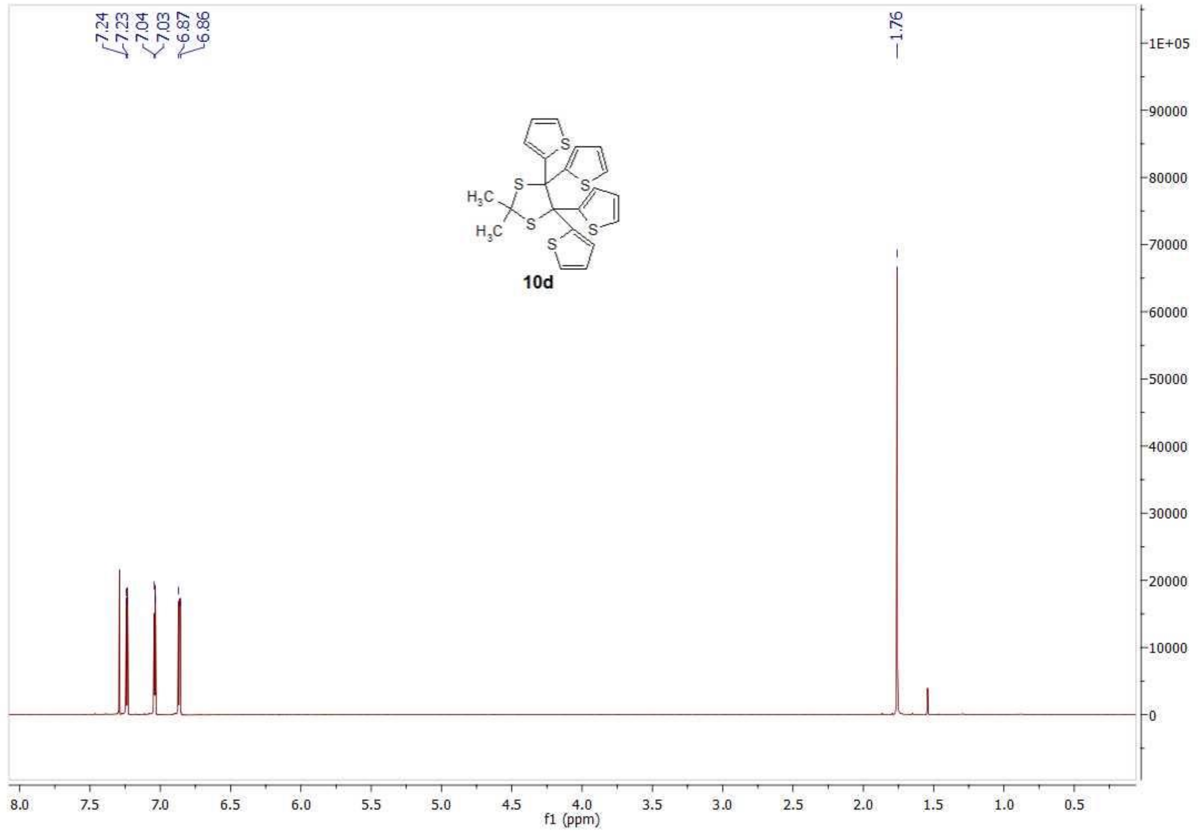

**Figure S9:** The $^{1}H$ NMR spectrum of compound **10d**.

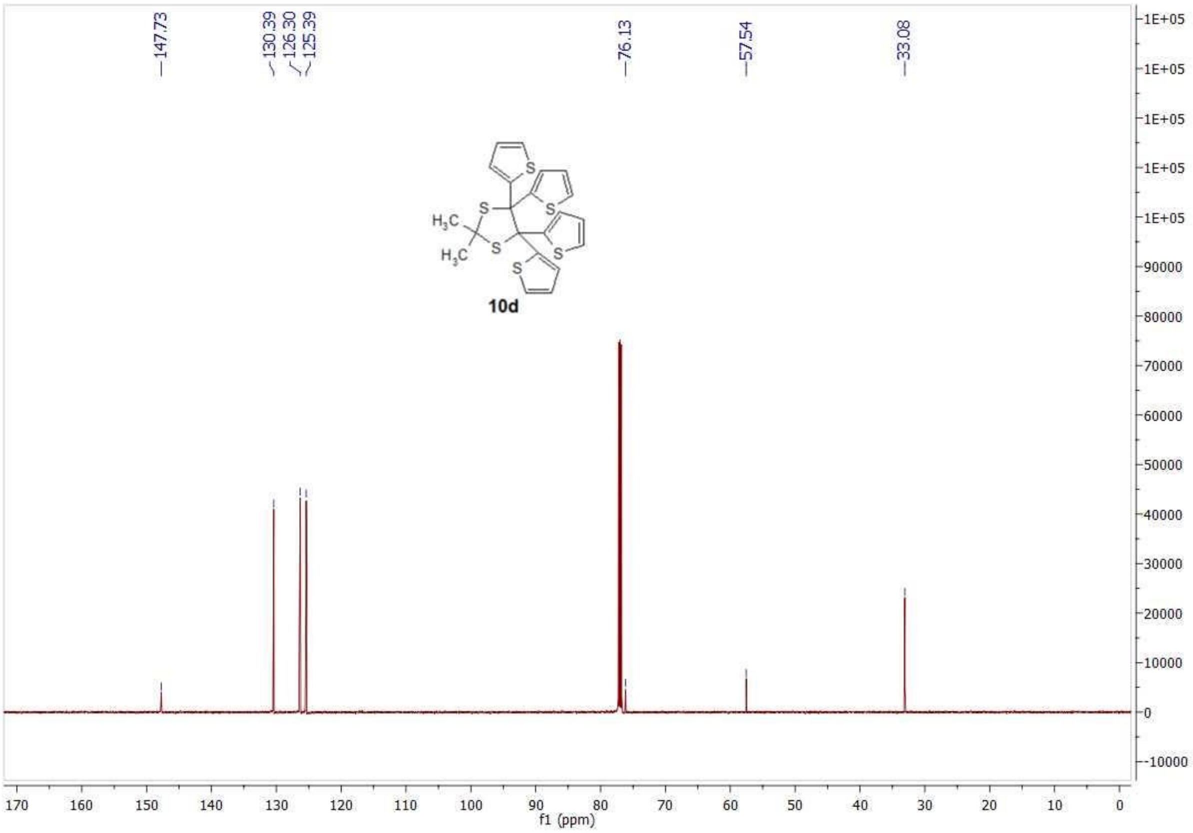


**Figure S10:** The $^{13}C$ NMR spectrum of compound **10d**.

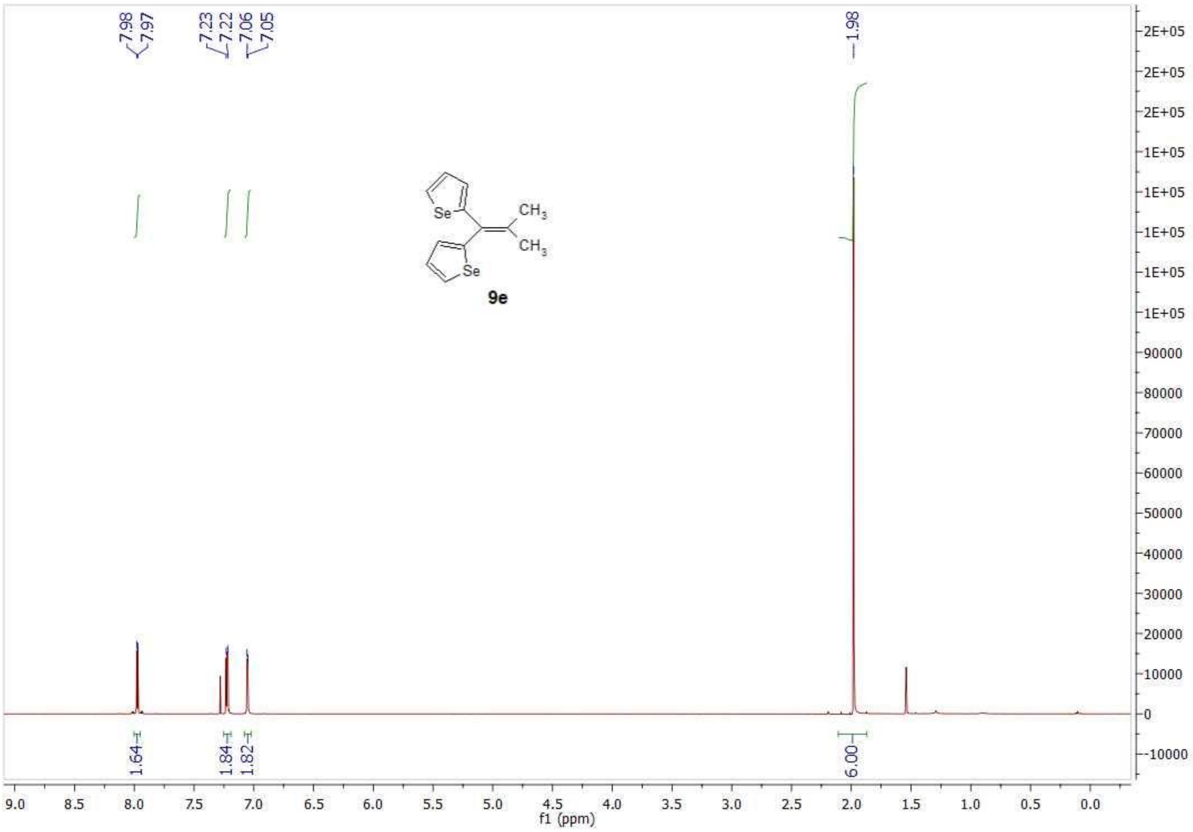

**Figure S11:** The $^{1}$H NMR spectrum of compound **9e**.

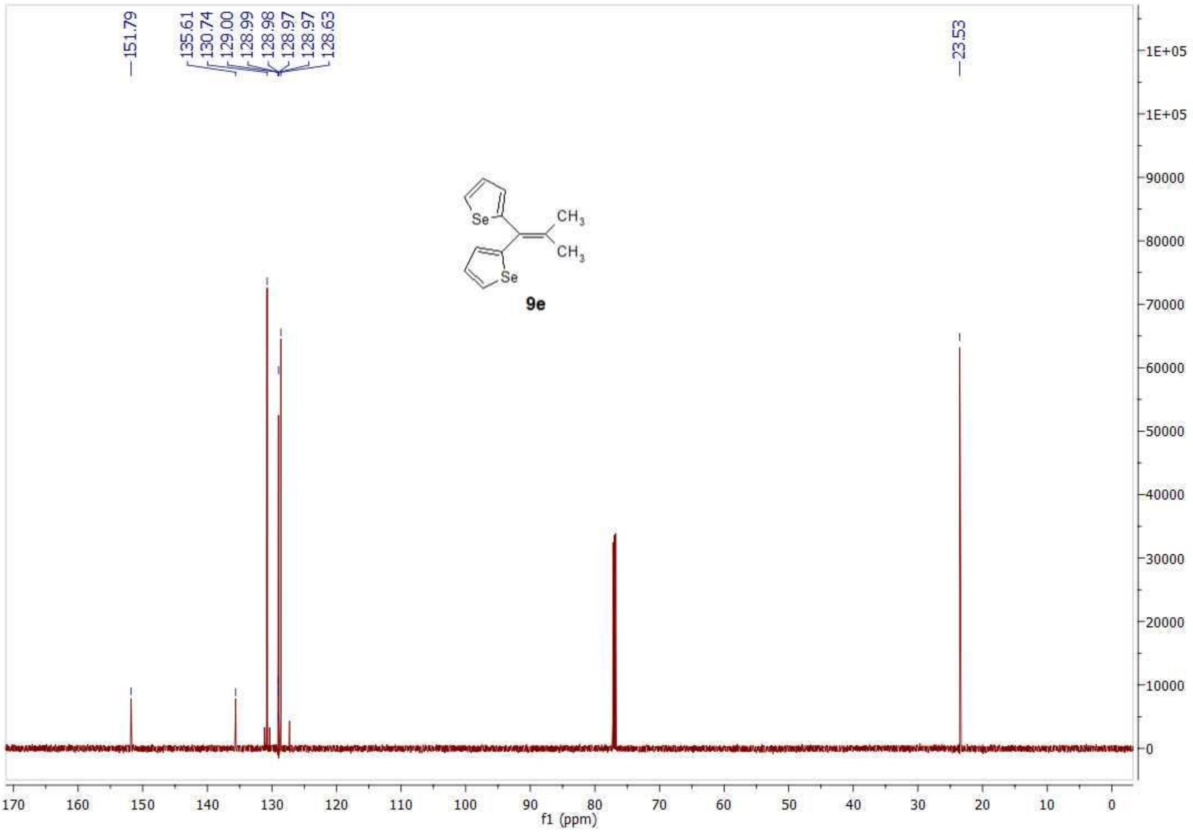


**Figure S12:** The $^{13}$C NMR spectrum of compound **9e**.

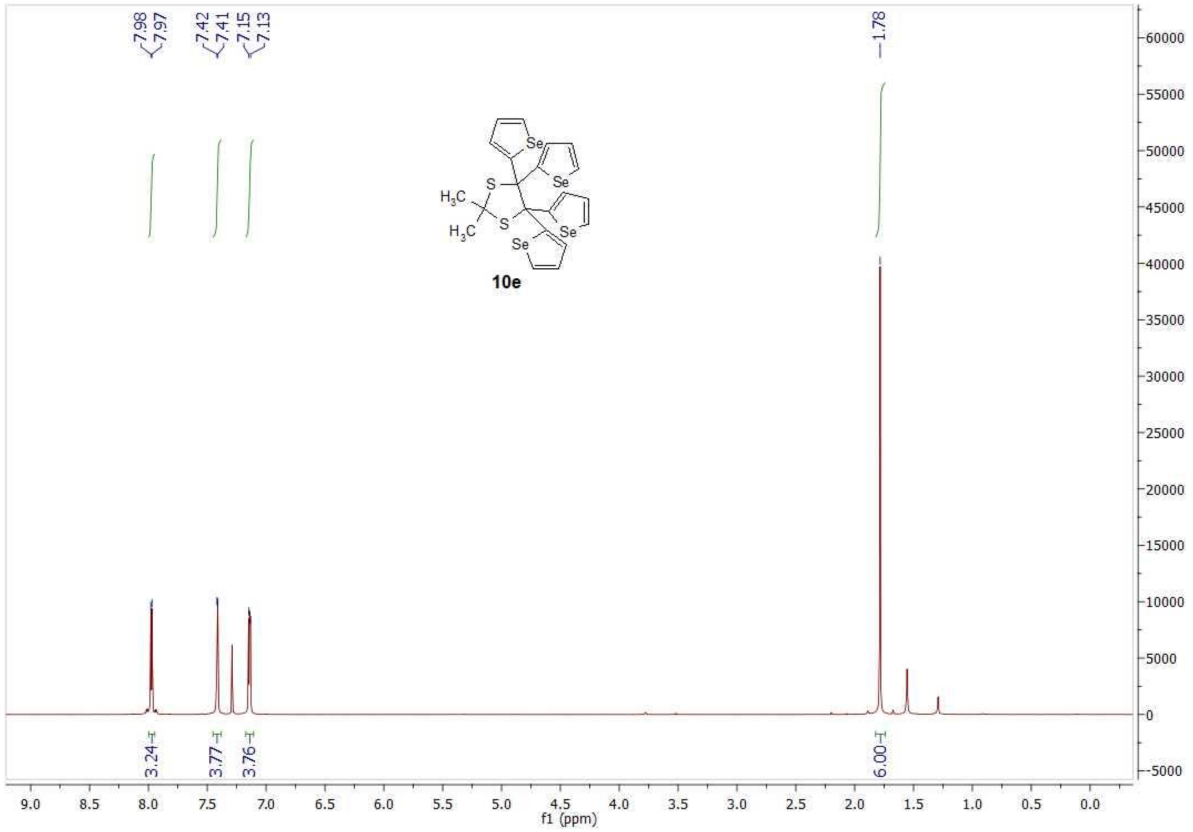

**Figure S13:** The $^{1}H$ NMR spectrum of compound **10e**.

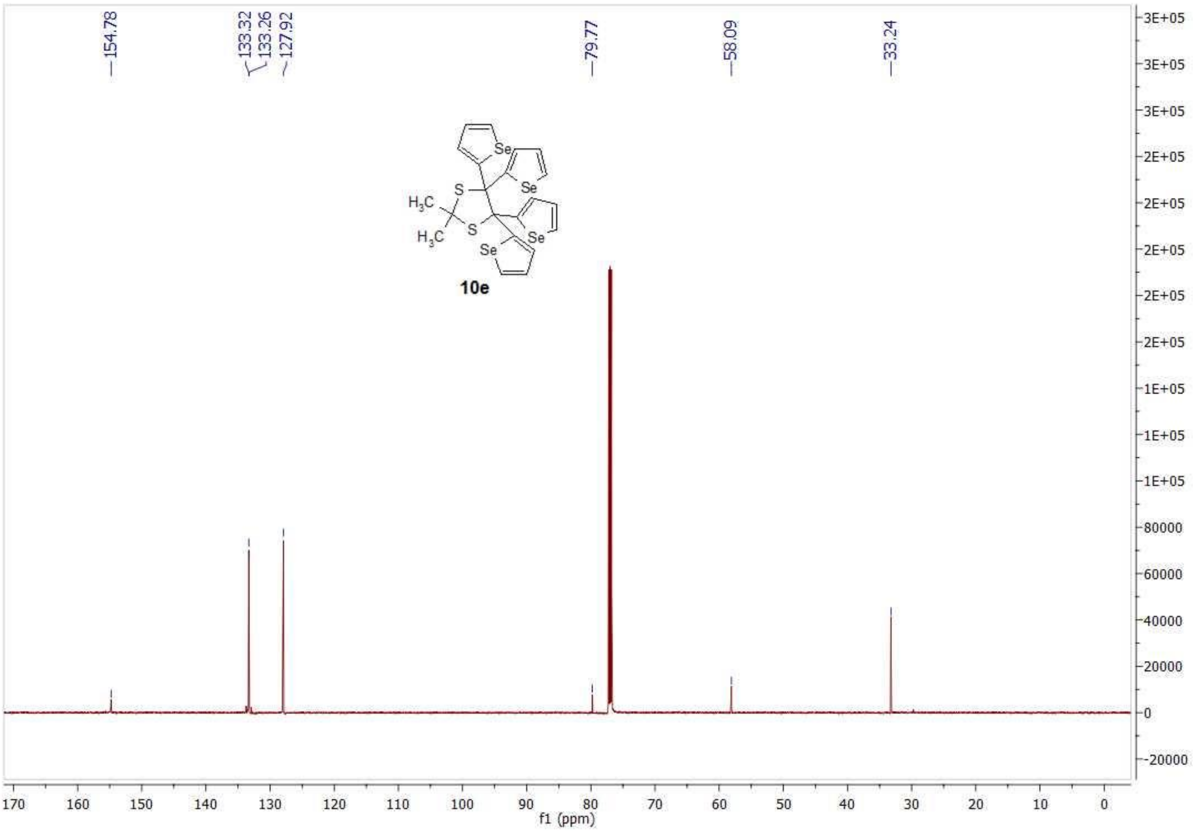


**Figure S14:** The $^{13}C$ NMR spectrum of compound **10e**.

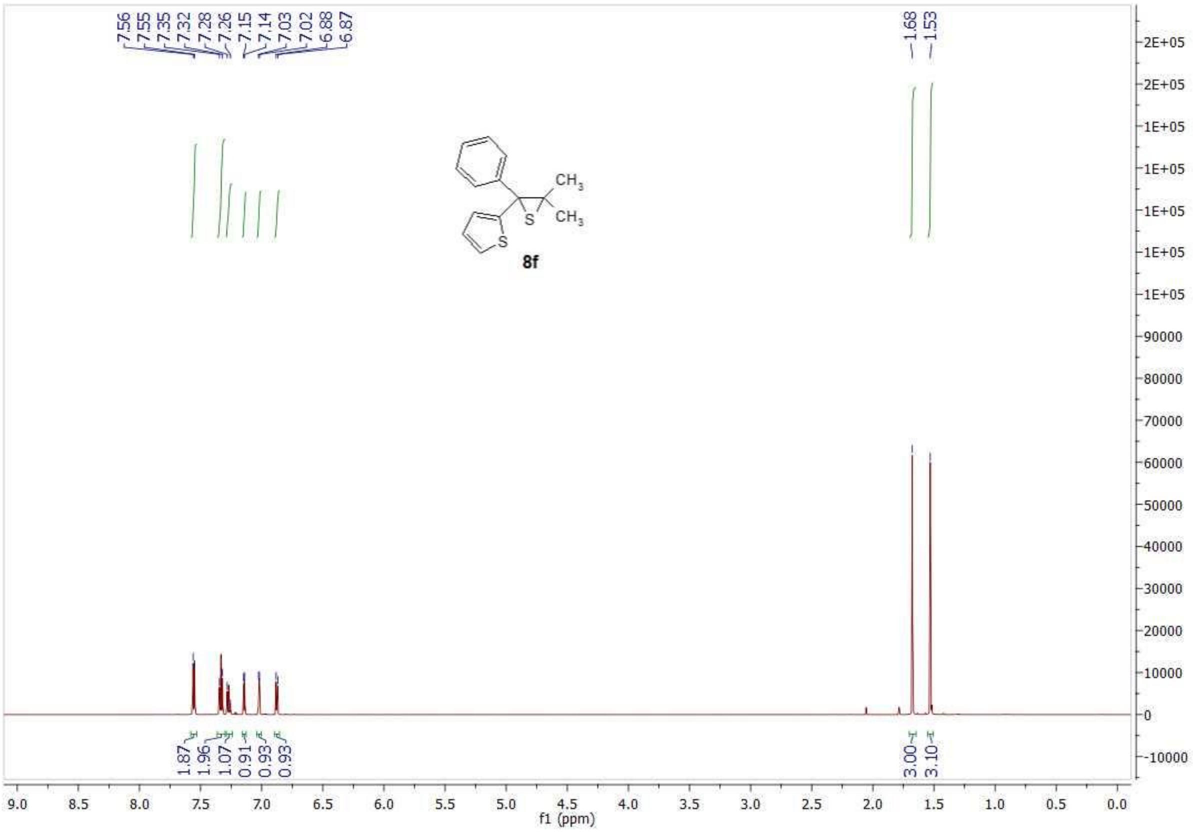

**Figure S15:** The $^{1}$H NMR spectrum of compound **8f**.

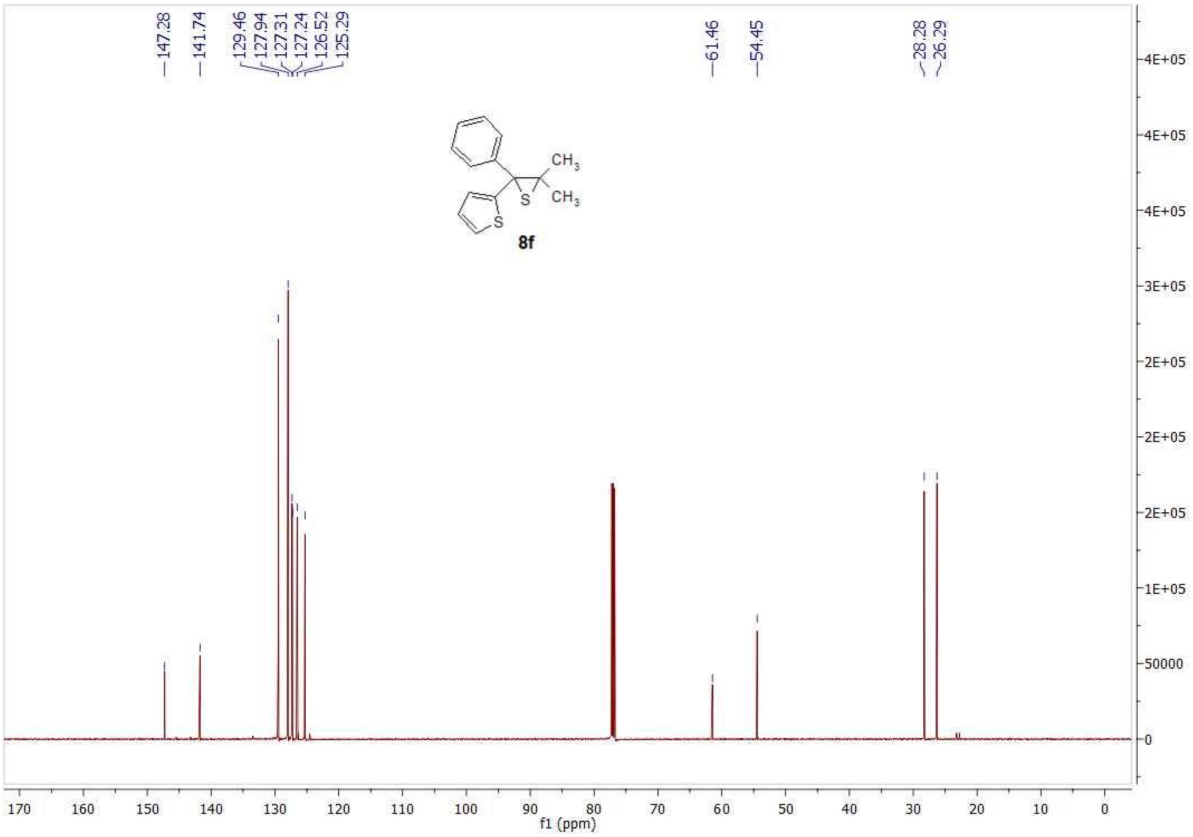


**Figure S16:** The $^{13}$C NMR spectrum of compound **8f**.

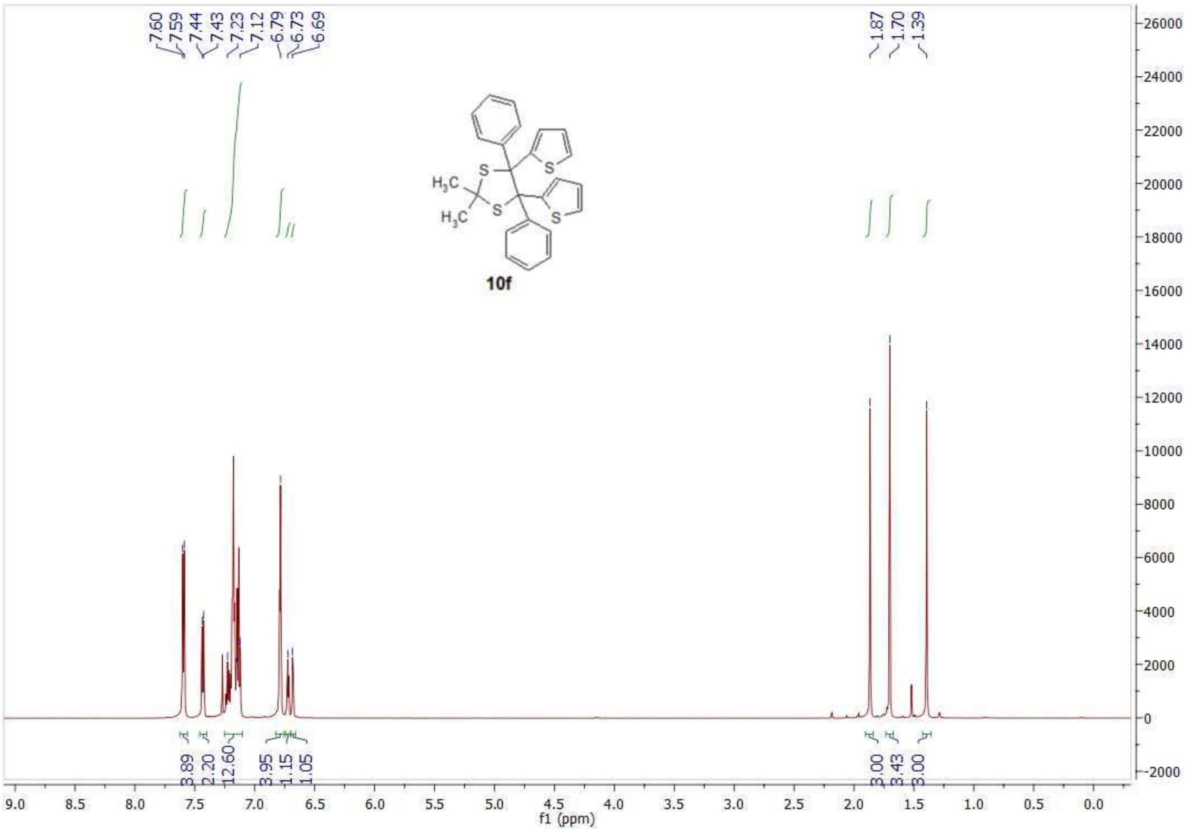

**Figure S17:** The $^{1}$H NMR spectrum of compound **10f**.

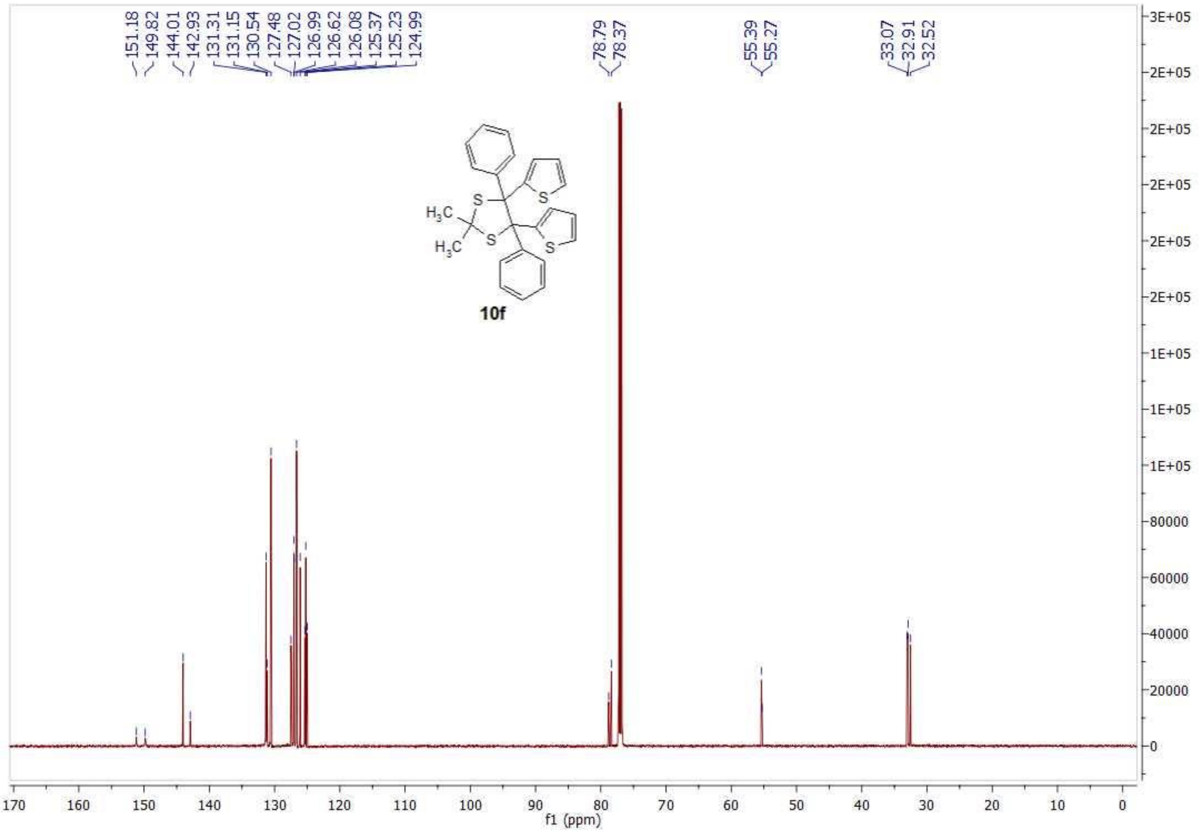


**Figure S18:** The $^{13}$C NMR spectrum of compound **10f**.

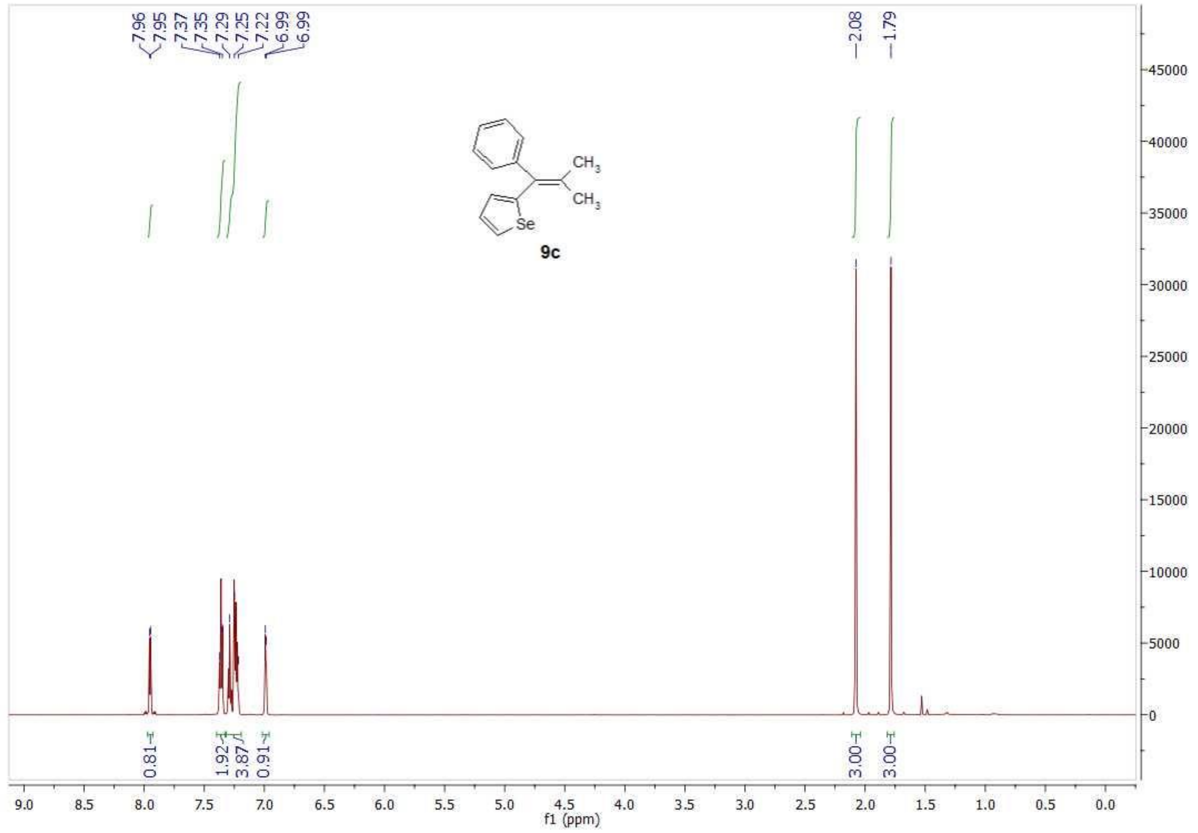

**Figure S19:** The $^{1}$H NMR spectrum of compound **9c**.

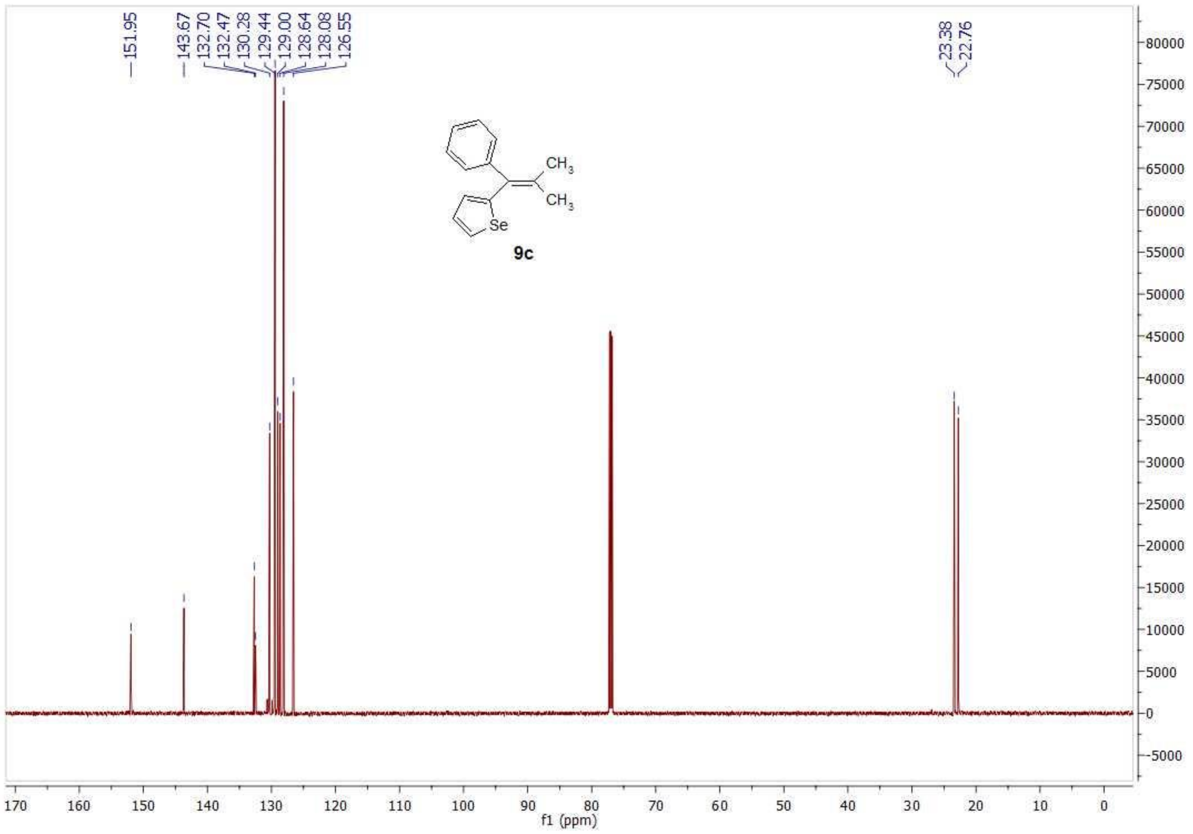


**Figure S20:** The $^{13}$C NMR spectrum of compound **9c**.

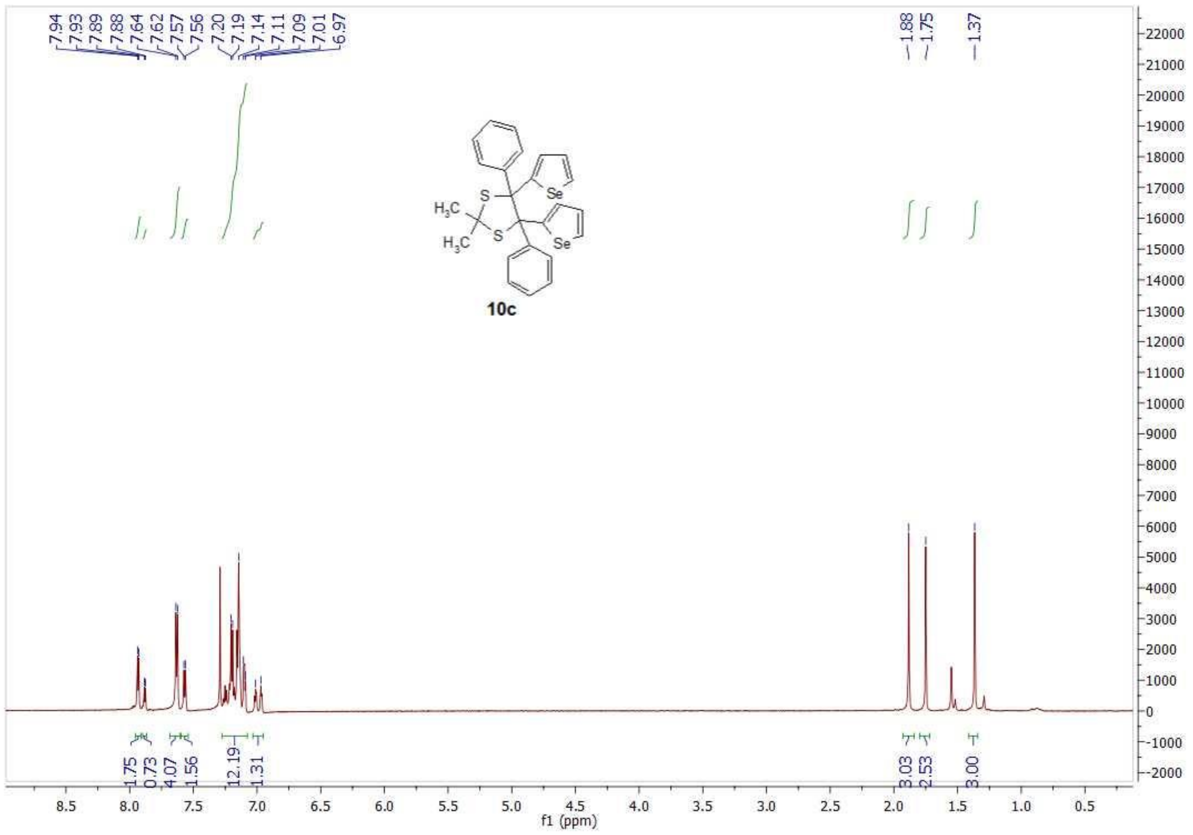


**Figure S21:** The $^{1}$H NMR spectrum of compound **10c**.

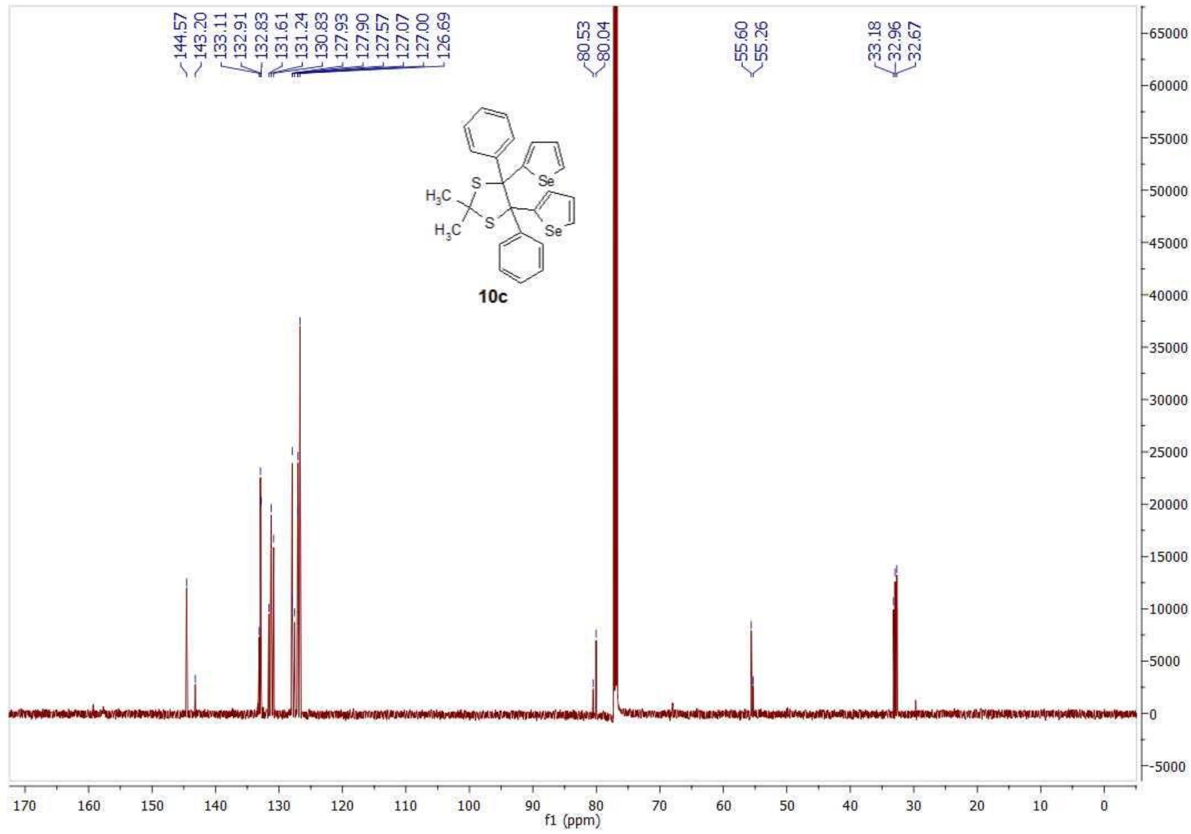


**Figure S22:** The $^{13}$C NMR spectrum of compound **10c**.

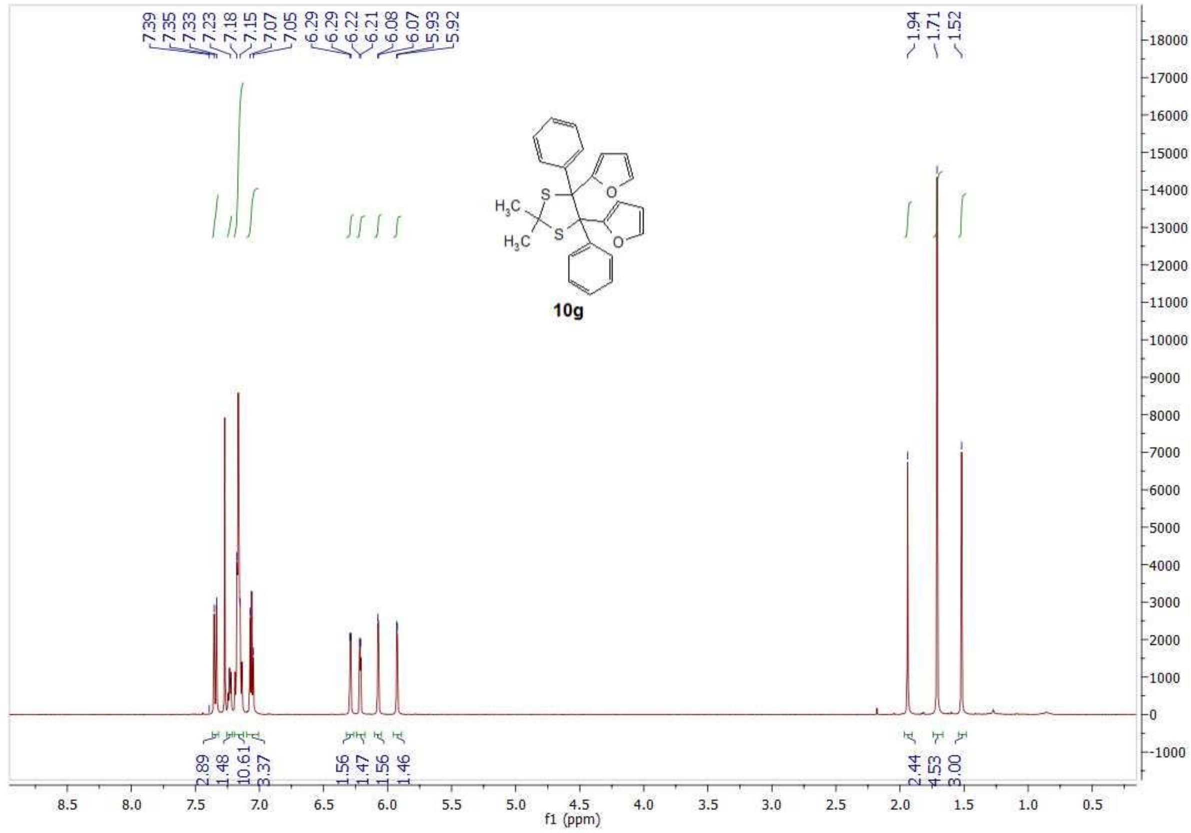


**Figure S23:** The $^{1}$H NMR spectrum of compound **10g**.

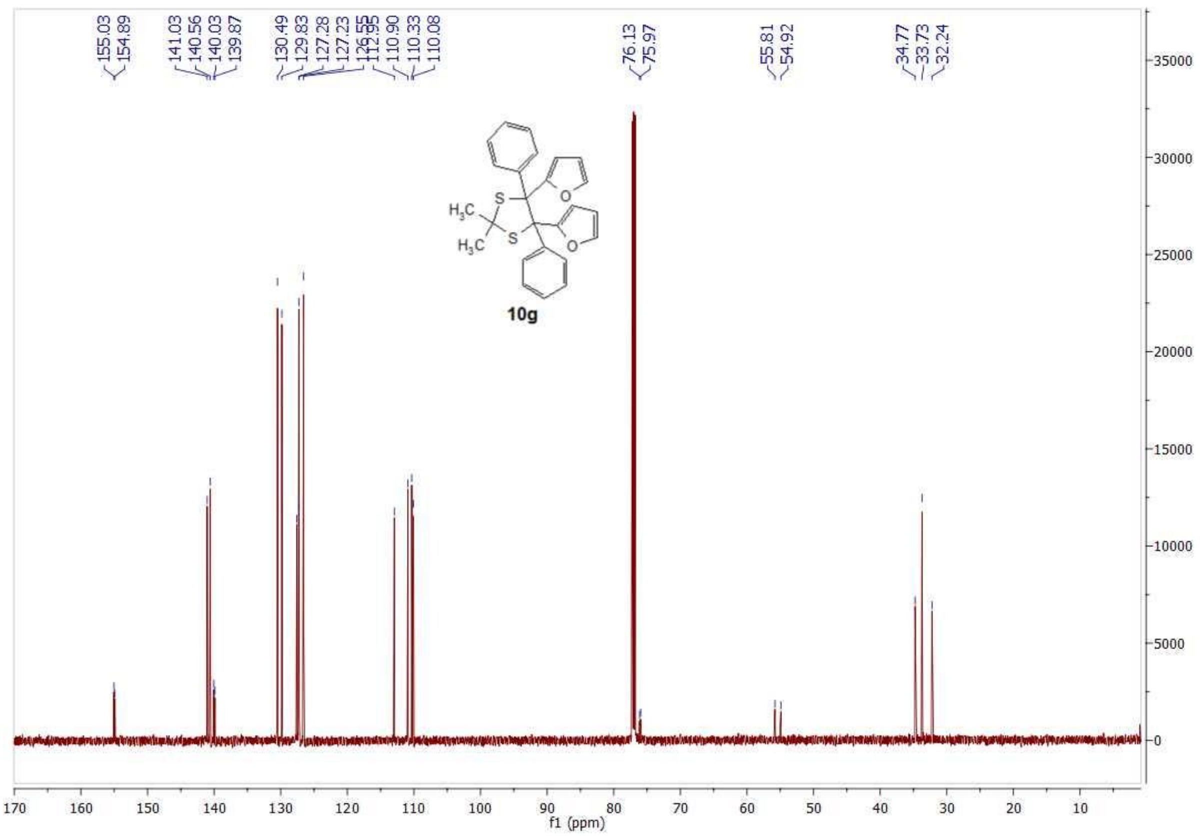

**Figure S24:** The $^{13}C$ NMR spectrum of compound **10g**.

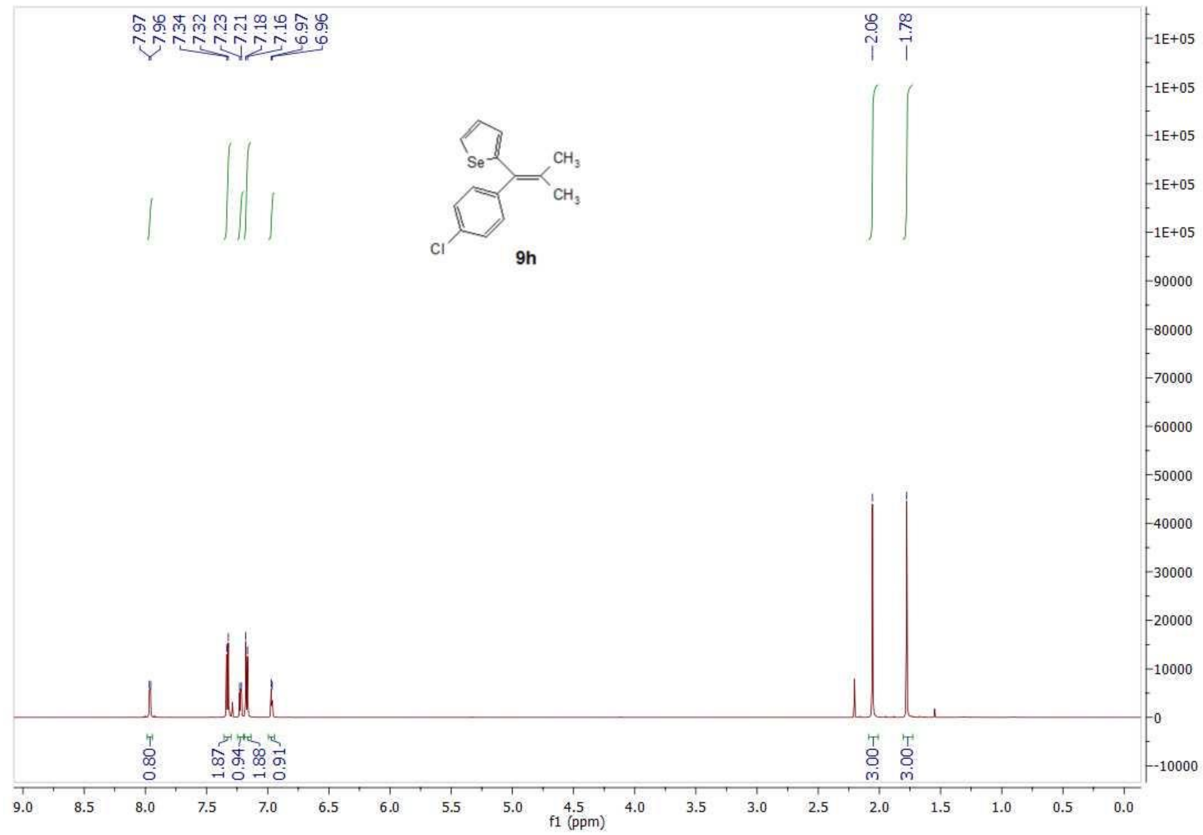

**Figure S25:** The $^{1}H$ NMR spectrum of compound **9h**.

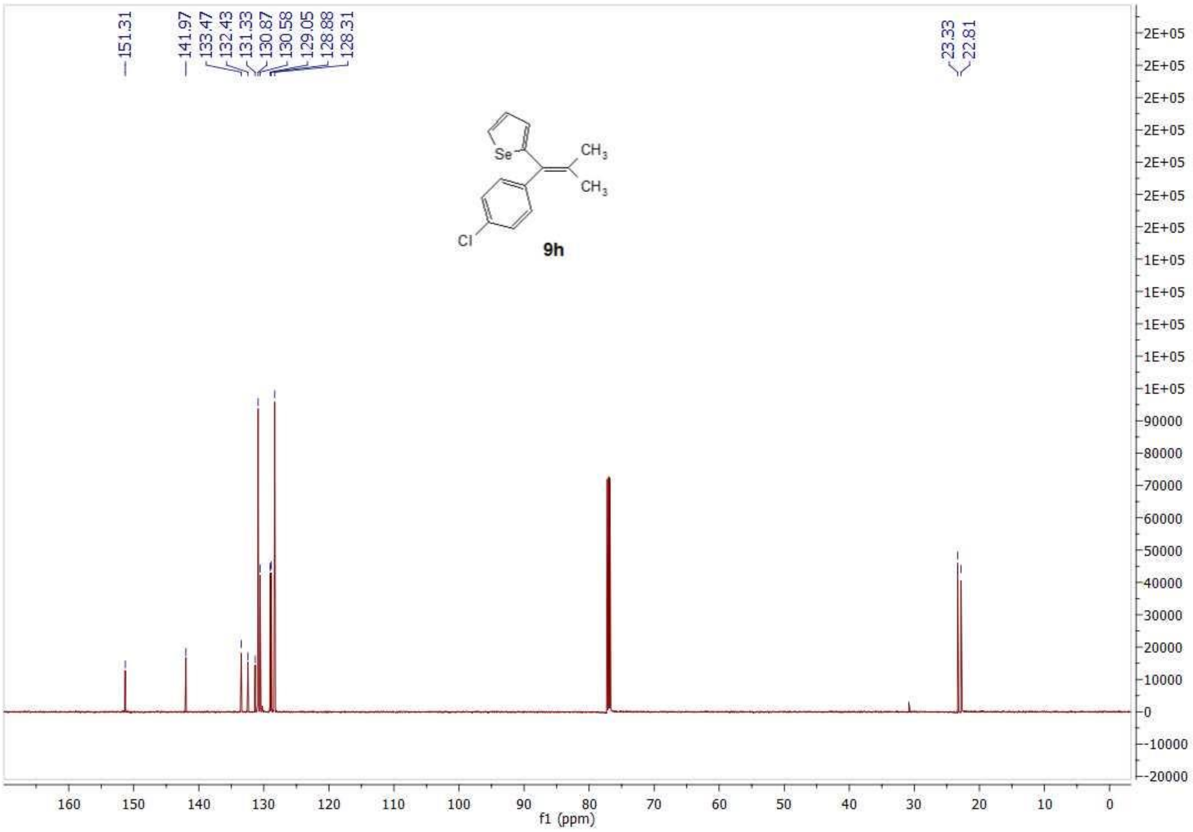


**Figure S26:** The $^{13}$C NMR spectrum of compound **9h**.

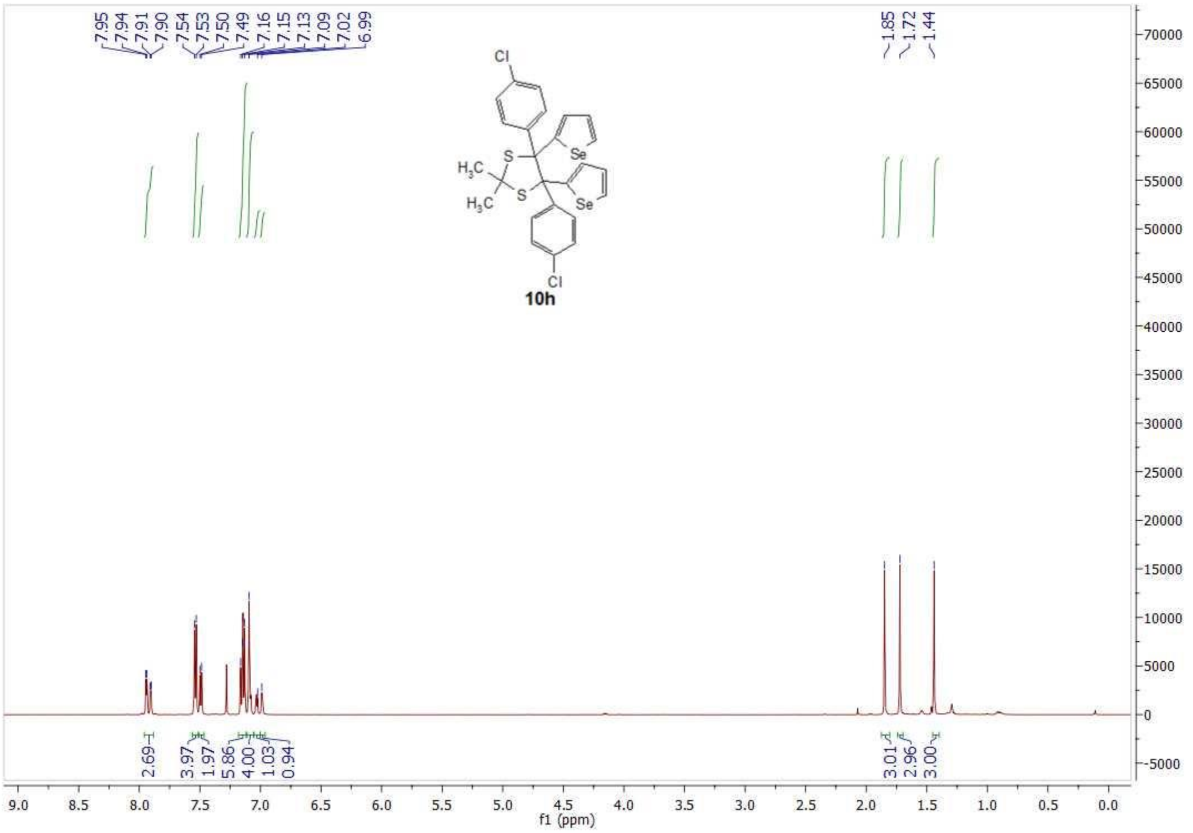


**Figure S27:** The $^{1}$H NMR spectrum of compound **10h**.

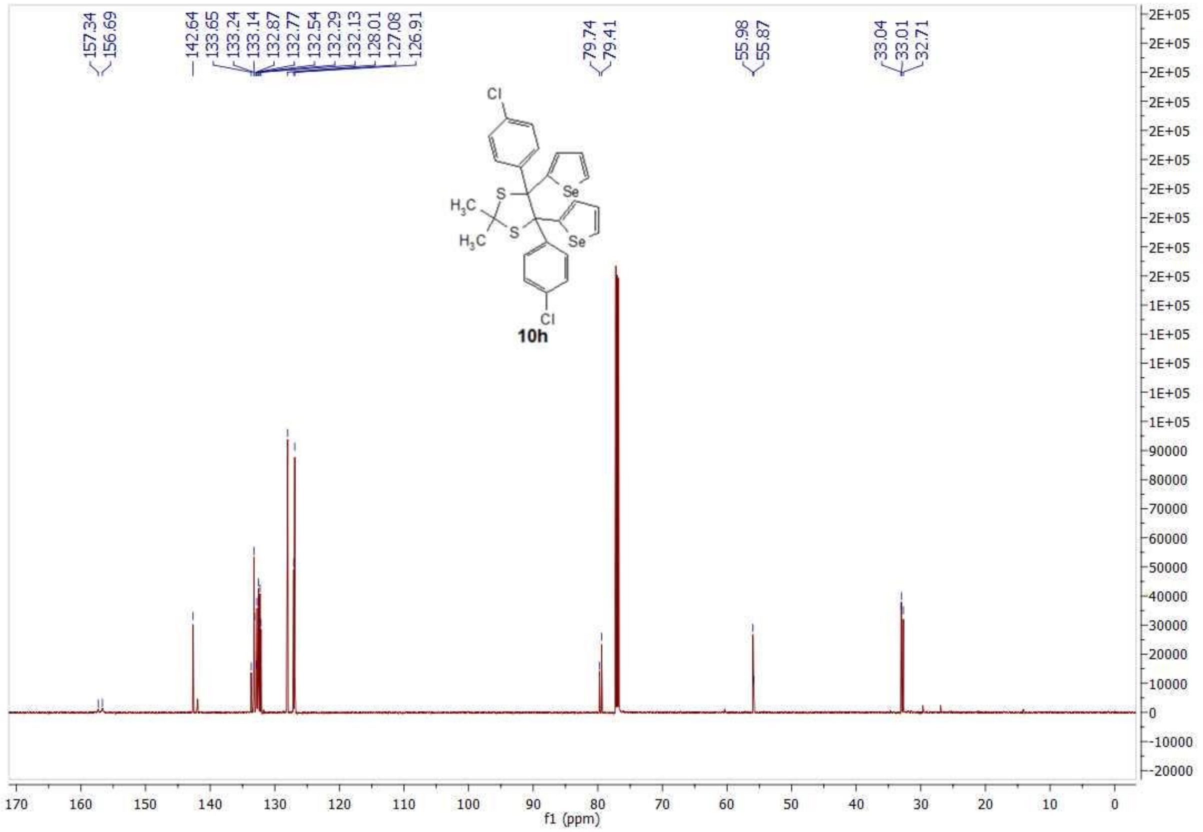


**Figure S28:** The $^{13}$C NMR spectrum of compound **10h**.

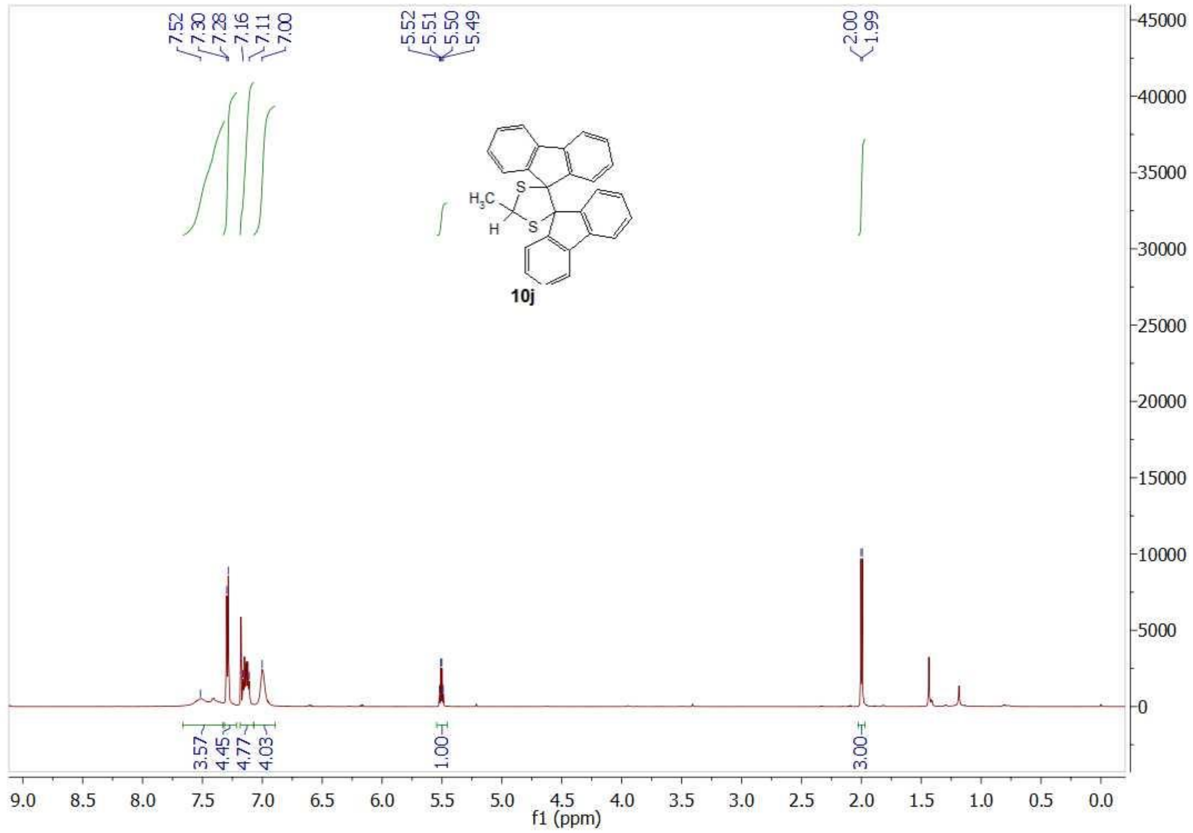


**Figure S29:** The $^{1}$H NMR spectrum of compound **10j**.

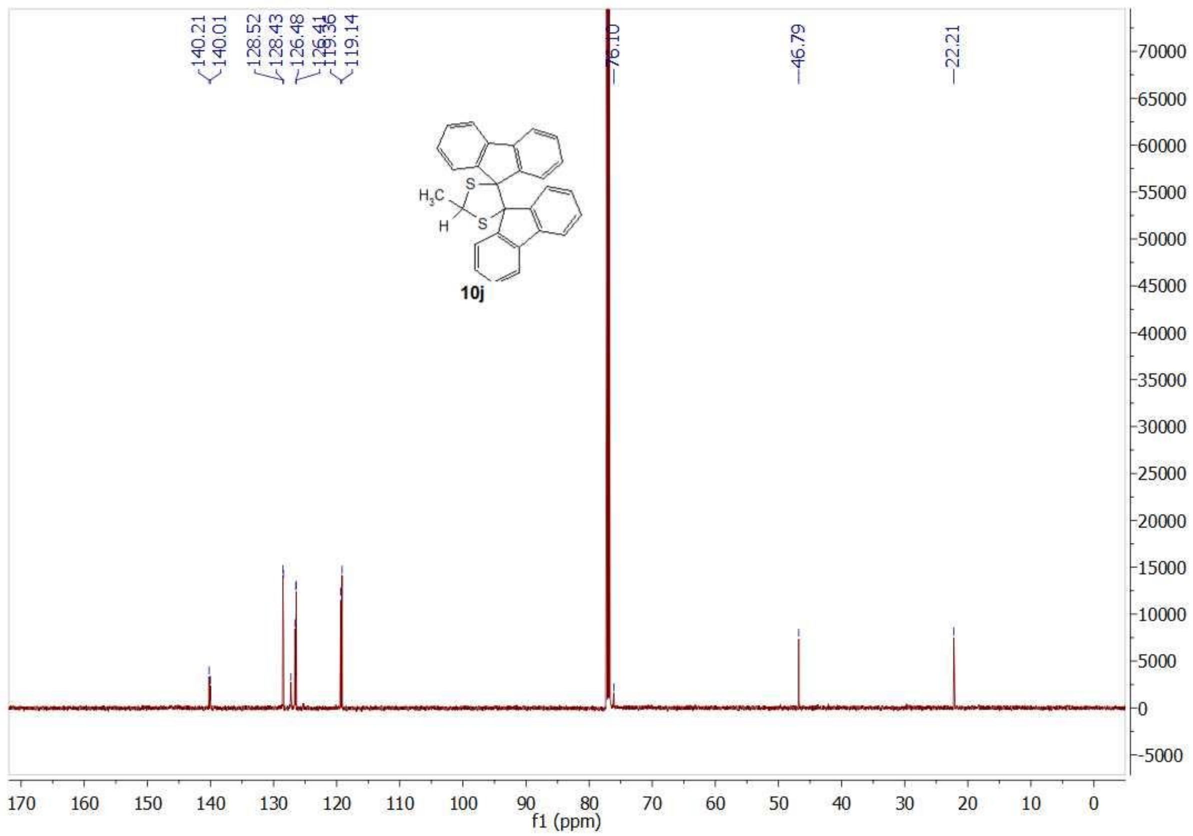


**Figure S30:** The $^{13}$C NMR spectrum of compound **10j**.

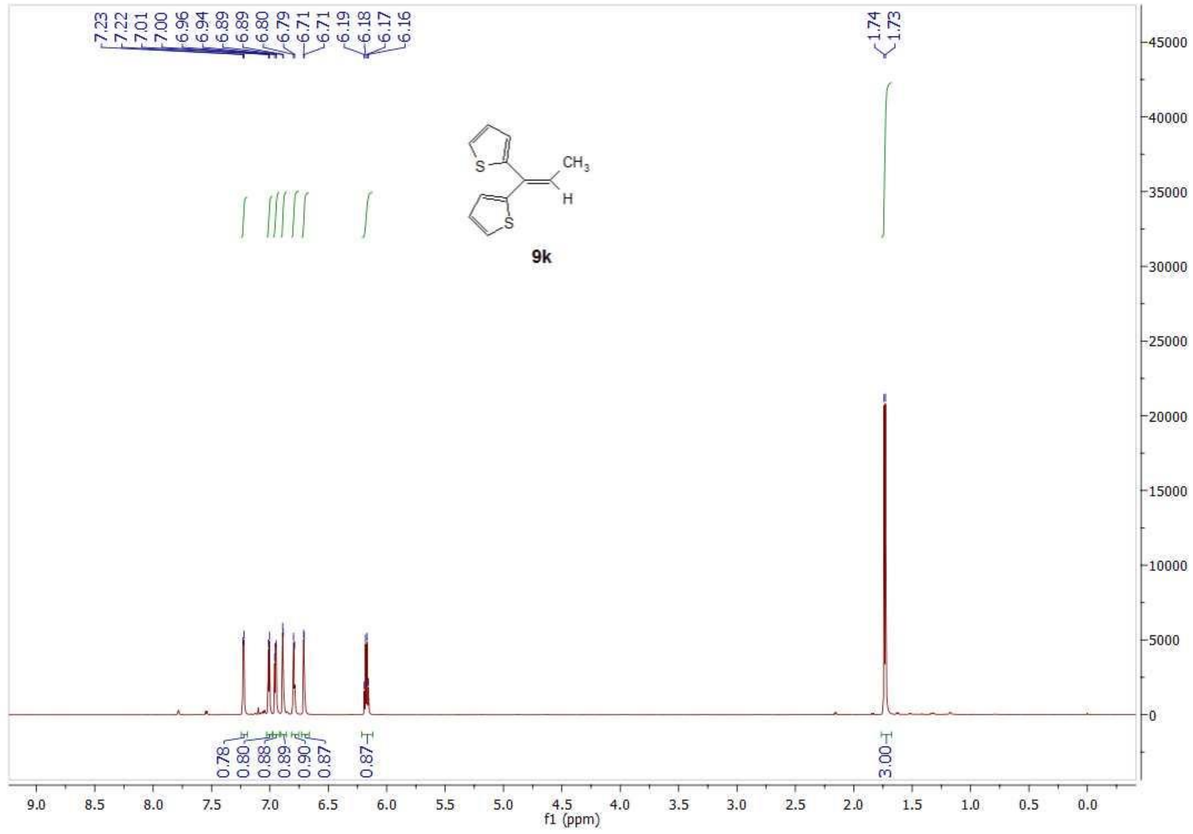


**Figure S31:** The $^{1}$H NMR spectrum of compound **9k**.

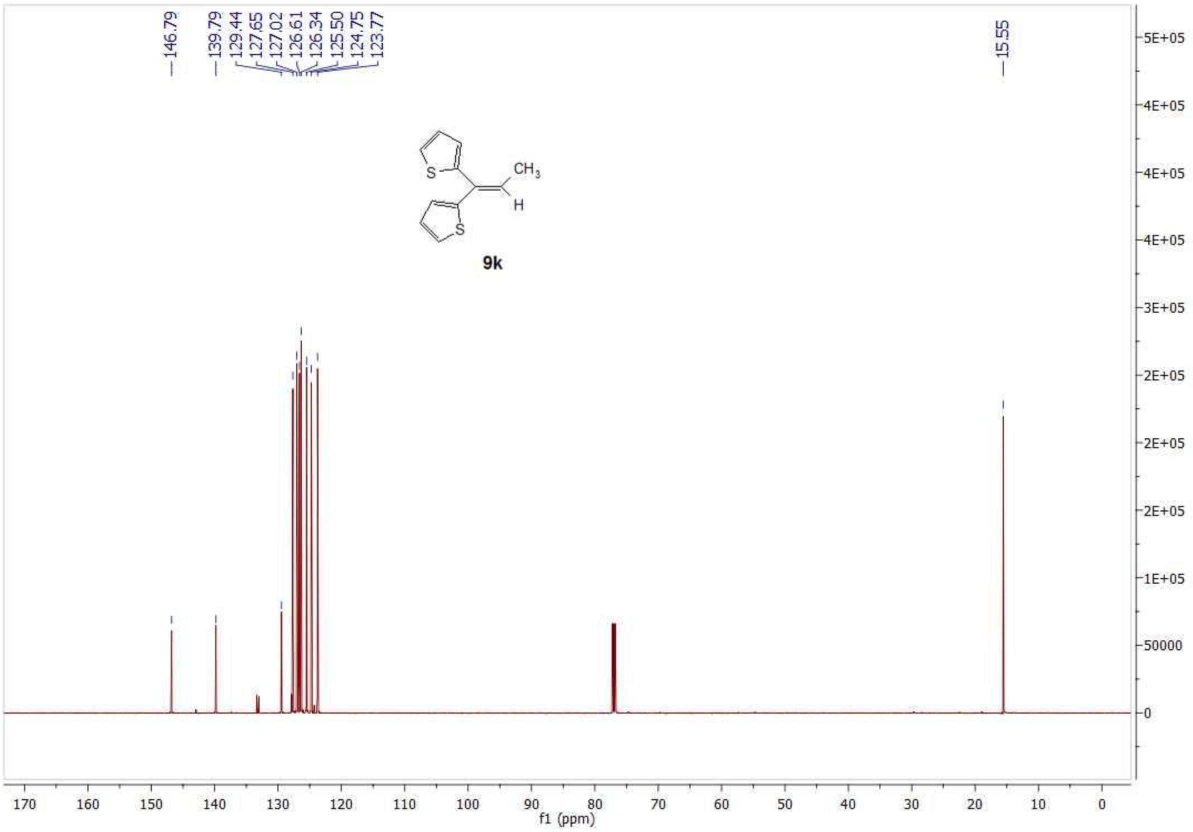


**Figure S32:** The $^{13}$C NMR spectrum of compound **9k**.

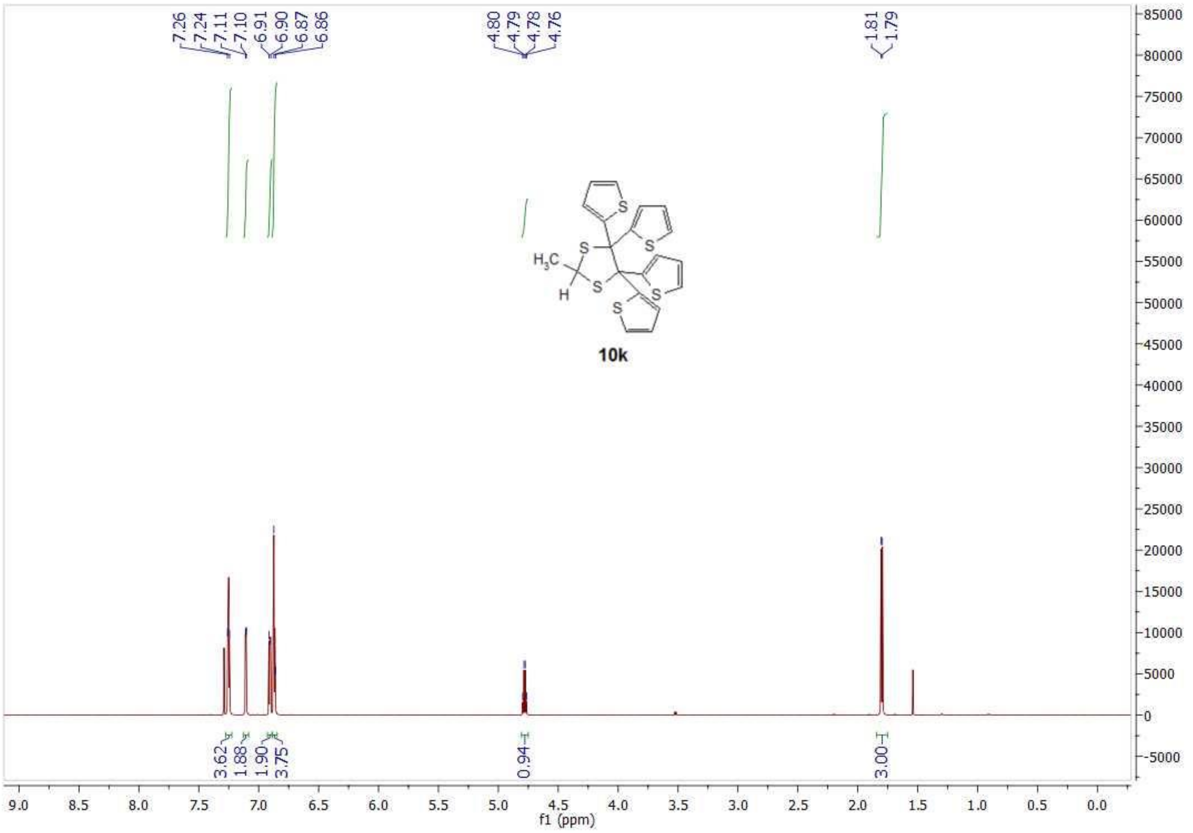


**Figure S33:** The $^{1}$H NMR spectrum of compound **10k**.

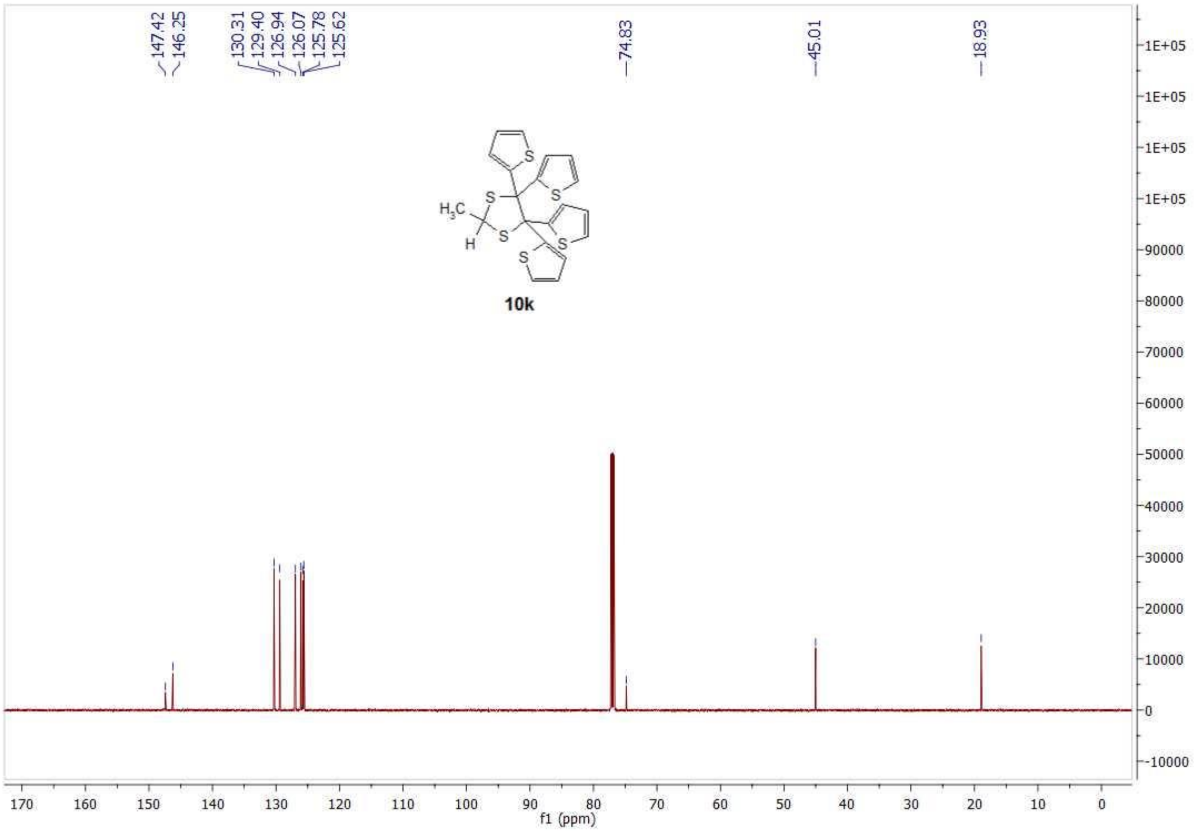


**Figure S34:** The $^{13}C$ NMR spectrum of compound **10k**.

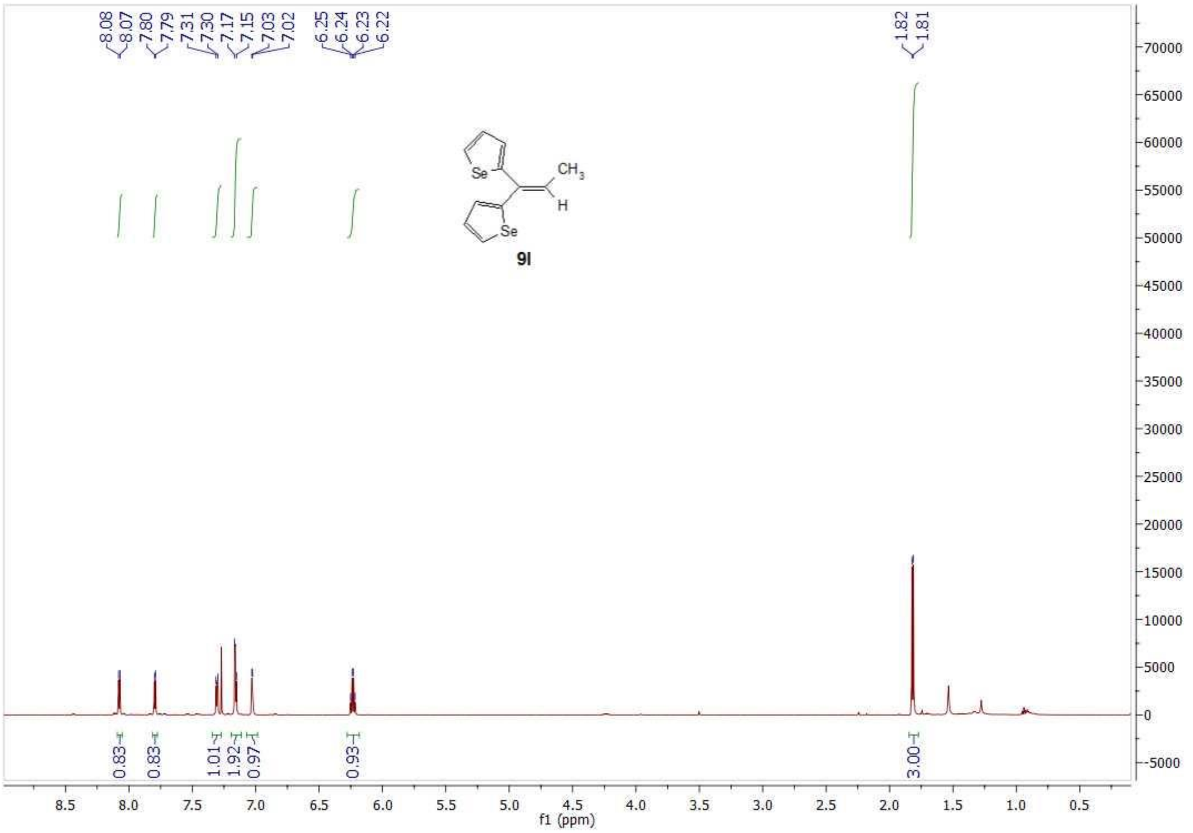


**Figure S35:** The $^{1}H$ NMR spectrum of compound **9l**.

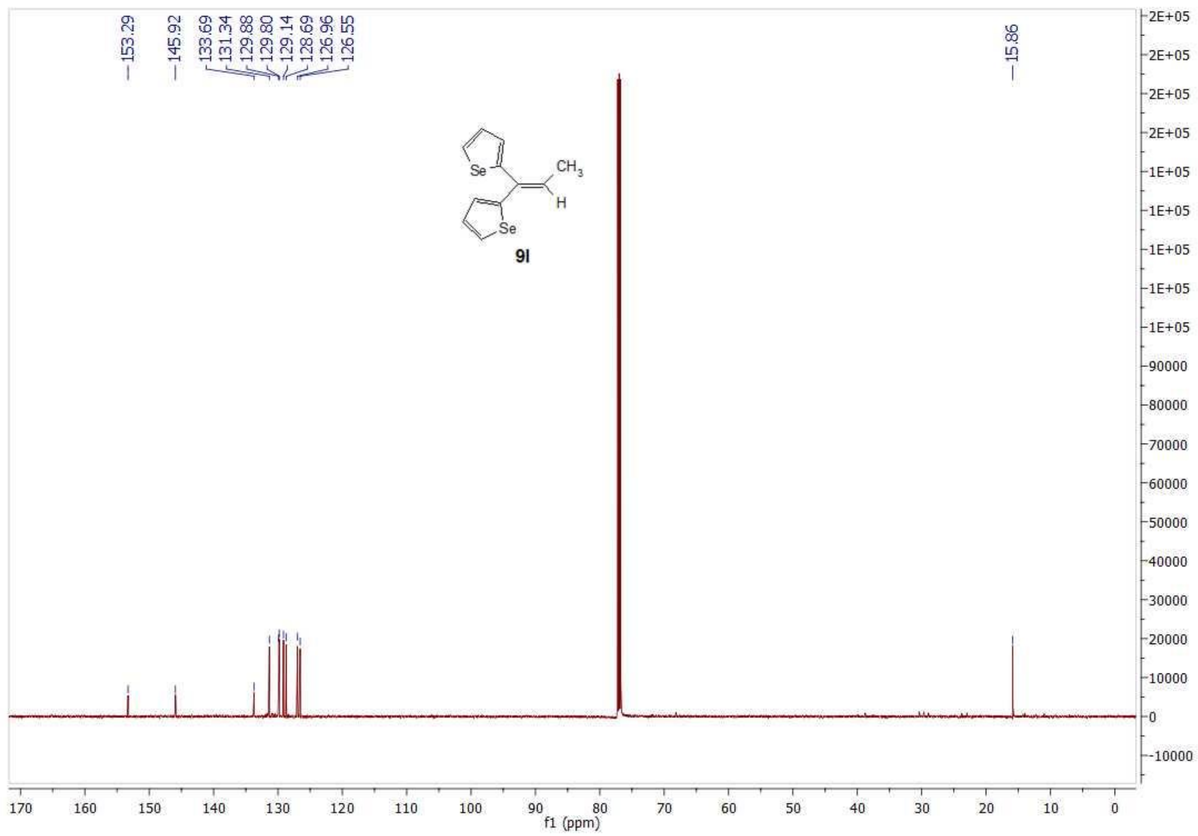


**Figure S36:** The $^{13}C$ NMR spectrum of compound **9l**.

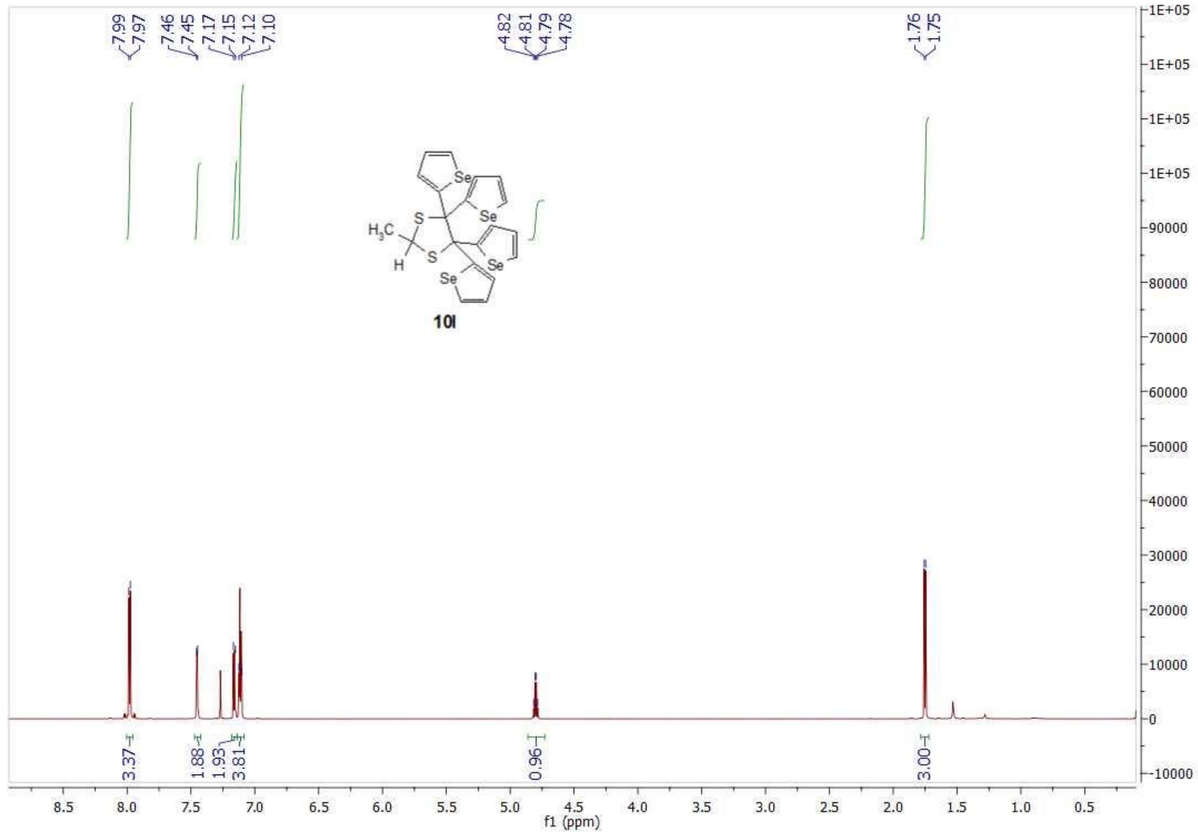


**Figure S37:** The $^{1}H$ NMR spectrum of compound **10l**.

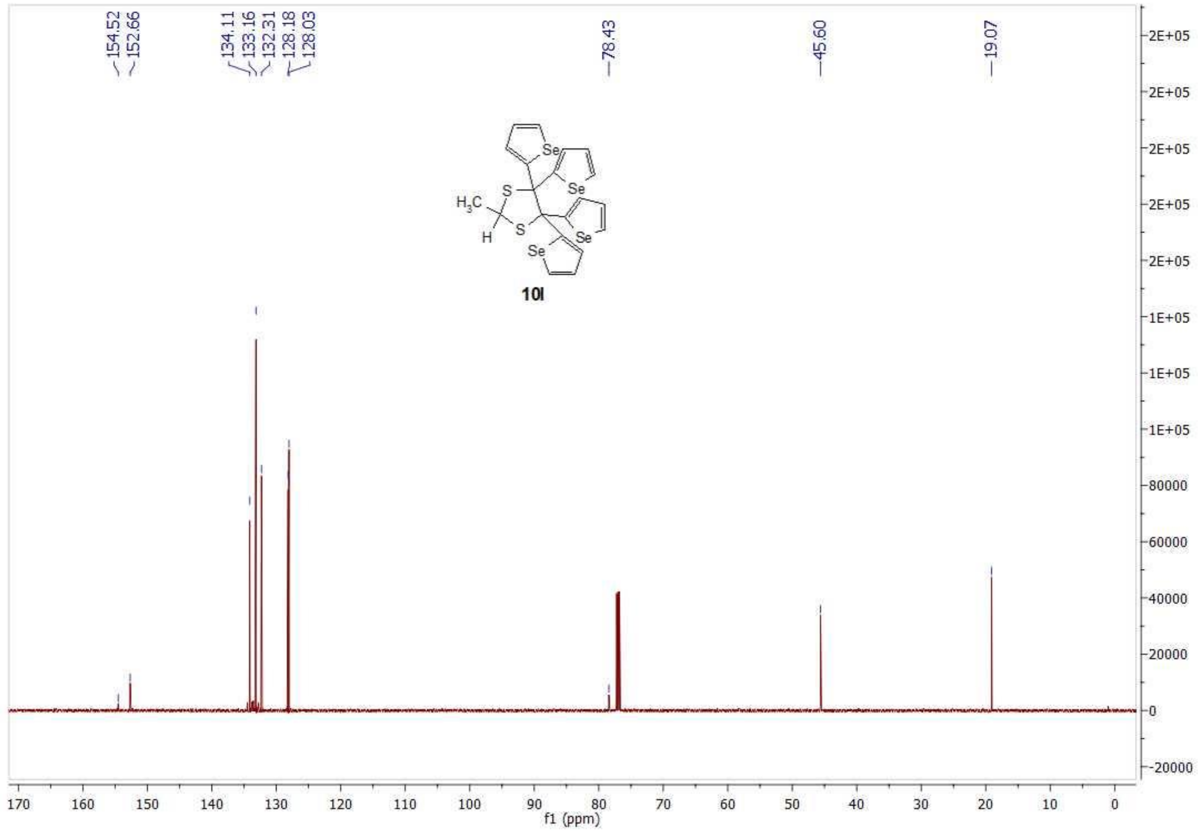


**Figure S38:** The $^{13}C$ NMR spectrum of compound **10l**.

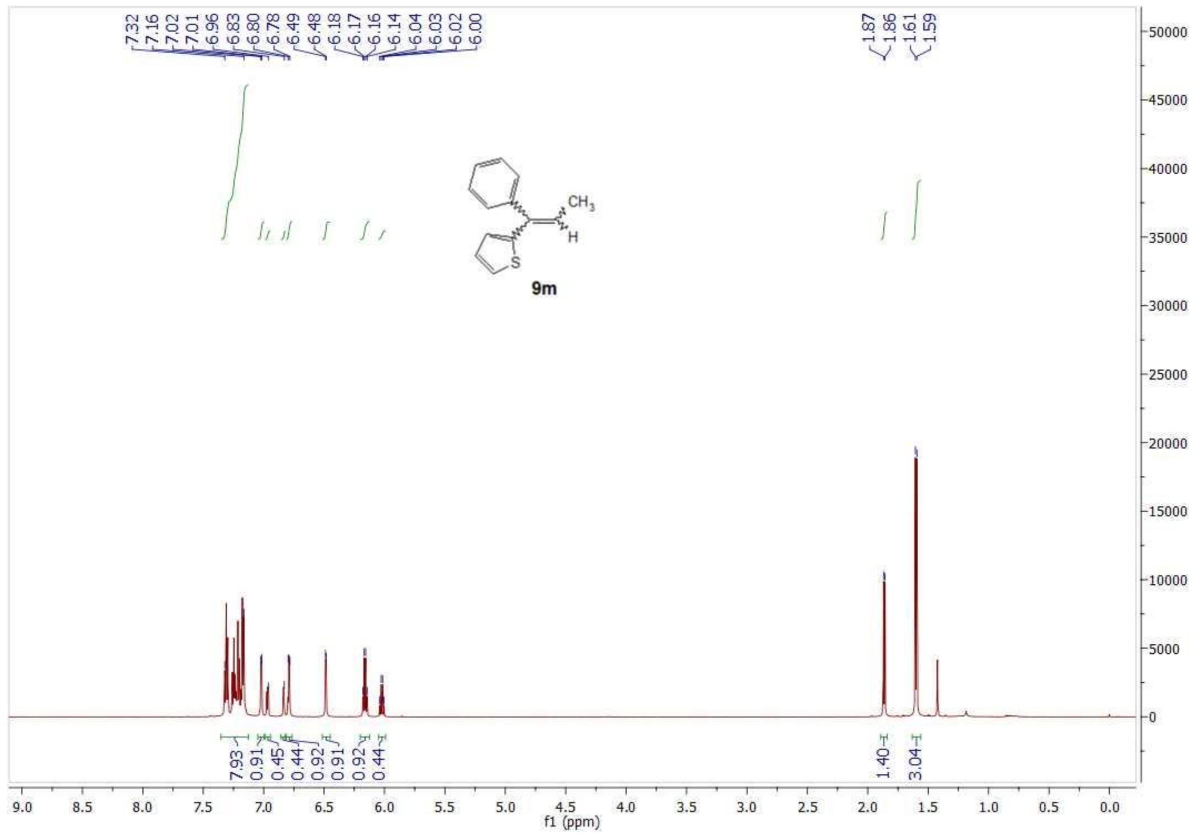


**Figure S39:** The $^{1}H$ NMR spectrum of compound **9m**.

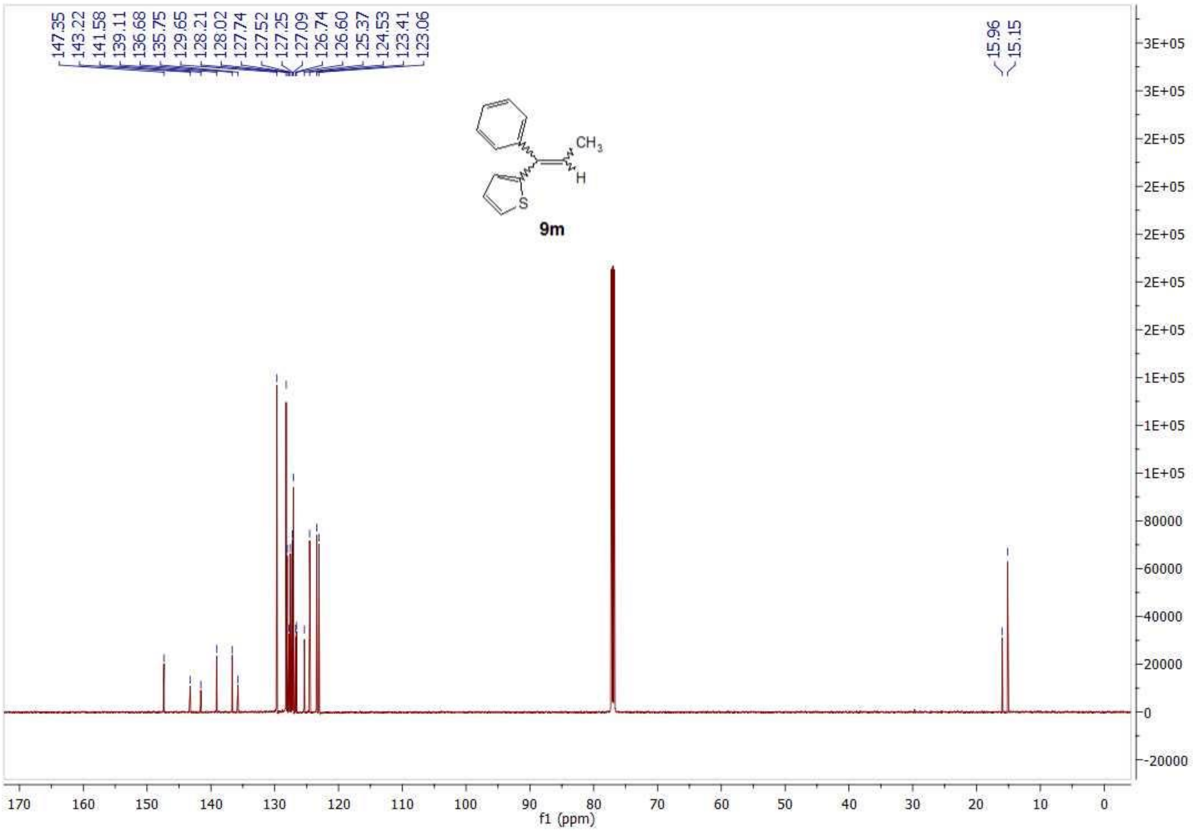

**Figure S40:** The $^{13}C$ NMR spectrum of compound **9m**.

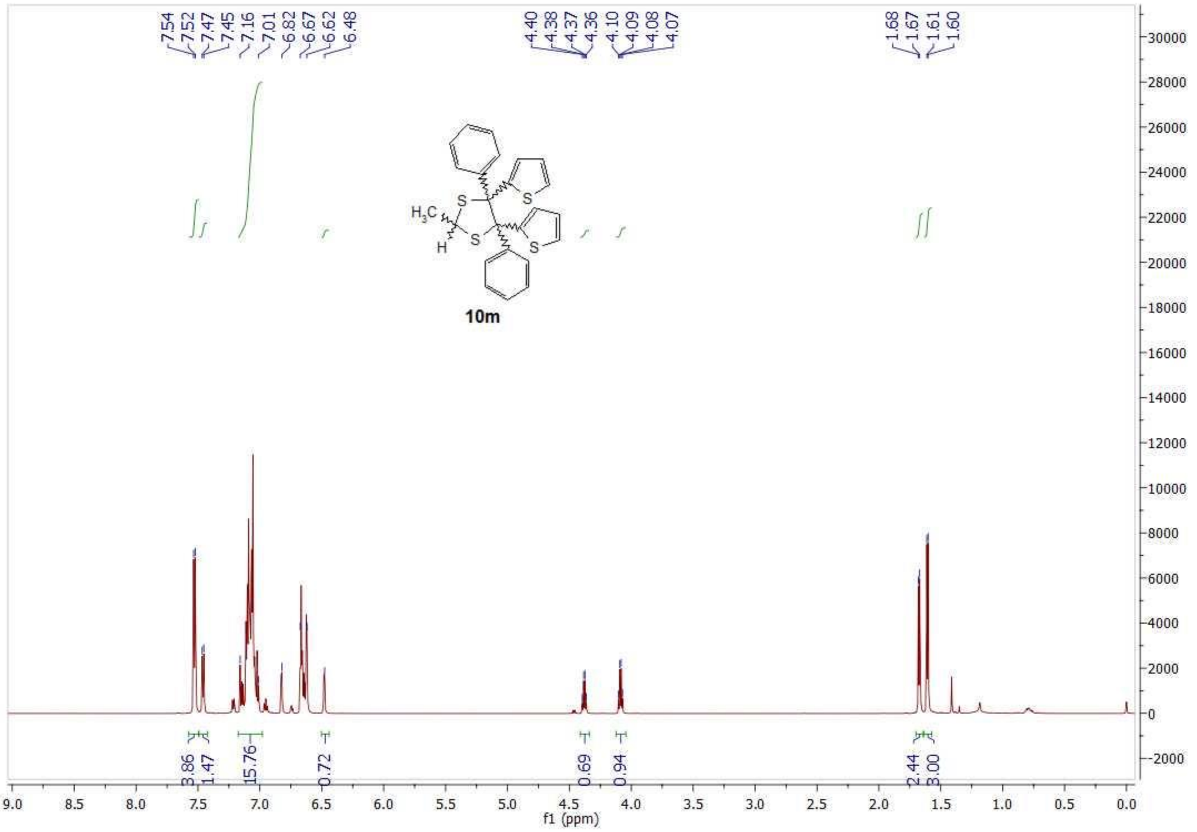

**Figure S41:** The $^{1}H$ NMR spectrum of compound **10m**.

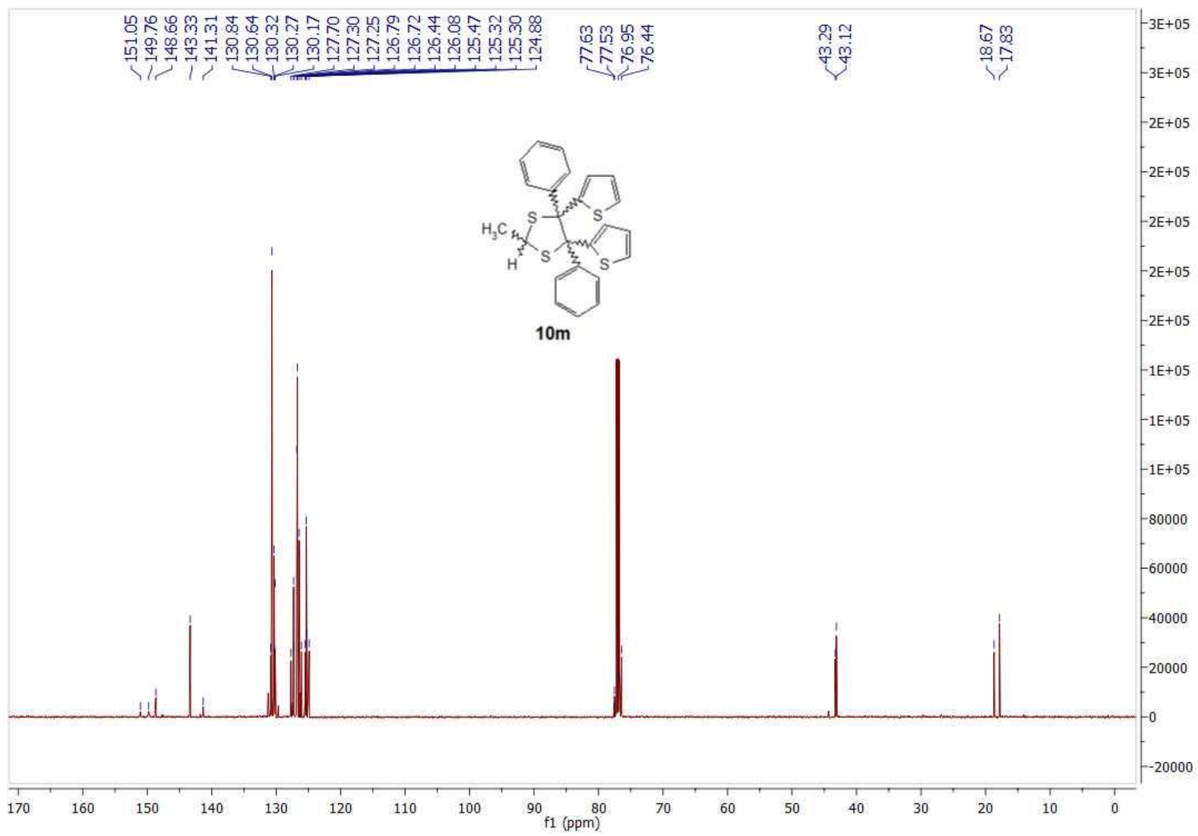


**Figure S42:** The $^{13}C$ NMR spectrum of compound **10m**.

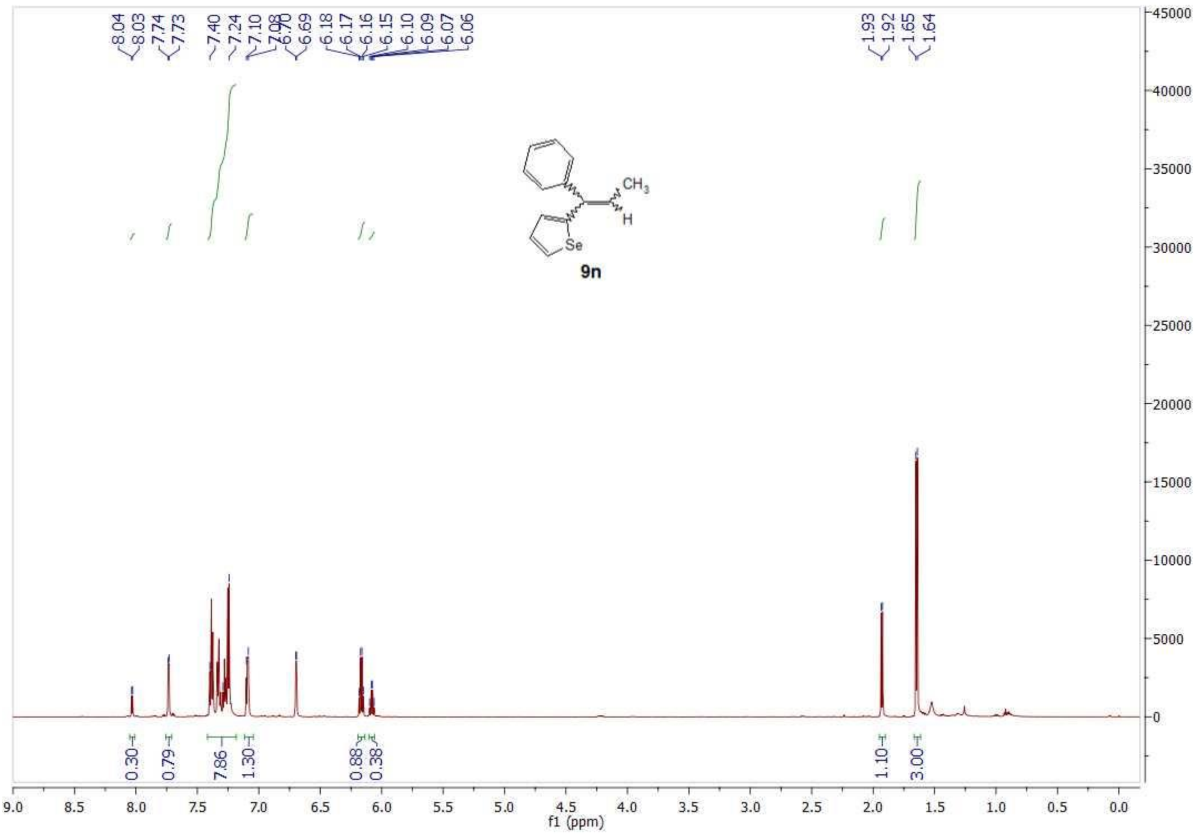


**Figure S43:** The $^{1}H$ NMR spectrum of compound **9n**.

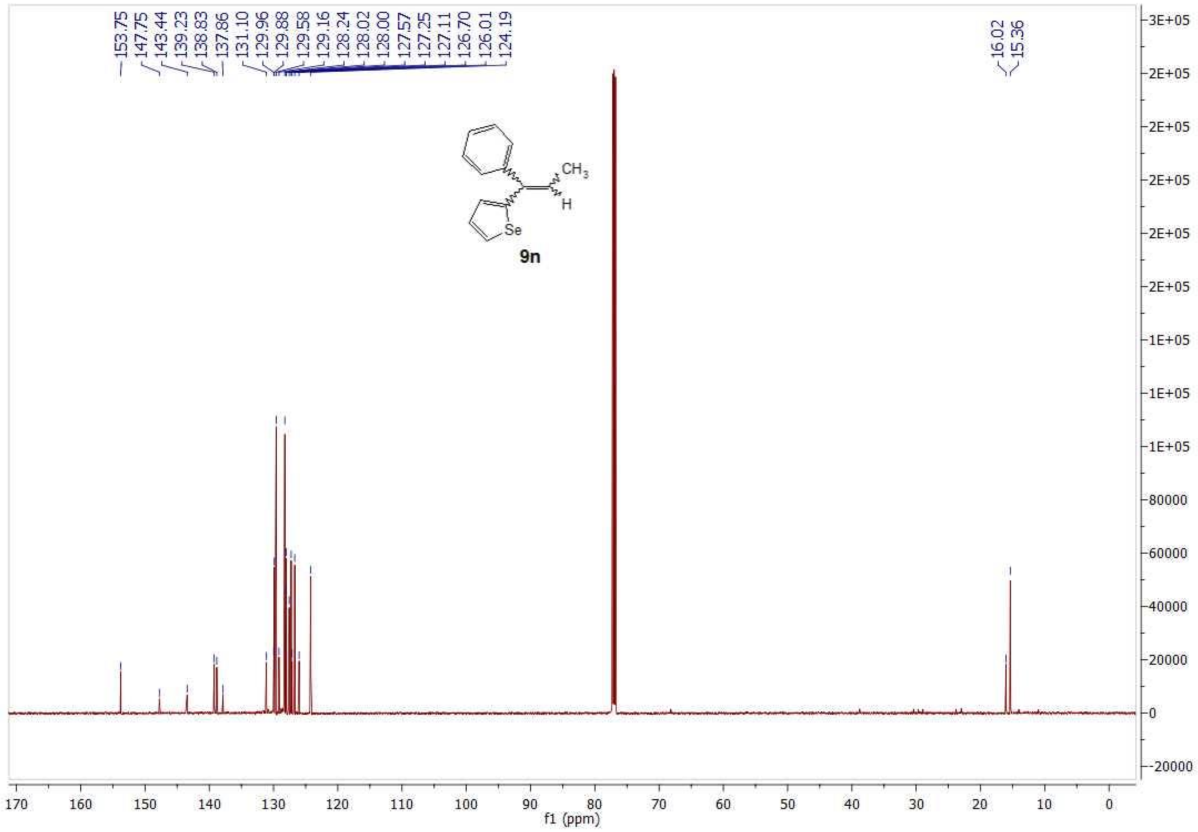

**Figure S44:** The $^{13}C$ NMR spectrum of compound **9n**.

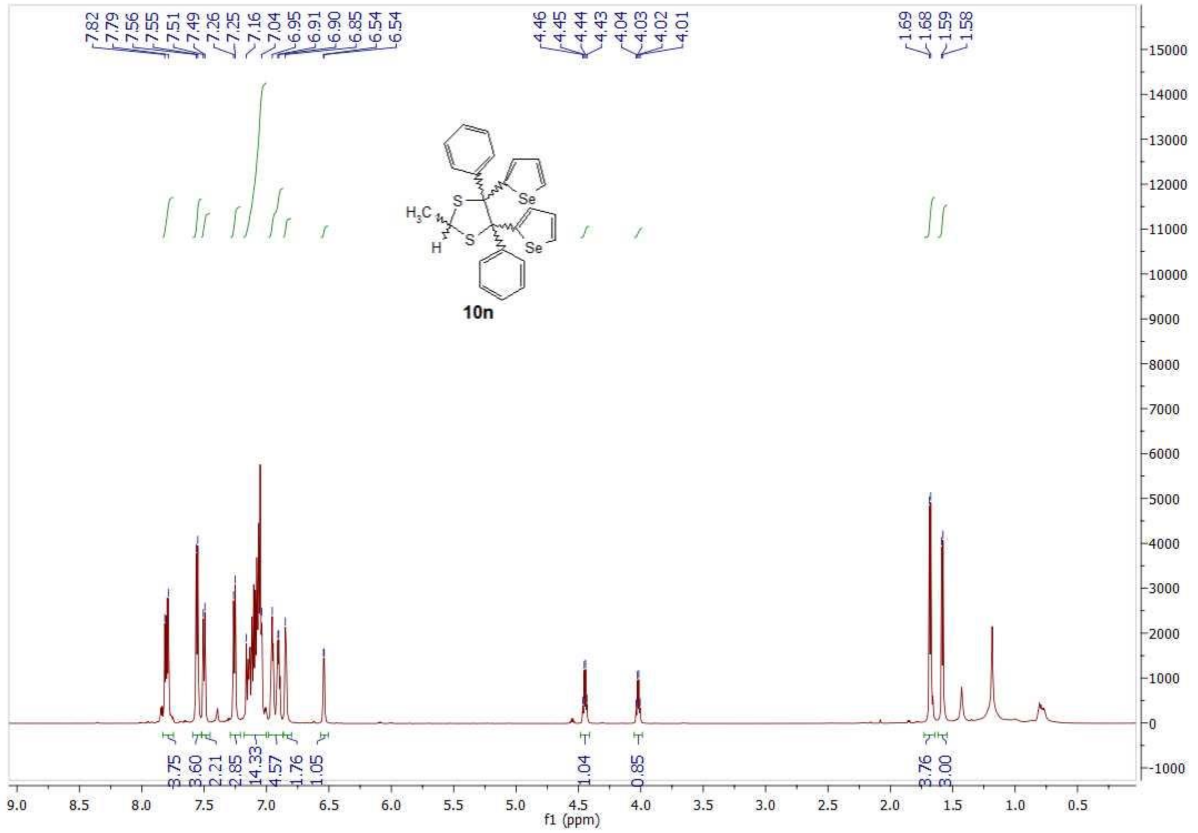

**Figure S45:** The $^{1}H$ NMR spectrum of compound **10n**.

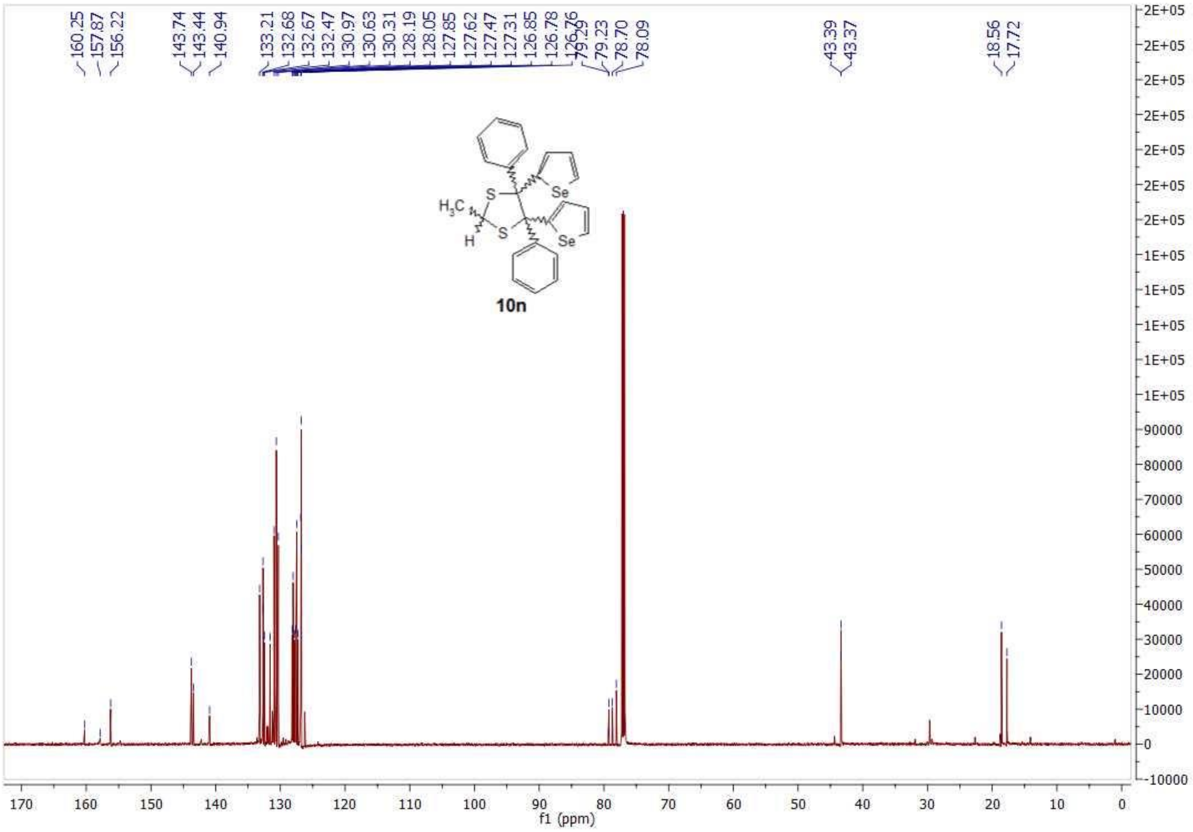


**Figure S46:** The $^{13}$C NMR spectrum of compound **10n**.

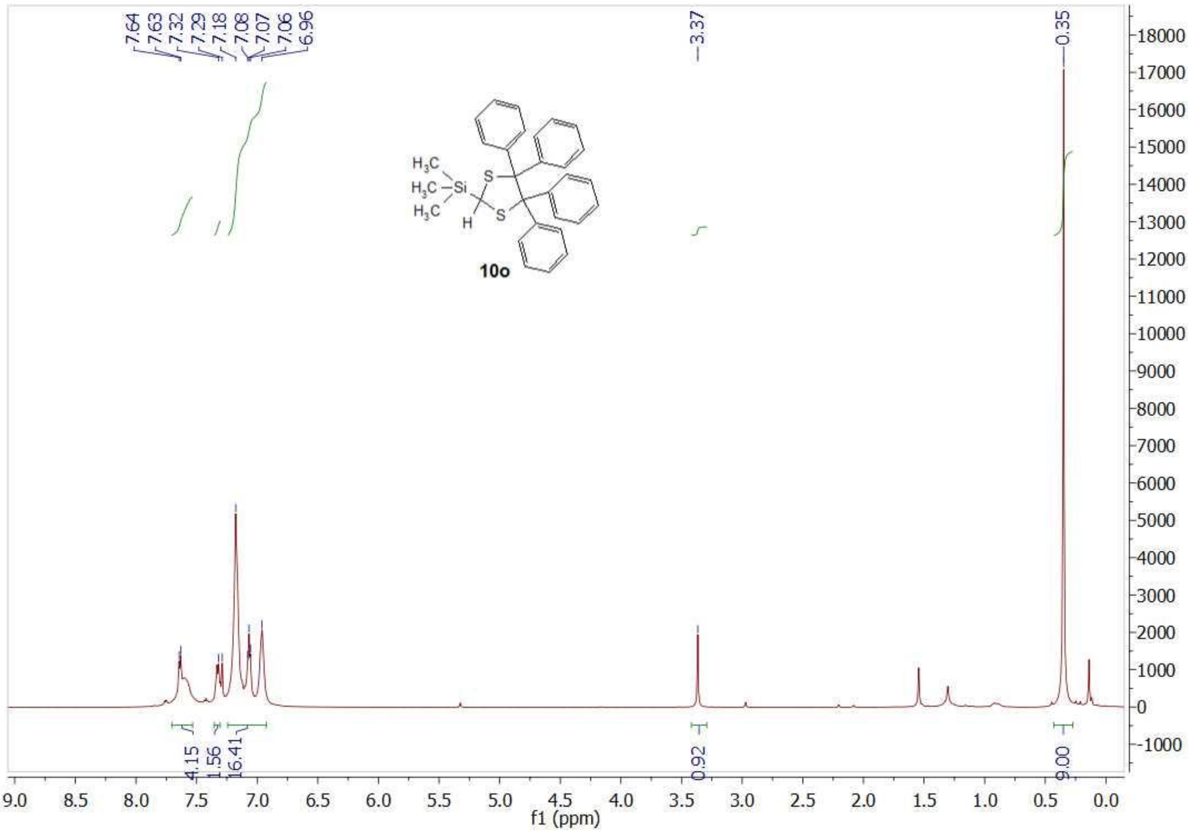


**Figure S47:** The $^{1}$H NMR spectrum of compound **10o**.

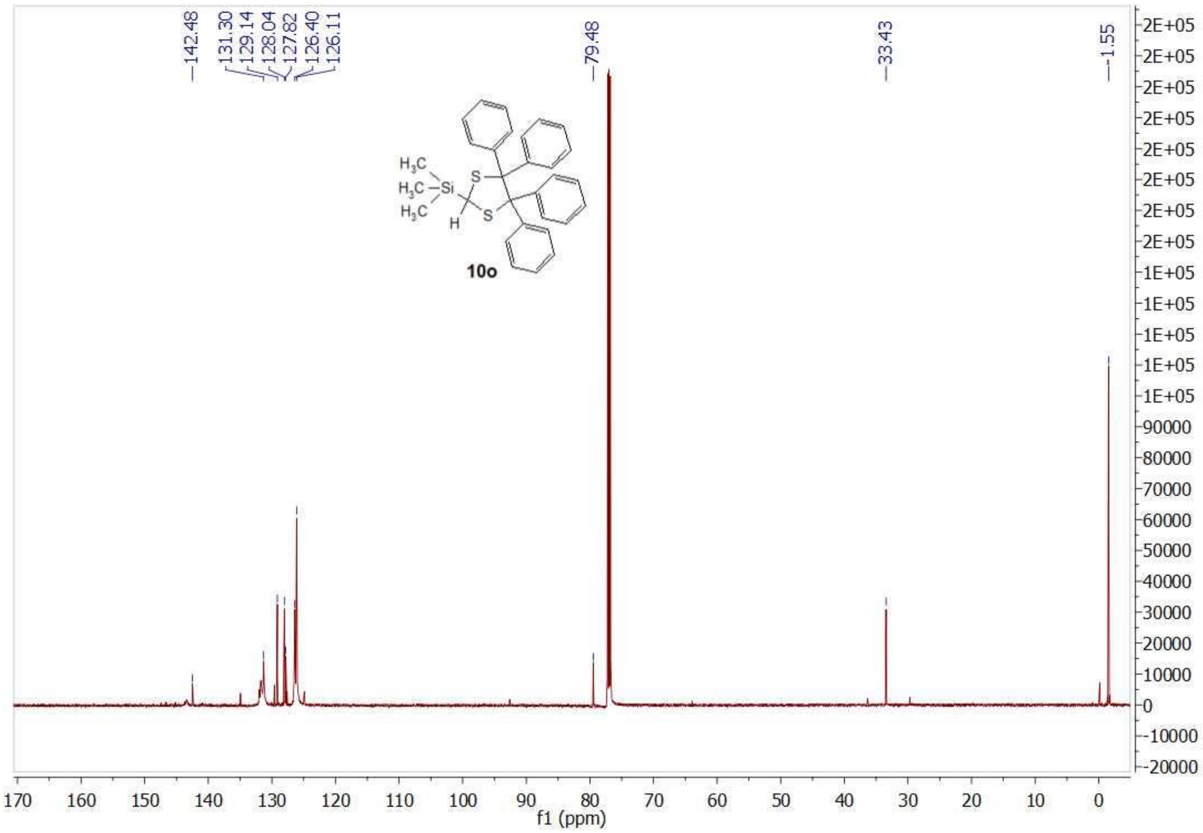


**Figure S48:** The $^{13}C$ NMR spectrum of compound **10o**.

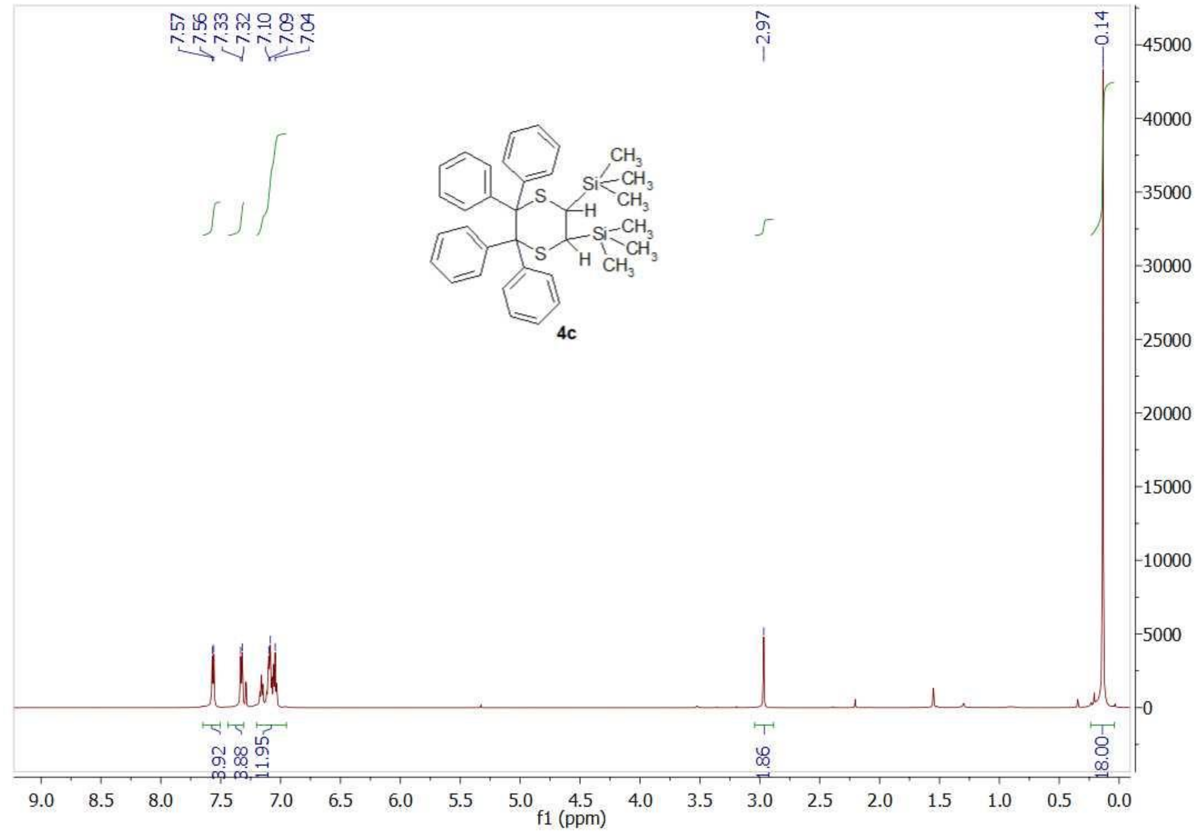


**Figure S49:** The $^{1}H$ NMR spectrum of compound **4c**.

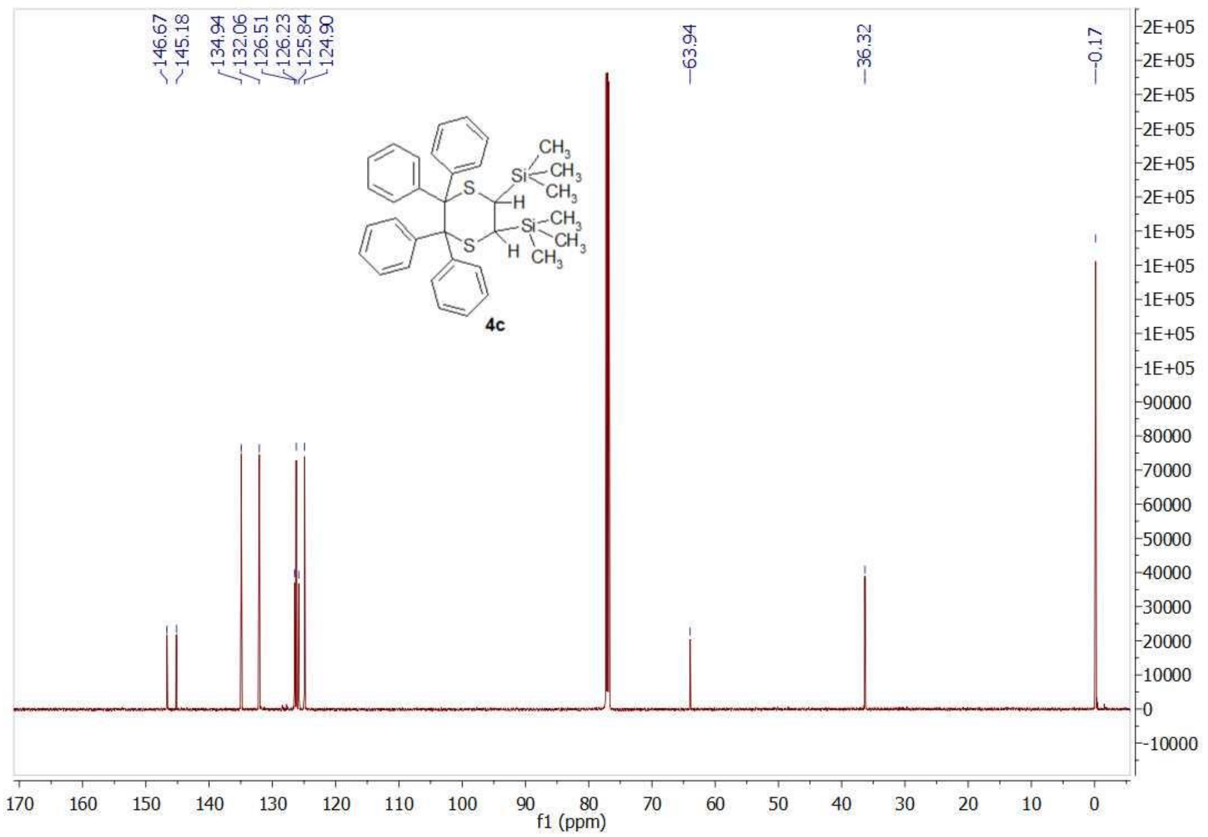

**Figure S50:** The $^{13}C$ NMR spectrum of compound **4c**.

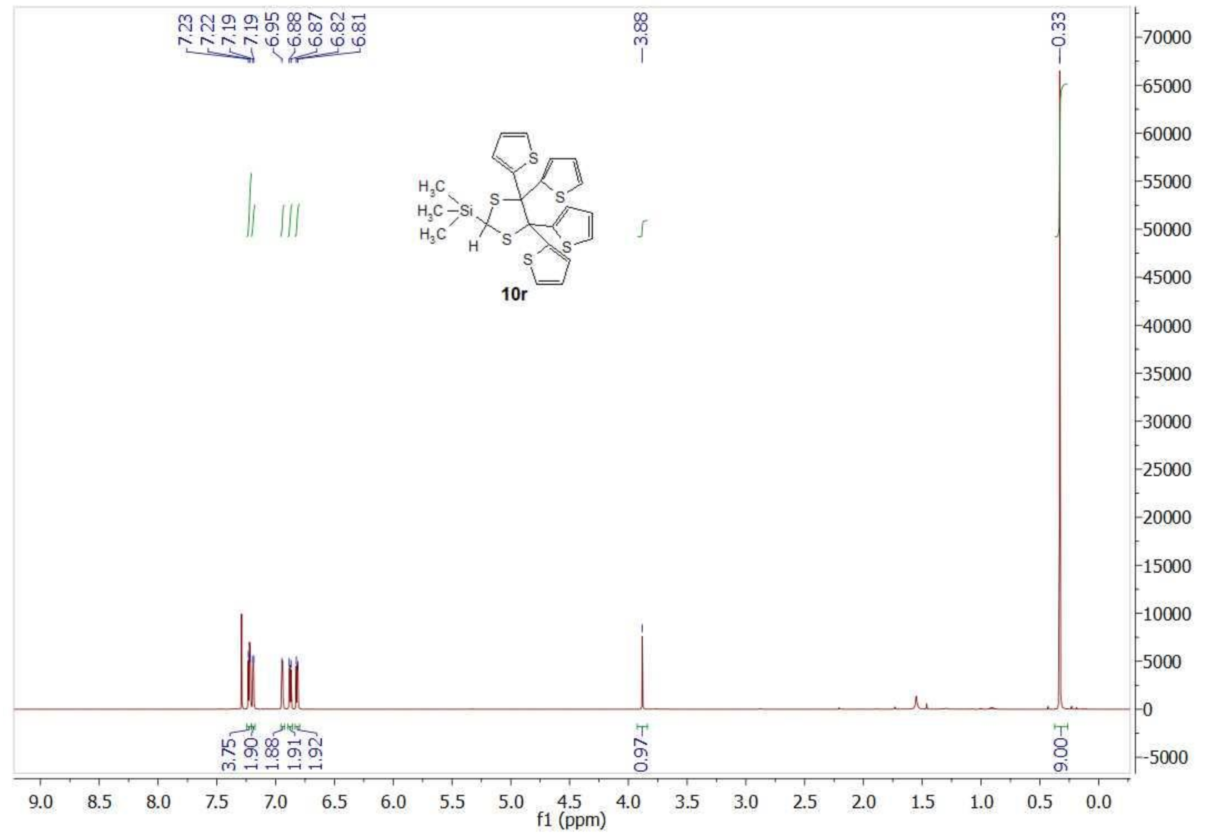

**Figure S51:** The $^{1}H$ NMR spectrum of compound **10r**.

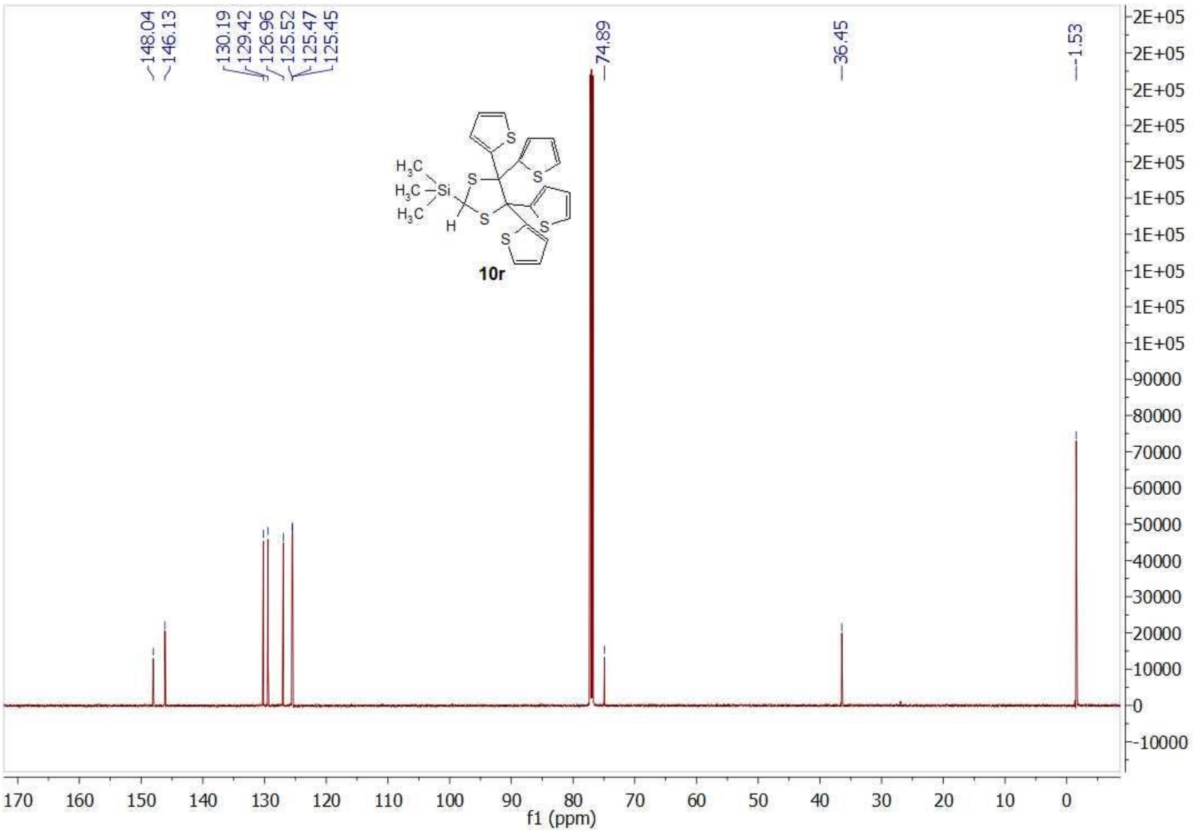


**Figure S52:** The $^{13}C$ NMR spectrum of compound **10r**.

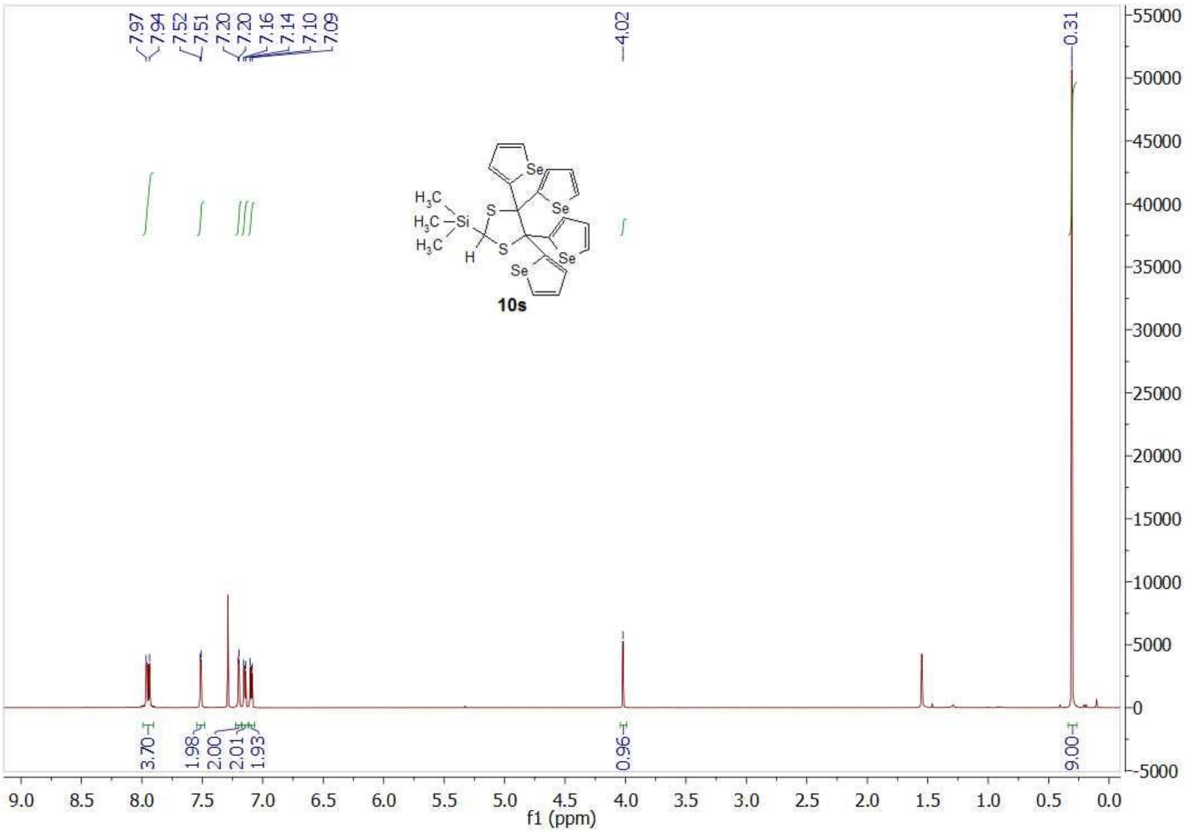


**Figure S53:** The $^{1}H$ NMR spectrum of compound **10s**.

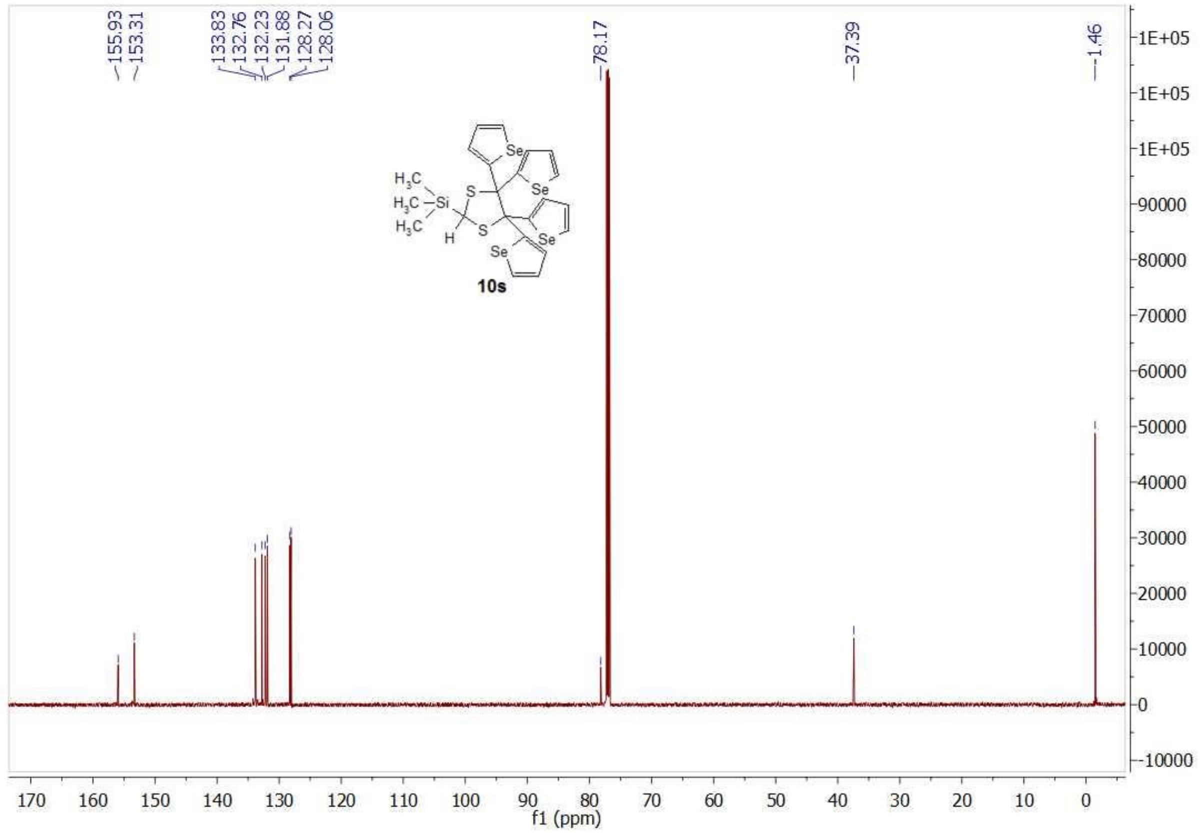


**Figure S54:** The $^{13}$C NMR spectrum of compound **10s**.
